# *CCDC66* frameshift variant associated with a new form of early-onset progressive retinal atrophy in Portuguese Water Dogs

**DOI:** 10.1038/s41598-020-77980-5

**Published:** 2020-12-03

**Authors:** Leonardo Murgiano, Doreen Becker, Courtney Spector, Kendall Carlin, Evelyn Santana, Jessica K. Niggel, Vidhya Jagannathan, Tosso Leeb, Sue Pearce-Kelling, Gustavo D. Aguirre, Keiko Miyadera

**Affiliations:** 1 Department of Clinical Sciences and Advanced Medicine, School of Veterinary Medicine, University of Pennsylvania, Philadelphia, PA USA; 2 Institute of Genome Biology, Leibniz Institute for Farm Animal Biology, Dummerstorf, Germany; 3 Institute of Genetics, Vetsuisse Faculty, University of Bern, Bern, Switzerland; 4 Optigen, LLC, Ithaca, NY USA

**Keywords:** Animal breeding, Genetic association study, Genetic linkage study, Genetic markers, Genotype, Medical genetics, Mutation, Sequencing, DNA sequencing, Next-generation sequencing, Genetics research, Protein transport, Genetics, Molecular biology, Eye diseases, Retinal diseases

## Abstract

Aberrant photoreceptor function or morphogenesis leads to blinding retinal degenerative diseases, the majority of which have a genetic aetiology. A variant in *PRCD* previously identified in Portuguese Water Dogs (PWDs) underlies prcd (progressive rod-cone degeneration), an autosomal recessive progressive retinal atrophy (PRA) with a late onset at 3–6 years of age or older. Herein, we have identified a new form of early-onset PRA (EOPRA) in the same breed. Pedigree analysis suggested an autosomal recessive inheritance. Four PWD full-siblings affected with EOPRA diagnosed at 2–3 years of age were genotyped (173,661 SNPs) along with 2 unaffected siblings, 2 unaffected parents, and 15 unrelated control PWDs. GWAS, linkage analysis and homozygosity mapping defined a 26-Mb candidate region in canine chromosome 20. Whole-genome sequencing in one affected dog and its obligatory carrier parents identified a 1 bp insertion (CFA20:g.33,717,704_33,717,705insT (CanFam3.1); c.2262_c.2263insA) in *CCDC66* predicted to cause a frameshift and truncation (p.Val747SerfsTer8). Screening of an extended PWD population confirmed perfect co-segregation of this genetic variant with the disease. Western blot analysis of COS-1 cells transfected with recombinant mutant CCDC66 expression constructs showed the mutant transcript translated into a truncated protein. Furthermore, in vitro studies suggest that the mutant CCDC66 is mislocalized to the nucleus relative to wild type CCDC66. *CCDC66* variants have been associated with inherited retinal degenerations (RDs) including canine and murine ciliopathies. As genetic variants affecting the primary cilium can cause ciliopathies in which RD may be either the sole clinical manifestation or part of a syndrome, our findings further support a role for CCDC66 in retinal function and viability, potentially through its ciliary function.

## Introduction

In the vertebrate retina, conversion of the optical image into neural impulses is initiated by rod and cone photoreceptors, whose main role is to capture and transduce photons into neuronal signals. Photoreceptors are highly polarized, light-sensitive neurons whose structure is composed of four distinct compartments: the outer segment^1^, the mitochondria-rich inner segment, the cell body harbouring the nucleus, and the synaptic body, which extends to synapse with second order neurons^2,3^. Photoreceptors are located adjacent to the retinal pigment epithelium, a cell layer that is vital for their maintenance and survival. Aberrant morphogenesis and development of rod and/or cone photoreceptors (and of their synaptic connections to second order neurons) can cause retinal degeneration (RD) that is progressive and ultimately leads to blindness. The aetiology behind RDs is often genetic in nature, making RDs predominantly inherited diseases. While past studies on RD often focused on the photoreceptors themselves, there has been increasing interest in the connecting cilium, which bridges the outer and inner segments of the photoreceptor and play a critical role in photoreceptor integrity and maintenance^4–6^. Connecting cilia have a structure analogous to primary sensory cilia in other cell types^4–6^ and inherited defects in primary cilia cause a class of inherited conditions known as ciliopathies, in which retinal dystrophy and degeneration are often, albeit not necessarily, part of a syndromic phenotype^7,8^.

The number of genes and loci associated with inherited retinal diseases, including RDs, in people is greater than 300 (RetNet: https://sph.uth.edu/RETNET/, accessed 9/24/2020) and an increasing number of comparable diseases are also recognized in non-human mammals. Indeed, variants in more than 30 genes have been associated with different forms of inherited retinal diseases in dogs, most of which are RDs and are referred to as progressive retinal atrophy (PRA) in veterinary medicine^9^ (ExpeRTs: https://www.vet.upenn.edu/experts, accessed 9/24/2020). PRA can be diagnosed in virtually any canine breed and its molecular cause may be related to one of the 30+ known breed-specific disease variants or driven by variants yet to be discovered. Investigation into the molecular basis of blinding retinal diseases in dogs is vital for the welfare and health of numerous breeds. Furthermore, dogs are invaluable animal models for the development of therapies that can be translated into a clinical setting^10–12^, providing essential insight into the molecular players and mechanisms driving RDs in humans^13–15^.

While the impact of a genetic variant on the nature and severity of pathogenesis can sometimes be inferred^16^, there are over 3000 domain of unknown function (DUF) families estimated to exist within the Pfam database^17^ representing roughly 22% of known protein families in eukaryotic organisms^18^. Characterization of DUFs and further elucidation of their structure/function relationship is an important aim in structural genomics^19,20^. As shown previously by Veermer and colleagues, utilization of mapping techniques in combination with sequencing can identify causative genes for autosomal-recessive diseases, and help pinpoint a poorly characterized putative domain associated with a pathological condition. This type of breakthrough can ultimately pave the way for discovery and characterization of new signalling pathways^21,22^.

The Portuguese Water Dog (PWD) population, although once fairly small, has gained popularity in recent years, in part due to its ‘hypoallergenic’ nature and increasing media exposure. The breed originates from three major founding events involving two different kennels^23–25^. The PWD breed offers some specific advantages over other breeds for the study of complex traits such as Addison's disease^24^, as the bottlenecks and founder effects in the current breed population is considered to have resulted in limited genetic variation^24,26,27^. Fortunately, the PWD breeding community has been highly motivated and involved in improving the health of the breed, and readily participates in genetic screening of their dogs.

PRA has been diagnosed in a variety of dog breeds and is homologous to human inherited RDs such as retinitis pigmentosa (RP). While most forms of PRA are inherited in an autosomal recessive manner, an autosomal dominant variant of rhodopsin has been reported in Mastiffs ^28^ and X-linked PRA variants have also been identified^21,29^. There are more than 38 described genetic variants that either cause independent forms of Mendelian PRAs or function as disease modifiers to alter disease expression^30–33^. Among the canine population, it is not uncommon for a given form of PRA caused by a specific genetic variant to be found in multiple breeds that share a common origin. In established breeds, it was originally thought that a single gene variant found to segregate with PRA in a given breed would likely explain most, if not all, the PRA cases in that breed due to the within-breed genetic uniformity resulting from population bottlenecks affected by founder effects and popular sires. The inter-breed ‘genetic barriers’ implemented by the breeders and breed clubs for the establishment, preservation, and enhancement of canine breeds thus contributed to keeping the variant within the breed. However, with advances in tools available for genomic and genetic analyses, some breeds are found to have more than one forms of PRA segregating in the population^14^. Examples of breeds with multiple forms of PRAs characterized at the gene mutation level include Golden Retrievers (*PRCD*, *TTC8*, and *SLC4A3*)^34–36^, Irish Setters (*PDE6B* and *C2ORF71*) ^37,38^, and Miniature Schnauzers (*RPGR* and *HIVEP3-PPT1*)^36,39^. Such findings suggest deliberate breeding practices associated with breed establishment and the subsequent breed isolation^14^ fixed multiple PRA segregating genes within these populations.

Prior to the current study, it was known that prcd-PRA (progressive rod-cone degeneration-PRA) segregated in PWDs as an autosomal recessive, late-onset PRA, recognized clinically at 3–6 years or older^14,40^ .This disease is associated with a *PRCD* genetic variant that is considered to have a relatively ancient origin and shared by nearly 60 diverse canine breeds and their crosses^36,41^ (ExpeRTs, www.vet.upenn.edu/experts, accessed 9/24/2020). Herein we report a new form of RD in PWDs that is not associated with *PRCD* and is characterized by an earlier disease onset. We performed a genome-wide association study (GWAS) and whole-genome sequencing (WGS), and identified the causative genetic variant in *CCDC66*, a gene that has been associated with RDs specifically related to defective ciliary function in mouse models as well as in another form of canine RD^42^.

## Results

### Phenotypic characterization of early-onset PRA (EOPRA) in PWDs

**Figure 1:**
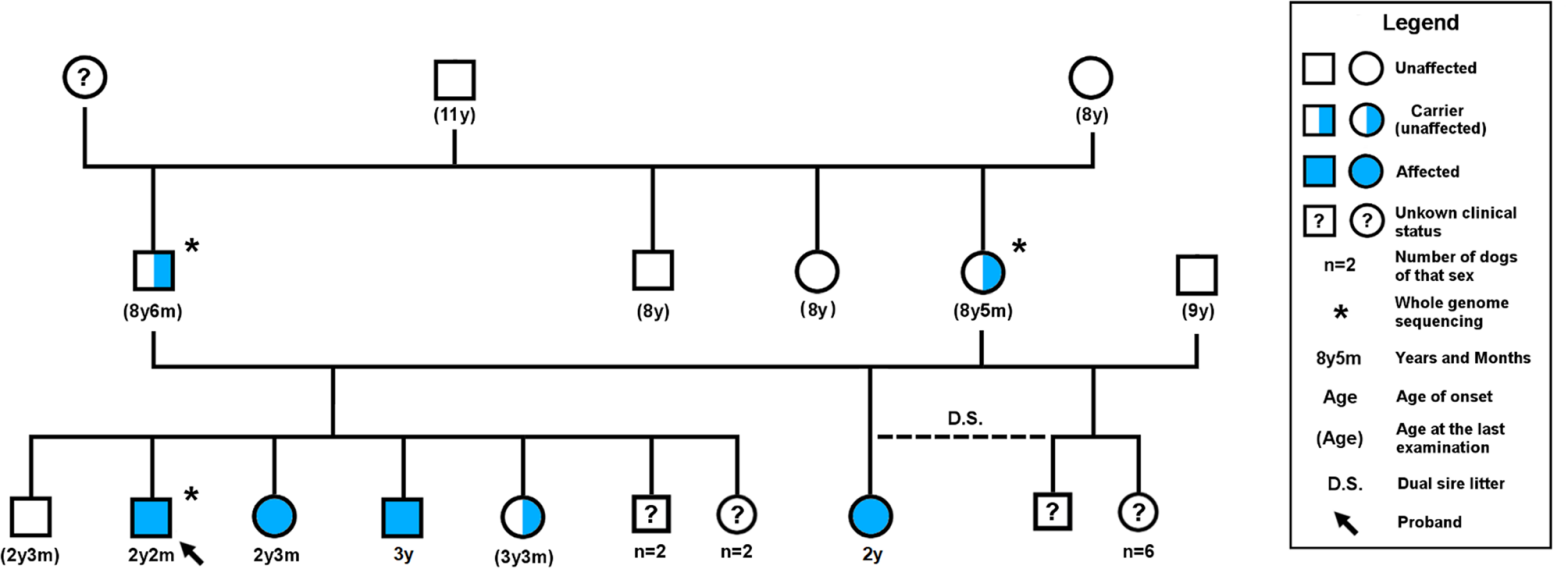
Family tree of the four affected PWDs and their close relatives. The dogs indicated with the blue filled symbol are clinically affected with EOPRA and are homozygous for the insertion allele (CFA20:g.33,717,704_33,717,705insT, CanFam3.1) in *CCDC66*. The half-filled symbols indicate clinically unaffected carriers that included the parents who are obligate carriers, and one of the unaffected siblings later genotyped as a carrier. Unknown status is indicated with a question mark for dogs that were not examined clinically. Dogs indicated with an asterisk (*) were used in WGS. The family tree shows a common sire of the parents that are half-siblings. Females are shown as circles, males in squares.

A total of 4 cases (2 males, 2 females), all full-siblings, from a small consanguineous pedigree were diagnosed with EOPRA (Fig. 1).

**Figure 2:**
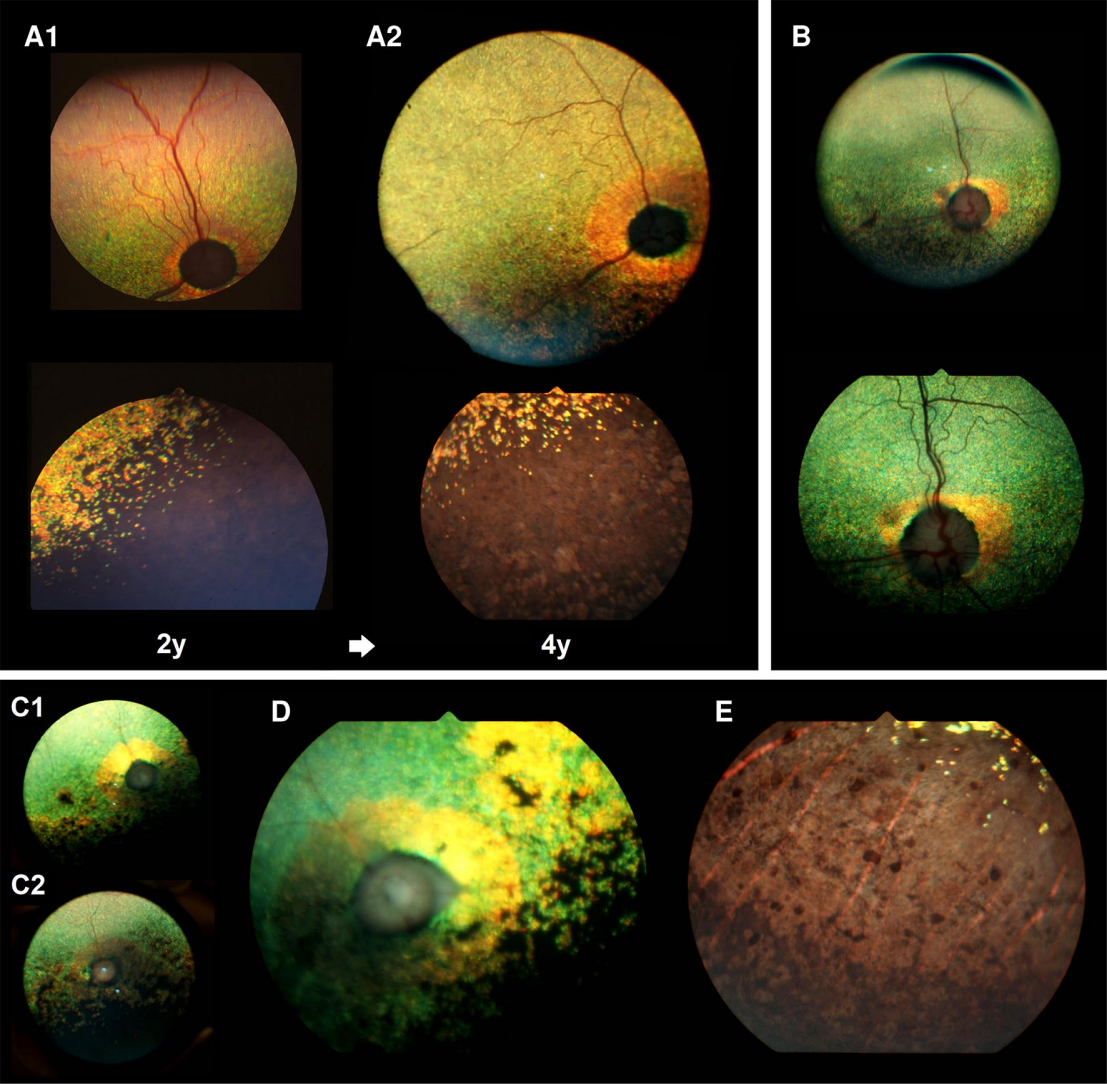
Fundus photographs of PWDs affected with *CCDC66*-EOPRA. (**A**) Peripapillary (upper images) and non-tapetal (lower images) images of an EOPRA-affected dog presenting with early- (**A1**) and mid-stage (**A2**) disease at 2 and 4 years of age, respectively. Disease stage is based on fundus appearance, with early disease corresponding to 3–4 ONL nuclei, mid disease corresponding to 1–2 ONL nuclei, and late disease corresponding to 0–1 ONL nucleus^43^. The ring-like hyperreflectivity of the peripapillary tapetal region has expanded over time. There is also progression of the multifocal depigmentation in the non-tapetal region. (**B**) Fundus photographs of an EOPRA-affected dog presenting mid-stage disease at 6 years of age. This dog was reported to have normal vision but exhibited early changes of retinal degeneration at 3 years of age. (**C**) Late-stage disease fundus of the right (**C1**) and left (**C2**) eyes in an affected dog at 4 years of age, corresponding to 0–1 ONL nucleus^43^. (**D**) Details of the peripapillary region in the right eye shown in (**C**) with distinct, ring-like hyperreflectivity, vascular attenuation, optic disc pallor, and pigment clumping. (**E**) Details of the non-tapetal fundus in the left eye shown in (**C**) with extensive depigmentation (pale brown-grey), intraretinal pigment clumping, and visualization of the choroidal vasculature. Fundus images were obtained using the Genesis fundus camera by direct photography or indirect photography through a 20D condensing lens^44^.

The initial clinical signs noticed in affected dogs by the owners were visual deficits, including difficulty following moving objects and walking into still objects, which were reportedly worse under dim light, consistent with nyctalopia. These signs became progressively worse, compromising the animals’ vision under both dim and well-lit conditions. The age of onset was determined by the time point at which the visual deficits became noticeable to the owners or when ophthalmoscopic abnormalities were first noted. The male proband and the two affected females had decreased vision per the owner at initial presentation and were diagnosed ophthalmoscopically as EOPRA with an age of onset at 2 years. A second male dog had no obvious visual deficit per the owner at initial presentation at age 2 years and had unremarkable fundus when examined ophthalmoscopically. However, peripapillar changes suggestive of PRA developed by 3 years of age at which time electroretinography (ERG) was recommended but declined. This dog was re-examined at 6 years of age when visual impairment was evident, ERGs were undetectable (Supplementary Fig. 1), and ophthalmoscopic changes were consistent with mid-stage disease. The ophthalmoscopic changes observed were common in all affected dogs, characterized by generalized tapetal hyper-reflectivity, diffuse vascular attenuation, optic disc pallor, and multifocal depigmentation of the non-tapetal fundus (Fig. 2, images by the authors).

A feature that was unique to this disease in all affected dogs was a distinct peripapillary ring of hyper-reflectivity or peripapillary conus (Fig. 2A1,B), which progressed into a broader zone of hyper-reflectivity around the optic disc in advanced disease (Fig. 2A2,C,D; details of the non-tapetal fundus in Fig. 2E). Other than the visual deficits, fundus abnormalities, and a sluggish pupillary light reflex, the remainder of the ophthalmic exam was within normal limits except for cataract formation with equatorial vacuoles in one dog at an advanced stage of disease. Functional assessment of the retina was carried out in one of the affected dogs (male dog, onset at 3 years) at a single later follow-up time point at age 6 years revealing undetectable scotopic and photopic ERGs (Supplementary Fig. 1). Of note, no other clinical abnormalities were identified or reported in the dogs affected with EOPRA.

Pedigree analysis suggested an autosomal recessive mode of inheritance based on the phenotypically normal parents of the affected dogs, with both male and female affected individuals (Fig. 1). The pedigree information revealed a recent common ancestor which was the shared sire of the obligatory carrier parents who were half-siblings. However, subsequent molecular analysis revealed that this recent common ancestor did not carry the disease allele, suggesting that the disease variant may be present more widely across the PWD population.

### Mapping of the critical candidate region

**Figure 3:**
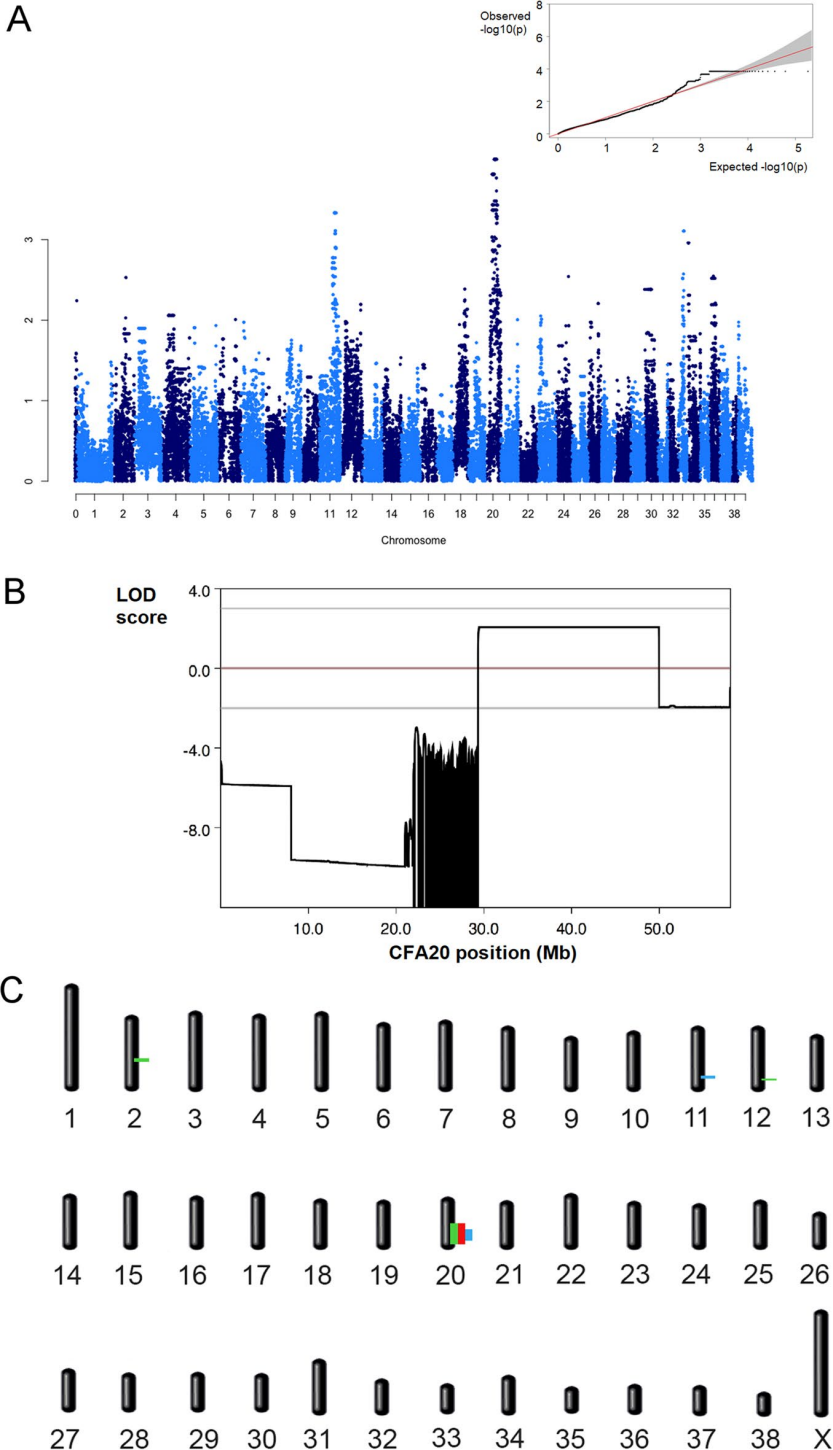
Genome-wide mapping of EOPRA. (**A**) Results of the genome-wide association study (GWAS) obtained from analyzing the Illumina SNP chip data showing the negative log of the raw *p* values calculated with the genotypic association test (minimum *p* value = 0.0001400861). Top right, QQ-plot showing the expected quantiles (x axis) of the log *p* values versus the observed (y axis). The moderate skewing of a marker toward the upper side suggests a weak association with the “affected” condition compared with what would be expected by mere chance. The flat-line at expected − log(*p* value) = 3.85 (highest *p* value reported of 0.00014) is likely caused by the high stratification thus justifying the complementary mapping methods used. (**B**) Multipoint parametric linkage carried out with a subset of the data representing a nuclear family of dogs consisting of 2 parents, 4 affected and 2 unaffected full siblings. Positive LOD scores (2.501) were found on canine chromosome 20 with a critical interval of 20.63 Mb (position 29.33–49.96 Mb). (**C**) Recap of the different mapping hits obtained through the different methods used. Genome regions identified by positive LOD scores in linkage analysis, by homozygosity mapping, and by the highest peaks in GWAS are indicated in green, red, and blue, respectively. Note the triple hit on canine chromosome 20.

Typing for the known *PRCD* disease variant did not reveal the mutant *PRCD* allele in any of the PRA-affected PWDs in the pedigree, suggesting a new inherited disease. Hypothesizing a simple Mendelian recessive inheritance, 4 cases and 19 controls derived from both closely and distantly related PWD dogs were genotyped using an Illumina Canine 170 k SNP chip. After removing 103,281 non-informative markers (due to the closely related nature of the population), 70,380 SNPs were used for GWAS mapping. The estimated lambda was 1.33, showing an expected degree of population stratification. While no statistically significant association was found with the disease, we detected possible suggestive peaks on canine chromosomes (CFA) 20 (*p* value = 0.00014), and 11 (*p* value = 0.00059) (Fig. 3A). The top 100 associated markers were all on CFA20. Due to the stratification and low power of the results, these GWAS hits were only considered as indicative, and therefore we opted to undertake additional mapping strategies.

As the first additional mapping approach, multipoint parametric linkage was carried out in a subset of closely-related PWD dogs (2 parents, 4 affected and 2 unaffected full-siblings). This revealed positive LOD scores on CFA2, CFA12 and CFA20. For the purpose of mapping, intervals with positive LOD scores and the α = 1 were considered relevant. A 20.63 Mb interval on CFA20 (CFA20: 29.33–49.96 Mb) was found to have the highest LOD score of 2.501 (Fig. 3B). The maximum LOD scores on CFA2 (2.43 Mb interval, position 54.97–57.40 Mb) and CFA12 (1.96 Mb interval, position 64.06–66.02 Mb) were 0.914 and 1.190, respectively.

As we hypothesized monogenic recessive inheritance based on the observed disease segregation, we expected that the disease allele and flanking chromosomal segments in the affected animals would be identical by descent (IBD). Therefore, we searched for extended regions of homozygosity (> 1 Mb) with simultaneous allele sharing. The 4 cases that were full siblings shared a homozygous region on CFA20, spanning from 21,911,990 (BICF2G630233682) to 48,461,664 bp (BICF2P389157).

### Variant detection and *CCDC66* annotation

Combining all the information from the different mapping strategies, the interval on CFA20 shared between all 4 cases was deemed the most likely to be associated with the disease (Fig. 3C). Hence the search for disease variants was thereafter focused on the 26.55 Mb critical interval on CFA20 (using the last heterozygous markers before and after the homozygous segment, BICF2G630233693 and chr20_48467358, ultimately CFA20:21,904,057–48,467,358 bp) established by homozygosity mapping. The variant calls resulting from WGS of three selected PWDs (1 affected dog and 2 obligate carrier parents) were filtered from an extended database of the Dog Biomedical Variant Database Consortium (DBVDC)^45^. Additionally, we checked for the presence of the variant in the idog (http://bigd.big.ac.cn/idog/, accessed 9/24/2020) and EVA databases (European Variant Database, see methods). The variants exclusive to the three PWDs in our WGS dataset amounted to 33,205.

**Figure 4:**
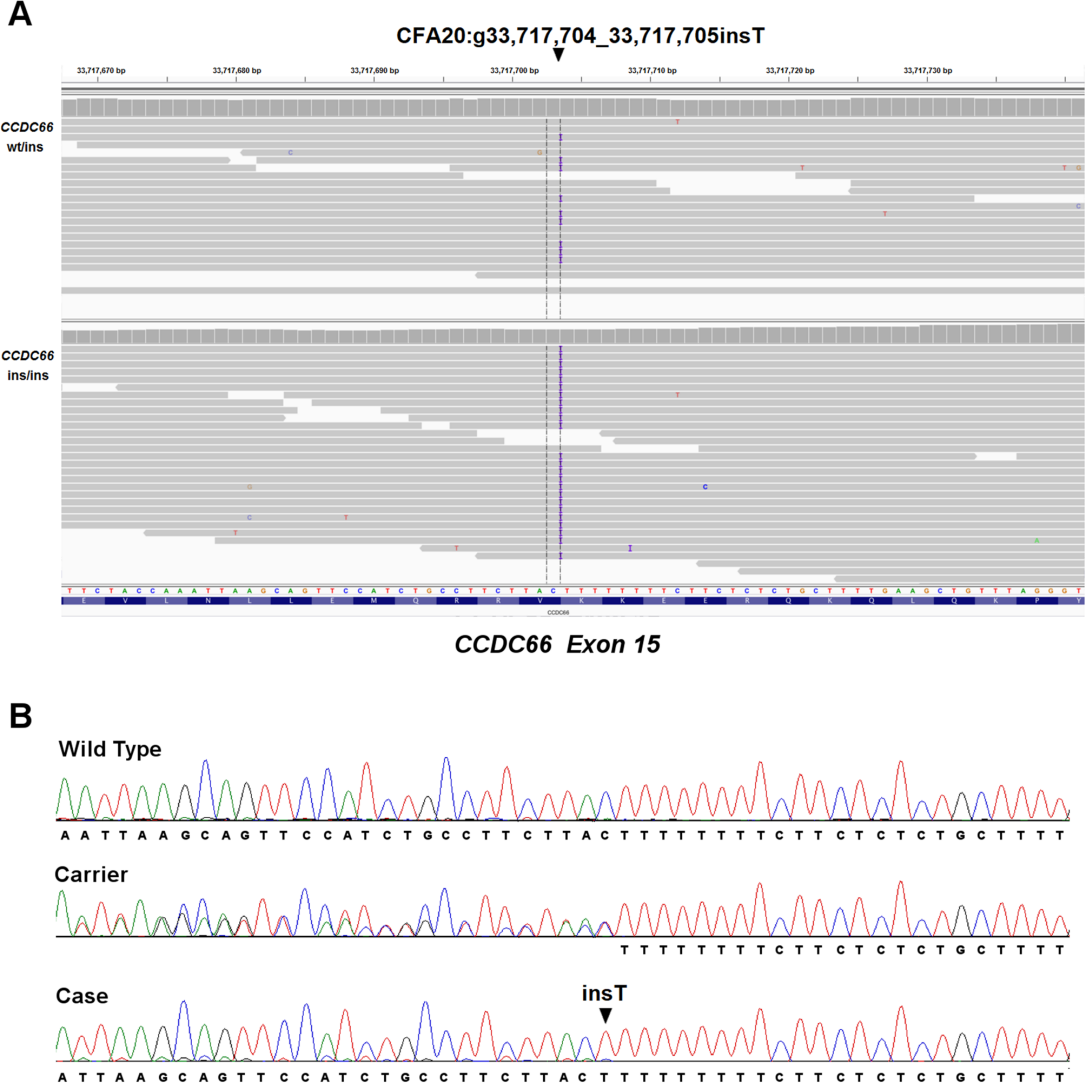
Whole-genome and Sanger sequencing of the disease variant. (**A**) Screenshot of the whole-genome sequencing (WGS) reads of an obligate carrier (parent) at the top and a clinically affected PWD at the bottom. Reads are mapped against the canine reference sequence (CanFam3.1). Note the 1-bp insertion in CFA20:g.33,717,704_33,717,705insT (CanFam3.1) (arrowhead). (**B**) Sanger sequencing confirmed the presence of the homozygous 1 bp insertion in the case. Electropherograms of a control PWD (wild type), one of the parents of the affected dogs (carrier), and an affected dog (case) are shown. The position of the variant is indicated with an arrowhead. Sanger sequences were produced with a primer in reverse complementary orientation to the genome reference sequence. Therefore, the sequence of the carrier has overlapping signals on the left side of the heterozygous indel.

Through filtering of the variants according to the assumed autosomal recessive inheritance mechanism, we found that 158 of these variants were homozygous in the affected dog and heterozygous in the obligate carriers. Finally, we searched for the variants predicted to have an impact on the coding sequence of the protein. The resulting two remaining variants were an insertion (CFA20:g.33,717,704_33,717,705insT, CanFam3.1) in the *CCDC66* gene (Fig. 4), predicted to cause a frameshift (p.Val747SerfsTer8); and CFA20:g.39,560,171G > A (CanFam3.1) in the *RNF123* gene predicted to cause a point mutation (c.2755C > T, p.Arg919Cys—predicted by Polyphen2 as being “Possibly Damaging”, score 0.81, not sufficient for “Probably Damaging”, a higher probability prediction). No large insertion, deletion, duplication or inversion associated with the disease was detected by Delly (see "Materials and methods" section).

Of note, during the analysis of the WGS output, another apparently pathogenic *CCDC66* variant predicted to cause a frameshift (CFA20:g.33,745,456_33,745,457insT, CanFam3.1) also emerged based on deviation from the reference sequence. However, examination of WGS data of over 50 canine genomes in our database that were unrelated to the current study and belonged to different breeds found that the additional T residue was in fact present in a homozygous state in all genomes examined. The presence of the additional T residue was further confirmed by PCR and Sanger sequencing of genomic DNA samples from healthy PWDs. It was therefore concluded that the CanFam3.1 reference sequence did not represent the wild type canine sequence at CFA20: 33,745,456, most likely due to a sequencing error, or possibly due to the presence of a rare variant in the Boxer from which the CanFam 3.1 reference genome assembly is derived.

With the identification of the *CCDC66* disease variant in PWDs, there are at least two distinct forms of PRA, early- and late-onset, confirmed to date in PWDs. Table 1 summarizes the two forms of PRA in PWDs, along with another form of PRA which has been associated with a distinct *CCDC66* variant in Schapendoes.

**Table 1:** Summary of EOPRA in PWDs and other related diseases.

Disease names used	References	Breed	Onset (y)	Gene	Disease variant	Possible equivalent disease name in humans
prcd-PRA	^36^	PWD	3–6	*PRCD*	c.5G > A	*PRCD*-RP
EOPRA	This paper	PWD	2–3	*CCDC66*	c.2262_c.2263insA	*CCDC66*-RP, *CCDC66*-LCA
PRA	^42^	Schapendoes	4–7	*CCDC66*	c.521_522insA	*CCDC66*-RP

### Screening of the protein changing candidate variants in the canine population

The potential protein changing variants, *CCDC66* (c.2262_c.2263insA) and *RNF123* (c.2755C > T) identified in our screening, were each genotyped in 102 and 132 available PWD samples, respectively (Table 2).

**Table 2:** Distribution of *CCDC66* and *RNF123* variants in PWDs with known phenotypes.

Retinal phenotype	*CCDC66* c.2,262_c.2,263insA			Total # of dogs	*RNF123* c.2,755C > T			Total # of dogs
	wt/wt	wt/A	A/A		C/C	C/T	T/T	
EOPRA—proband and its siblings, *PRCD* unaffected, early-onset (2-3y)	0	0	4	4	0	0	4	4
PRA*—sporadic cases, *PRCD* affected, early-onset (2-3y)	3	0	0	3	3	0	0	3
PRA*—sporadic case, *PRCD* unaffected, early-onset (2y)	1	0	0	1	1	0	0	1
Normal	69	25	0	94	93	31	0	124
Total # of dogs	73	25	4	102	97	31	4	132

Note that greater number of normal dogs were screened for the *RNF123* variant in an effort to identify homozygotes of the *RNF123* variant that would allow its exclusion from disease association. *These cases were evaluated and examined by referring board-certified veterinary ophthalmologists or accredited eye specialists other than the authors.

All four EOPRA affected siblings were homozygous mutant for both the *CCDC66* (c.2262_c.2,263insA) and *RNF123* (c.2755C > T) variants, while none of the clinically normal PWDs were homozygous mutant for either variant. These two variants, separated by ~ 6 Mb, were in linkage disequilibrium among the four EOPRA cases. Genotyping for the *RNF123* variant in an extended panel of normal control PWDs did not identify any homozygotes of the variant. Hence, the *RNF123* variant could not be conclusively excluded from disease causation by screening of available population. However, while the effect of the *RNF123* non-synonymous variant remains elusive, likely disease causation by the *CCDC66* variant is supported by previous association of *CCDC66* variants with RDs in canine^42^ and murine^46^ models, and our in vitro study below demonstrating altered subcellular expression associated with the *CCDC66* variant.

We also obtained additional DNA samples of four sporadic PRA cases of PWDs not directly related to the proband, with an early age of onset at 2–3 years, atypical for prcd-PRA, and had been diagnosed by certified veterinary ophthalmologists but not clinically examined by the authors. Three of these 4 dogs were homozygous for the *PRCD* variant and none of them were found to harbour the disease haplotype on CFA20 spanning *CCDC66*. Thus, these dogs likely represent the early-extreme of the prcd-PRA spectrum. The fourth case did not harbour either the prcd-PRA or *CCDC66*-EOPRA reported herein (Table 2), indicating a unique genetic origin. The *CCDC66* variant was screened in 616 additional PWD dogs of unknown phenotype that had been submitted for DNA testing service, revealing 14 carriers and no homozygote, with an allele frequency of 1.1% (Table 3).

**Table 3:**
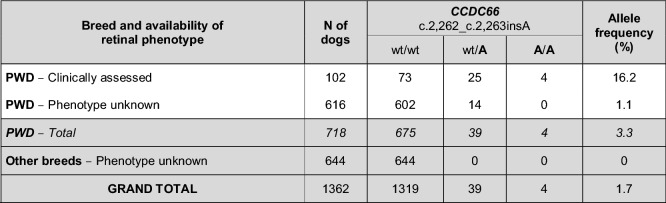
Distribution of the *CCDC66* disease variant in populations with and without phenotype information.

### *CCDC66* transcripts, protein isoforms, and impact of the variant

**Figure 5:**
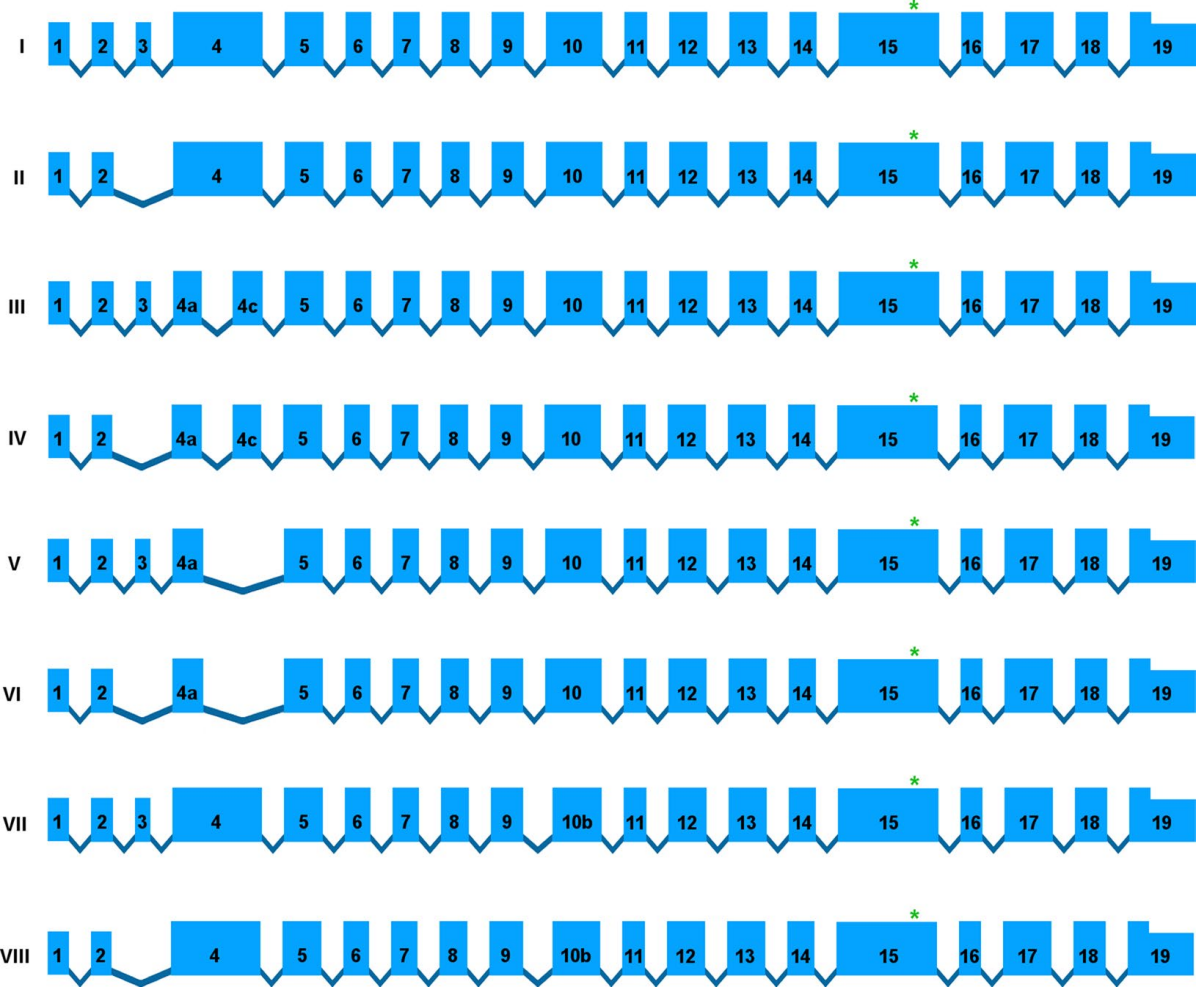
Canine *CCDC66* full-length transcript and minor isoforms. Predicted canine *CCDC66* transcript structures derived from control canine retinal RNA-seq data generated by our group for unrelated studies. Transcripts are named from I to VIII where transcripts I and II were overwhelmingly more abundant (> 90%) in our dataset. The asterisk indicates the position of the *CCDC66*-EOPRA disease variant described in this paper. The transcripts present in our RNA-seq datasets revealed 19 exons when the exitron phenomena were not taken into account.

**Figure 6:**
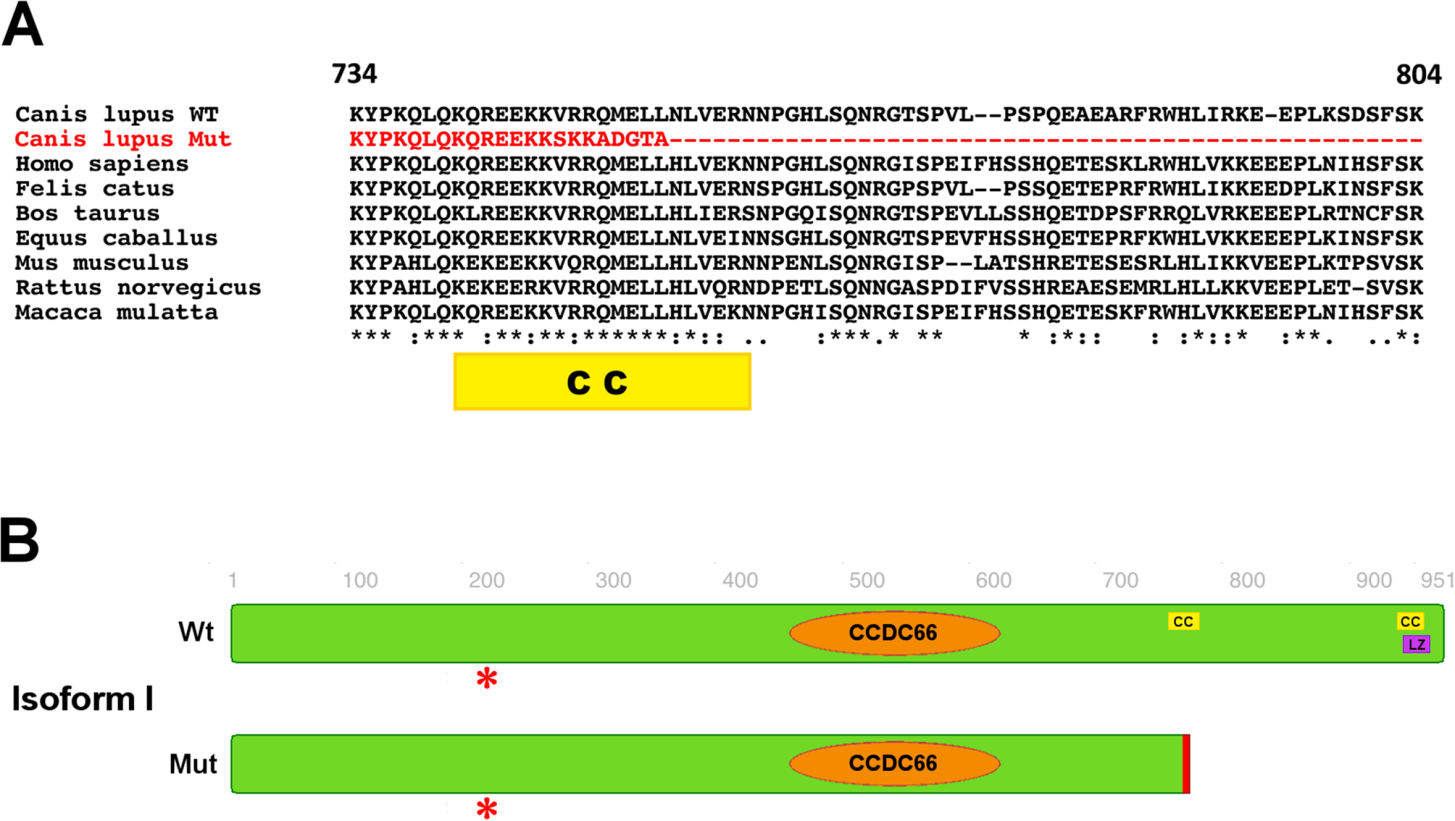
Canine CCDC66 protein structures and the implications of the disease variant. (**A**) Multiple sequence alignment of the CCDC66 protein in the region of the frameshift shows the conservation (*) of residues across mammals. On top, the canine isoform I is shown, with the positions of the extreme N- and C-terminal amino acids shown marked. The conservation (*) does not take the canine mutant (shown in red) into account. The position of the predicted coiled-coil (CC) domain is indicated with a yellow bar. (**B**) Predicted isoforms I and II in each the wild type (WT) and the mutated, truncated form (Mut). The length (AAs number) is reported in reference of the predicted isoform I. Note the CCDC66 domains, the coiled-coil (CC) and the leucine zipper (LZ) as predicted by motif identifiers. The red asterisk indicates the position of the frameshift variant described by Dekomien et al.^42^ (p.208 in isoform I). Note the loss of approximately 20% of the mutated protein and of the predicted coiled-coil and leucine zipper sequences in the C-terminal part in the mutant CCDC66 for both transcripts I and II.

The effect of the *CCDC66* variant on retinal transcripts could not be studied experimentally due to lack of access to ocular tissues from affected dogs. Therefore, we carried out extensive analysis of retinal RNA-seq data from normal dogs to first identify normal transcript isoforms and then to model how the *CCDC66* variant could impact translation. The output from Stringtie revealed eight predicted transcripts, identified as I to VIII (Fig. 5, Supplementary Data 1), in which to examine the potential impact of the variant. Transcript I was the most common at ~ 70% reads in the available data and transcripts I and II together comprised more than 90% of all detected *CCDC66* transcript reads. Note that the limited length of each RNA-seq paired-end reads at 100 bp did not allow for identification of the full transcript structure within the same reads, potentially leaving undiscovered minor transcripts. All identified transcripts were translated using the Expasy translation tool (https://web.expasy.org/translate/). Alignment was done among all eight predicted canine isoforms (Supplementary Data 2), canine isoforms with the predicted human isoforms obtained from NCBI (Supplementary Data 3), and the full-length canine wild type and mutants from other mammals (Fig. 6A, Supplementary Data 4). Taken together, these alignments reveal a predicted major heterogeneity of the N-terminus within and across species, and an overall greater abundance of conserved regions toward the C-terminus, including the region directly affected by the disease variant. The nomenclature of the disease variant is c.2262_c.2263insA for transcript I.

Translation of the mutant transcript I revealed a mutant protein (p.Val747SerfsTer8) of 755 amino-acids (aa) long, while the corresponding wild type canine isoform I predicted to be 951 aa. Thus, the variant results in a truncated mutant protein (Isoform I) that is 79.4% shorter than the wild type protein. These amino acid alignments are shown in Supplementary Data 5.

The predominant transcript I was further analyzed using Pfam^19^ to search for known or potential domains and functional sequences. While Pfam confirmed a coiled-coil domain at 450–598 aa, another alignment carried out by Interpro identified the same region as a CCDC66 domain (450–598 aa), which is conserved but not well characterized, as discussed below. The GO terms predicted were GO:0046548 retinal rod cell development; GO:0008017 microtubule binding; and GO:0005813 centrosomes. Additionally, Interpro predicted coiled-coil structures in the disease interval that are lost in the mutated protein (741–761 and 915–935 aa). Motifscan predicted a potential leucine zipper in position (918–939 aa isoform I). Psipred predicted a helix structure in the C-terminal region. The predicted wild type and mutated isoform I are shown in Fig. 6B.

### Western blot analysis of truncated mutant CCDC66 and immunohistochemical localization of CCDC66 in normal canine retina

**Figure 7:**
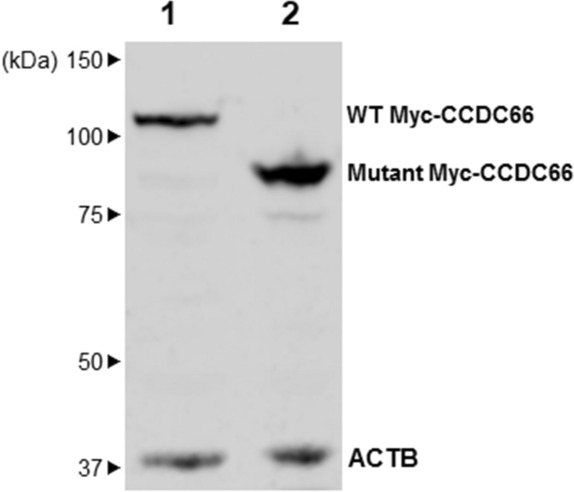
In vitro expression of wild type CCDC66 and the truncated mutant CCDC66. Western blot of lysates from COS-1 cells transfected with wild type (lane 1) and mutant (lane 2) Myc-tagged canine CCDC66. Incubation with anti-Myc antibody revealed expression of the expected full-length Myc-CCDC66 product (115 kDa) in lane 1, and a truncated protein (92 kDa) in lane 2 as expected by the disease variant. β-actin served as the internal loading control.

**Figure 8:**
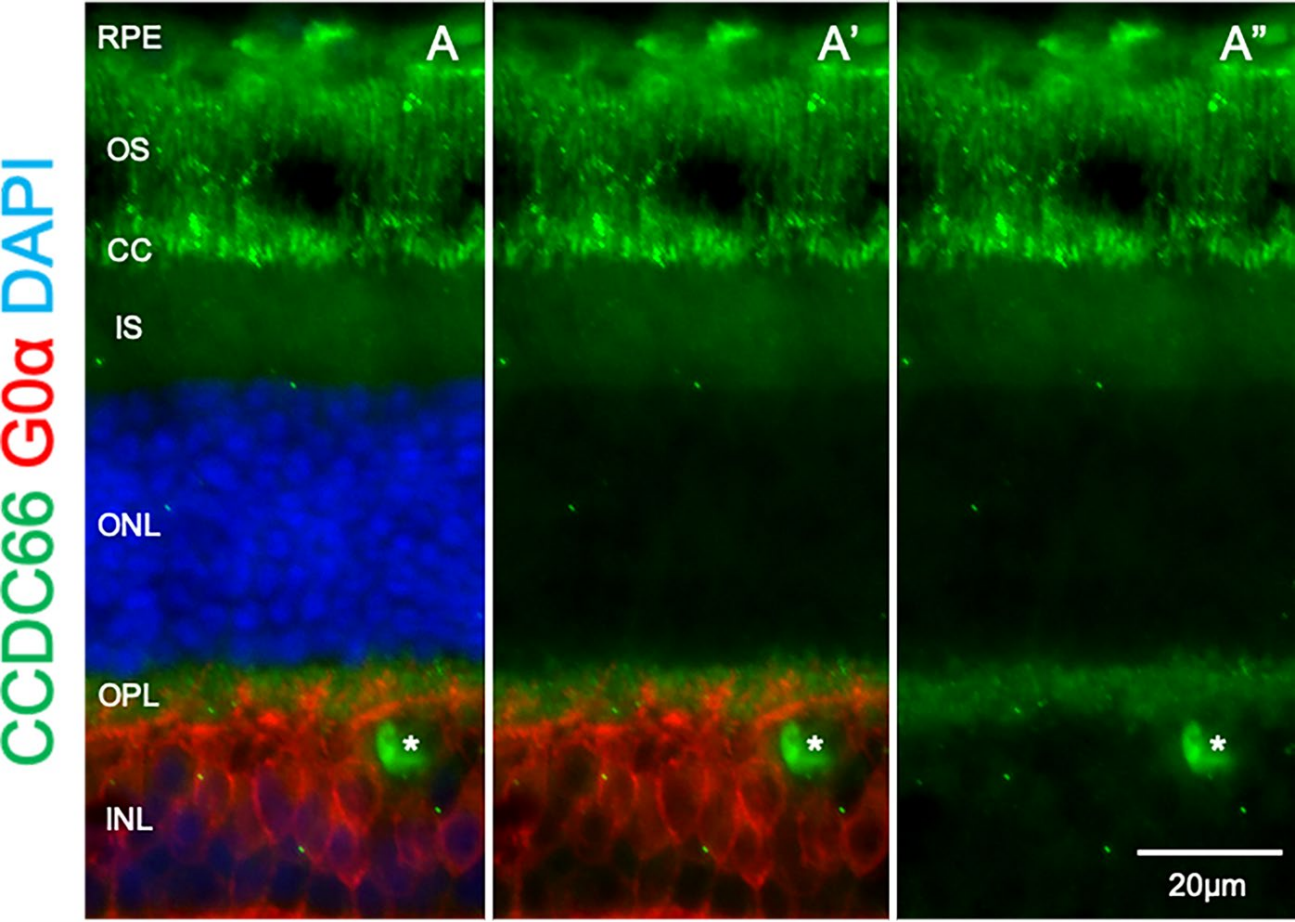
Localization of CCDC66 in the photoreceptor connecting cilia of normal canine retina. Immunohistochemistry in normal canine retinal cryosection demonstrates specific CCDC66 (green) immunolabelling of the connecting cilia with lighter labelling of the photoreceptor outer segments. There is also faint labelling along the OPL which apposes with Goα (red, ON-bipolar cell marker) indicating the localization of CCDC66 in the photoreceptor axon terminals. *blood vessel.

Western blot analysis was performed on lysates of COS-1 cells transfected with constructs encoding either the wild type or mutant CCDC66 with N-terminal Myc tags. The c-Myc (9E10) antibody was used to probe the Myc-CCDC66 protein complex. Immunoblot analysis showed bands of 115 kDa and 92 kDa (Fig. 7, Supplementary Fig. 2) corresponding to the Myc-tagged wild type and mutant CCDC66 proteins, respectively. The semi-quantitative result confirms the expression of the truncated mutant protein at levels comparable to that of the wild type. While affected canine retina was not available, we carried out immunohistochemistry in normal canine retina to validate the localization of CCDC66. Immunolabelling with anti-CCDC66 antibody revealed light labelling of the outer segment with specific and intense signals in the connecting cilia suggesting that this protein plays a role in ciliary function (Fig. 8). There was also some less intense labelling in the OPL, which was likely to be in the photoreceptor axon terminals based on apposing with Goα, an ON-bipolar cell marker.

### Nuclear mislocalization of mutant CCDC66 compared to wild type CCDC66

**Figure 9:**
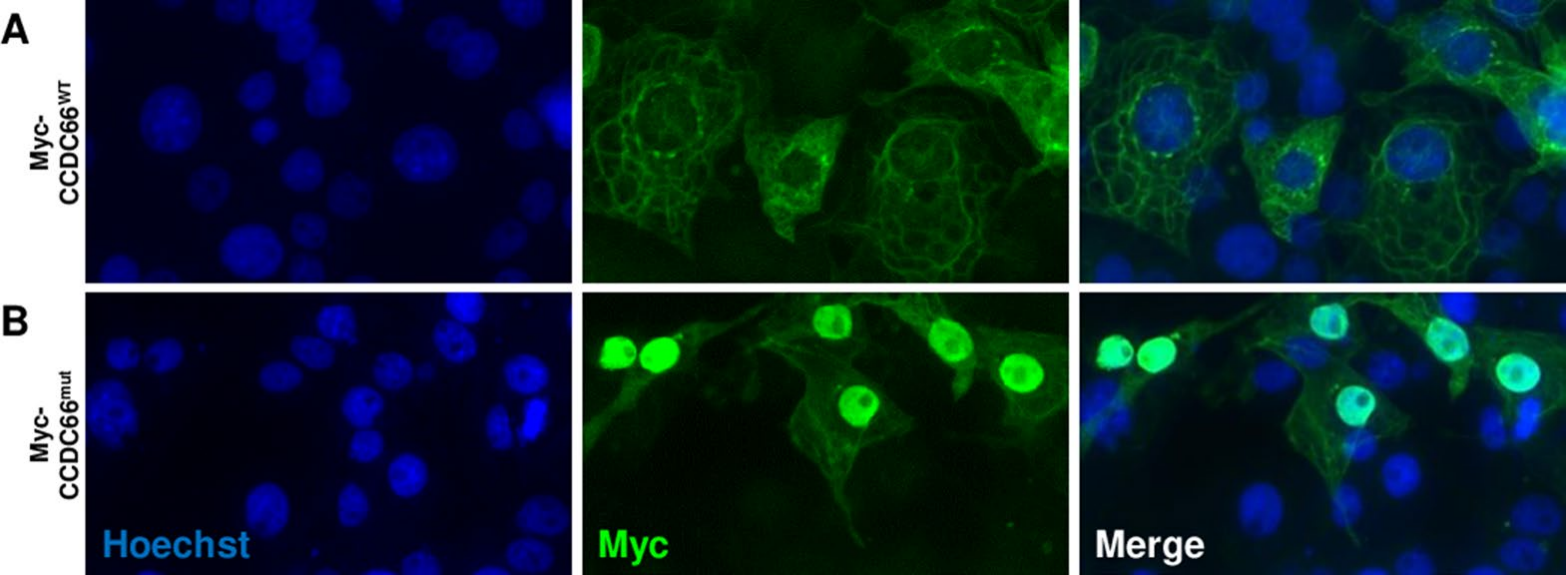
In vitro localization of wild type CCDC66 and the truncated mutant CCDC66. Immunocytochemistry shows wild type Myc-CCDC66 expressed in the cytoplasm with punctate perinuclear labelling, and no apparent nuclear localization (**A**). In contrast, expression of the mutant Myc-CCDC66 is largely restricted to the nucleus with faint cytoplasmic expression (**B**).

To examine the subcellular localization of CCDC66 proteins, wild type and mutant canine *CCDC66* cDNAs were each cloned into expression vectors with N-terminal Myc tags. COS-1 cells were transfected with each of these constructs individually, fixed, and probed with anti-Myc antibody. Immunocytochemistry revealed that wild type Myc-CCDC66 was expressed in the cytoplasm with a faint reticular pattern and punctate perinuclear labelling, with no apparent labelling in the nuclei (Fig. 9A). In contrast, expression of the mutant Myc-CCDC66 was largely restricted to the nucleus, with faint cytoplasmic expression (Fig. 9B). These findings suggest that mislocalization of CCDC66 in disease retinas can affect its role in the photoreceptor sensory cilia.

## Discussion

Through a combined approach of SNP genotyping, WGS, and population screening, we have identified a genetic variant in *CCDC66* associated with a newly characterized canine RD, namely EOPRA, in PWDs. *CCDC66* variants have been associated with RD in mice^46^ and dog^42^, supporting its functional role in EOPRA. We have shown that the *CCDC66* variant can give rise to a truncated protein that mislocalizes to the nucleus, further indicating that its altered function contributes to the disease phenotype. While a second genetic variant in *RNF123* that was in linkage disequilibrium with the *CCDC66* variant was also identified in the family of the proband, *RNF123* variants has not previously been associated with a retinal phenotype. As *CCDC66* variants have been previously implicated in retinal diseases, we believe that our newly identified *CCDC66* variant is causative for EOPRA.

Interestingly, the four affected PWDs with early onset not directly related to the proband did not harbour either *CCDC66* or *RNF123* variant. Of these dogs, three were homozygous for the prcd-PRA (*PRCD*) variant which has previously been found to segregate in the breed as late-onset PRA. As these dogs are not affected by the variant responsible for EOPRA, it suggests that these cases represent the early extreme of the spectrum of prcd-PRA. The fourth affected dog did not harbour the affected haplotype on CFA20 mapped herein or the prcd-PRA (*PRCD*) variant, suggesting that the disease in this dog is caused by an, as yet unidentified, genetic variant.

The *CCDC66* frameshift disease variant is predicted to truncate the C-terminus of the CCDC66 protein to roughly 80% of its original length. As tissues from affected dogs homozygous for the *CCDC66* variant were not available for analysis, we cloned wild type and mutant canine *CCDC66* into expression vectors for in vitro assays. Western blot results indicate that the mutated protein is translated and detectable, albeit truncated. Moreover, in vitro overexpression of CCDC66 in cultured COS-1 cells reveal altered subcellular localization in the mutant compared to the wild type, indicating a potentially defective function of the mutant CCDC66.

While the identified disease-associated genetic variant affects the C-terminus of CCDC66, the functional domains within this protein remain virtually unknown. Search for motifs based on the canine CCDC66 sequence using algorithms such as pfam, Motifscan, Interpro and Psipred confirmed the presence of the CCDC66 domain, upstream of the disease variant. These software programs also identified additional recognizable patterns (i.e. helix structures, coiled-coils and leucine zippers) some of which were missing in the truncated mutant protein. Alignment of the amino acid sequences with human CCDC66, as well as with CCDC66 in other mammals (Fig. 6; Supplementary Data 3 and 4), show a degree of conservation in the C-terminus. Taken together, these data suggest that the C-terminus of the CCDC66 protein has important functional relevance that is impacted by the frameshift variant.

Although we recently identified EOPRA as a new form of PRA in PWD, prcd-PRA caused by a genetic variant in *PRCD*^36^ has been known to affect the breed for over 10 years. Of particular note, prcd-PRA, which is categorized as late-onset PRA, is the most widespread form of PRA, having been reported in nearly 60 canine breeds and their crosses. While the associated *PRCD* genetic variant is identical across the many diverse breeds, the age of disease onset varies by breed, indicating that the genomic background unique to each breed affects disease progression. However, within individual breeds, the age of onset is much more predictable and the typical clinical disease onset of prcd-PRA in PWDs is 3–6 years or older^14,40^. Using the commercially available DNA test to detect the *PRCD* variant, the PWD breeding community has been encouraged to avoid breeding choices that can produce prcd-PRA affected puppies.

The four PWD cases in which we identified a new *CCDC66* disease variant (c.2262_c.2263insA) herein had an early disease onset at 2–3 years. Meanwhile, another variant in *CCDC66* associated with PRA in Schapendoes dogs^42^, in which a homozygous 1-bp insertion in exon 6 (c.521_522insA) leads to an immediate stop codon, had a disease onset of 4–7 years of age. Such difference in disease expression could be driven by the specific functional impairment caused by each *CCDC66* genetic variant or by difference in breed-specific genomic backgrounds and environmental factors that modify the phenotype. Dekomien et al. suggested that the *CCDC66* variant in Schapendoes was expected to truncate the CCDC66 protein^42^. In the case of PWDs, we were able to detect a truncated protein encoded by the mutant CCDC66. These results indicate that the mutant CCDC66 derived from the *CCDC66* variant in PWDs—occurring further downstream than in Schapendoes—is not subject to non-sense mediated decay and instead gives rise to a detectable amount of protein.

Dekomien and colleagues also studied a genetically-modified *Ccdc66*−/− mouse model that lacked *Ccdc66* expression and had a retinal phenotype. Their studies also indicated that CCDC66 protein primarily exists as dimers and that the coiled-coil domain may participate in protein–protein interactions^42^. Remarkably, Gerding et al. demonstrated that *Ccdc66* knockout in mice leads to early photoreceptor degeneration, with progressive retinal phenotype and physiological impairment of the retina^46^. ERG in these mice pointed to primary rod degeneration accompanied, to a lesser extent, by cone degeneration, following a time course comparable to RP in humans rather than other more rapidly progressive RP mouse models. In a more recent study, a substantial olfactory nerve fiber degeneration and alteration of olfaction-related behavior was described in aged *Ccdc66−/−* mice^47^. As both photoreceptors and olfactory sensory neurons that undergo degeneration are ciliated, *Ccdc66*−/− mice appear to represent a ciliopathy-like disease. Indeed, early-onset retinal dystrophies and degeneration are often part of the syndromic phenotype of ciliopathies^7,8,48^.

Despite being identified in 2010^42^, the potential functions of CCDC66 are only beginning to surface. Notably, CCDC66 is required for efficient ciliogenesis and centriolar satellite distribution^48^. Co-immunoprecipitation experiments demonstrated that CCDC66 interacts with numerous proteins that function in ciliogenesis (i.e. CEP72, CEP290 and PCM1) and CEP290 has also been previously implicated in retinal degeneration in human ciliopathies and mouse models^49^. Overexpression of CCDC66 disrupts organization of centriolar satellites and inhibits primary cilium formation. In our current study, in vitro expression of wild type canine CCDC66 in COS-1 cultured cells was suggestive of a microtubular expression pattern, which was compromised by the mutant canine CCDC66, resulting in its nuclear accumulation. Based on the critical role of microtubules in the integrity of photoreceptor connecting cilium, and the presence of specific CCDC66 labelling in the connecting cilia of photoreceptors in normal canine retina, our findings warrant further investigation as to whether mislocalization of CCDC66 variants may result in ciliopathies with retinal manifestations.

More recently, the role of *CCDC66* in human RDs has been challenged by Khan and colleagues, who concluded that a c.2649delA (p.Lys883AsnfsTer20) variant in *CCDC66* is not causative for the condition in patients based on the absence of the variant in one affected sibling in an affected family segregating RD^50^, as well as homozygosity in a different individual from an allegedly healthy sample pool from the Greater Middle East^51^. While variants closer to the N-terminus causing RD could not be excluded, the authors instead suggest that a genetic variant in *NMNAT1* was responsible for the RD in the patients investigated. However, our data demonstrating complete co-segregation of the *CCDC66* genotype with the EOPRA phenotype in PWDs support the essentiality of the role of CCDC66, and support *CCDC66* as suitable candidate gene underlying RDs.

Although no variants in *CCDC66* have been definitively mapped to human ciliopathies, *CCDC66* variants have been associated with RD in dogs and mouse. While mouse models often fail to completely recapitulate the human phenotype of ciliopathies^52–54^ and variants in the same genes can lead to dramatically different phenotypes across^55^ and even within^56^ mammalian species, it remains possible that as yet uncharacterized human *CCDC66* variants may still contribute to disease. Indeed, our preliminary data and previous work by others^42,46^ suggest that *CCDC66* is subject to alternative splicing events which are still incompletely characterized. Thus, the impact of genetic variants must be carefully analysed and should be weighed against the prevalence of certain transcripts specific to the tissue of interest.

Here, we show that expression of wild type canine CCDC66 in COS-1 cells results in a cytoplasmic expression pattern, but that the EOPRA disease variant accumulates within the nucleus (Fig. 9). Our findings strongly suggest that localization of the mutant CCDC66 within the nucleus impairs its physiological function, thus leading to the RD phenotype. Conkar and colleagues previously used ciliated RPE1 cells to functionally and biochemically characterize CCDC66^48^. The authors showed that CCDC66 localizes to the centrosome and the centriolar satellites, redistributing between centriolar satellites and the primary cilium in ciliated cells. Interestingly, the group also expressed the variant CCDC66 described by Dekomien^42^ and showed the mutant CCDC66 localized diffusely throughout the cytoplasm and nucleus (Fig. S2 in the Conkar paper, showing other truncated forms). The authors suggested that the RD phenotype might result from disruption of the localization and interactions of CCDC66 in cells, a conclusion consistent with our findings. Further analysis of the effect of our identified variant awaits expansion of the research colony so that affected animals/tissues can be analysed in detail to inform on the direction and approach of in vitro studies.

## Conclusions

In this paper, we show that the SNP genotyping allowing both GWAS and linkage analysis, and a combined approach with WGS is a powerful tool to study the molecular basis of inherited diseases in dog. We were able to successfully map an exonic 1 bp insertion variant that leads to a premature stop codon in *CCDC66*, a gene previously implicated in RD. Based on the expression of a truncated and mislocalized mutant CCDC66 protein in vitro, we propose this variant as causative for EOPRA in PWDs. This disease variant occurs downstream of the CCDC66 domain, the function of which remains to be characterized. Nonetheless, the interval truncated in the mutant protein is conserved in mammals and motif predictions suggest structural patterns with potential functional significance.

*CCDC66* is the second gene, after *PRCD*, in which a genetic variant has been associated with PRA in PWDs, demonstrating locus heterogeneity within a breed. In addition, allelic heterogeneity can occur between breeds, as the *CCDC66* genetic variant reported herein differs from the variant previously found associated with PRA in the Schapendoes breed.

Reports of *CCDC66* genetic variants occurring in dogs and mice have all pointed to the role of *CCDC66* in RD. Our findings in the newly characterized canine model suggest that the role of *CCDC66* in normal retinal function warrants further investigation, including functional assessment of human CCDC66 as well as screening for *CCDC66* variants in RD patients whose underlying genetic variants have not been accounted for, particularly if there are indications of ciliopathy.

## Materials and methods

### Ethical statement

The research was conducted in full compliance and strict accordance with the Association for Research in Vision and Ophthalmology (ARVO) Resolution on the Use of Animals in Ophthalmic and Vision Research. The protocol was approved by the Institutional Animal Care and Use Committees (IACUCs) of the University of Pennsylvania (#806301).

### Sample collection and phenotype assessment

All the cases affected with EOPRA (n = 4) along with control PWDs (n = 78) directly or remotely related to the cases were clinically ascertained by board-certified veterinary ophthalmologists (KM, GDA). In addition, samples from normal control PWDs (n = 20) were submitted from their owners along with detailed questionnaire related to vision and where available, eye examination results. In the exceptions (n = 4) where the animals were not available for examination by the authors, reports of PRA with onsets at 2–3 years of age from other board-certified veterinary ophthalmologists or accredited eye specialists were referred to.

All examinations were done by indirect ophthalmoscopy and biomicroscopy after pharmacologic dilation as stipulated by the ACVO guidelines for screening eye examinations. For selected cases, fundus photographs were taken using a fundus camera (Genesis-D; Kowa Ltd, Nagoya, Japan) by direct photography or indirect photography through a 20D lens^44^. Blood samples were collected in EDTA tubes for DNA extraction. For each dog, eye examination records, and where possible, pedigree information, were obtained. All the clinical information was reviewed by board-certified veterinary ophthalmologists (KM, GDA) prior to inclusion of each animal in the study. Electroretinography was carried out in one dog based on protocols previously described^57^.

### Animals and single nucleotide polymorphism (SNP) genotyping

Blood-derived genomic DNA samples from a total of 23 PWDs (4 affected and 19 unaffected) were used for SNP chip genotyping. Among these samples, there was a subset of closely related dogs consisting of the 4 EOPRA cases, their 2 unaffected full-siblings, and the 2 unaffected parents. DNA was extracted with the Illustra DNA extraction kit BACC2 (GE Healthcare) following manufacturer's instructions. Genotyping was performed using the CanineHD BeadChip (Illumina) that includes 173,661 evenly distributed SNPs, and following standard protocols as recommended by the manufacturer. Additional PWD DNA derived from blood or cheek swab samples were genotyped in-house or through the Optigen research program for the *PRCD* variant using established methods^36^ and for the *CCDC66* and *RNF123* genetic variant as described below.

### Genome-wide association

For GWAS, we used the GenABEL package (https://cran.r-project.org/src/contrib/Archive/GenABEL/, v 1.8) developed for the R Studio integrated environment^58^ (https://rstudio.com/, v 0.99.903). As a preliminary step for the analysis, we used the standard quality control settings to remove markers and individuals with call rates < 90%, markers with minor allele frequency (MAF) < 5%, and markers strongly deviating from Hardy–Weinberg equilibrium. The preliminary MDS plot and ancestry analysis expectedly pointed out a stratified population, therefore the analysis was followed by a mixed model association study as designed in the GenABEL package. The Manhattan plot was analyzed to search for suggestive or associated peaks.

### Homozygosity mapping and phasing

The GWAS analysis was followed by a homozygosity mapping approach, carried out with PLINK (https://www.cog-genomics.org/plink2 v 1.9)^59^ to detect extended intervals of homozygosity with shared alleles, and to fine-map the region containing the responsible genetic variant. The dataset consisted of 173,661 evenly spaced SNPs. Individuals and SNPs were selected using the commands “--keep”, and “--extract”. Homozygosity analysis was performed on all cases using the commands “--dog”, “--homozyg” and “--homozyg-group”.

### Linkage

To prepare the dataset for linkage analysis, the PLINK (https://www.cog-genomics.org/plink2 v 1.9)^59^ software was used with the “--dog” command to account for the species-specific chromosome quantities. The genotype data was pruned to remove SNPs that had either > 10% missing genotype calls, had a minor allele frequency of < 5%, or exceeded the Hardy–Weinberg disequilibrium *p* value of 0.0001 (“geno 0.1”, “--maf 0.05”, “--hwe 0.0001”). Subsequently, the MERLIN (https://csg.sph.umich.edu/abecasis/Merlin/download/, v 1.1.2) software^60^ was used to carry out the parametric linkage analysis under the assumption of a recessive mechanism of inheritance. The dataset consisted of 8 dogs: 6 full-siblings (4 affected and 2 unaffected) and their 2 obligate carrier parents. The “--error” command was applied to filter out Mendelian errors. The linkage for the each of the autosomes was carried out in the following manner: multipoint LOD scores was calculated using a model for mono-allelic autosomal recessive trait; we assumed for the calculation a complete penetrance and a frequency of the mutated allele of 0.68. MINX, part of the MERLIN package and tailored for X-chromosome linkage analysis, was used to analyze the X chromosome, with the same parameters previously described.

Since we analyzed a small family with a reduced number of samples (8-individuals in total), any positive LOD score obtained was accepted as possible indication of linkage. The graphical representation of the analysis was obtained in MERLIN using the “--pdf” option.

### Whole-genome sequencing

Three DNA fragment libraries were prepared from samples derived from a case-parent trio (1 affected dog and its 2 obligate carrier parents). Libraries of 300 bp insert size was prepared, llumine HiSeq2500 paired-end reads (2 × 100 bp) were collected one lane per sample, and the fastq files were created using Casava (https://bioweb.pasteur.fr/packages/pack@casava@1.8.2, v 1.8.2). A total of 1,637,397,795 reads (100 bp paired-end reads) were collected for the three dogs from a shotgun fragment library (corresponding to an approximate average 32.6 × coverage of the genome). The paired-end reads were then mapped to the dog reference genome CanFam3.1. The reads were aligned using Burrows-Wheeler Aligner (BWA, http://bio-bwa.sourceforge.net/, v 0.5.9-r16)^61^ with default settings. The SAM file which was generated by BWA was then converted to a BAM file and the reads were sorted by chromosomes using samtools (http://www.htslib.org/, v 1.1)^62^. The PCR duplicates were marked using Picard tools (http://sourceforge.net/projects/picard/).

### Variant discovery

For variant calling and data alignment, the GATK (https://gatk.broadinstitute.org/hc/en-us, v 2.4.9) program^63^ was used in the “unified genotyper” mode. The output data was produced in variant call format (vcf, version 4.0); the raw calls for all samples and sites were flagged using the standard variant filtration module of GATK; such variant filtration was executed in accordion to the “best practice” documentation of GATK (version 4). The prediction of the functional effects of the detected variants was carried out using the software SnpEff (https://pcingola.github.io/SnpEff/, v 5.0)^64^, comparing the data with the CanFam3.1 assembly . The final list of the variants exclusive to the PWD samples was obtained filtering them from the Dog Biomedical Variant Database Consortium (DBVDC), using the software BCFtools (https://samtools.github.io/bcftools/, v 1.11)^61,65^. Further filtering was carried out using the DogSD (https://bigd.big.ac.cn/dogsdv2/, accessed 9/24/2020) and the European Variation Archive variant browser (https://www.ebi.ac.uk/eva/?Variant-Browser, accessed 9/24/2020) as additional source of variants. The accession numbers of the available bam files are reported in File S8. Furthermore, the Delly2 program (https://github.com/dellytools/delly, v 0.8.5)^66^ was used to detect structural variants in the three BAM files. The same software was used to validate the suspected variants in the same cohort of the affected case and the two sequenced parents, plus 10 canine .bam files sequenced by the authors in studies unrelated to the PWD work reported herein. The commands for deletions, insertions, inversion and duplications were all executed separately. The analysis was carried out focusing on the candidate region evidenced by mapping.

### PCR and Sanger sequencing

The genetic variants in *RNF123* and *CCDC66* were verified in all the available PWD samples, by re-sequencing of targeted PCR products using Sanger sequencing. PCR products were sequenced on an ABI 3730 capillary sequencer (Life Technologies, Carlsbad, CA) after treatment with ExoSAP-IT (Thermo Fisher Scientific, Waltham, MA). Sequence data were analyzed using Sequencher (http://www.genecodes.com/, v 5.1, GeneCodes). Primer sequences are available upon request.

### Transcript characterization

The *CCDC6* mRNA was annotated using SPLIGN (https://www.ncbi.nlm.nih.gov/sutils/splign/splign.cgi) with the human transcript NM_024503.4 as template. Stringtie (https://ccb.jhu.edu/software/stringtie/, v 2.13)^67^ was used with a dataset of 8 samples of canine retinal RNA-seq produced in our lab for unrelated studies as a reference to re-annotate *CCDC66* transcripts and to detect alternate splicing. Further, the predominant, full-length canine *CCDC66* cDNA was amplified by RT-PCR and subjected to Sanger sequencing for confirmation of the exon structure and the cDNA sequence.

### Sequence alignment

The transcript sequences obtained were aligned to the predicted sequences of other mammals using Clustal Omega (https://www.ebi.ac.uk/Tools/msa/clustalo/)^68^. Furthermore, each transcript was converted into protein sequences using the Expasy translation tool (https://web.expasy.org/translate/). Each predicted protein sequence obtained was aligned using Clustal Omega against the human sequences and the conserved regions were marked. Additionally, the predicted proteins from *CCDC66* transcripts I and II were submitted to Pfam-sequence search (https://pfam.xfam.org/)^19^ for the prediction of known/potential domains and functional sequences. Additional alignments were carried out by Interpro (https://www.ebi.ac.uk/interpro/)^69^, Motifscan (https://myhits.isb-sib.ch/cgi-bin/motif_scan), and Psipred (http://bioinf.cs.ucl.ac.uk/psipred/)^69^. *RNF123* variant impact predicted with Polyphen-2 (http://genetics.bwh.harvard.edu/pph2/)^70^.

### Cloning of cDNA constructs into expression vectors and cell transfection

Retinal RNA was extracted from a wild type canine retina, and *CCDC66* cDNA was amplified by RT-PCR. Two non-synonymous variants were found in this wild type retina affecting relatively well conserved amino acids. These were corrected to match the reference sequence using NEBuilder HiFi DNA Assembly Master Mix (New England Biolabs, Ipswich, MA). The same kit was used to introduce the CFA20:g.33,717,704_33,717,705insT mutation to construct the mutant version of *CCDC66* cDNA. Full-length wild type and mutant canine *CCDC66* cDNA was each cloned into pKMyc vectors (Addgene, Cambridge, MA) to create N-terminal Myc-tagged constructs^71^. African green monkey kidney fibroblast-like cell line (COS-1) (ATCC, Manassas, VA; cat. #CRL-1650; lot #59102713) was cultured in DMEM with 10% foetal bovine serum and 10U/mL penicillin/streptomycin with an atmosphere of 5% CO_2_ at 37 °C. Cells were seeded and grown to 70–90% confluency in glass cell culture chamber slides and transfected with either of the recombinant expression vectors or a control ‘empty’ vector using Lipofectamine 3000 (Invitrogen, Thermo Fisher Scientific), according to manufacturer’s protocol. After 48 h of transfection, cells were subjected to immunocytochemistry or harvested for Western blot analysis.

### Western blot analysis

Extracts of transfected cells were prepared in RIPA buffer (Thermo Scientific, Waltham, MA, USA) containing a protease and phosphatase inhibitor cocktail (Halt, Thermo Scientific, Waltham, MA, USA). The cells were first homogenized by vortexing and left on ice for 15 min. The samples were then centrifuged and a total protein concentration in the supernatant was assessed by Bradford assay. From each sample, 60 µg of total protein was denatured at 95 °C for 5 min in 1X Laemmli sample buffer (Bio-Rad, Hercules, CA, USA) and 5% 2-mercaptoethanol. Protein samples were then resolved on a 4–12% Bis–Tris gel (Invitrogen, Carlsbad, CA, USA) in 1X SDS/Tris/Glycine buffer, transferred to a nitrocellulose membrane (Bio-Rad, Hercules, CA, USA) and blocked with 1X Odyssey PBS Blocking Buffer (LI-COR, Lincoln, NE, USA) for 1 h at room temperature. The membrane was then probed with anti-c-Myc (1:1,000) (9E10, Cell Signaling, Danvers, MA, USA) and anti-β-Actin (1:2,000) (ab8227) (Abcam, Cambridge, UK) for normalization at 4 °C for 18 h. After incubation with infrared dye-tagged secondary antibodies (LI-COR, Lincoln, NE, USA), immunoblots were washed thrice in PBS-Tween 20 (0.1%) with a final wash in PBS. Protein bands were visualized on a digital imaging system (Odyssey FC, LI-COR, Lincoln, NE, USA).

### Immunohistochemistry (IHC)

Tissue preparation, sectioning and IHC were done as previously described^72^. Briefly, cryosections were blocked for 1 h in PBS solution containing 0.25% Triton X-100, 3% normal horse serum, 1% BSA and 0.1% gelatin from cold water fish skin, followed by overnight incubation in primary antibody. After PBS washes, secondary antibody conjugated with Alexa Fluor (Molecular Probes) was incubated for 2 h at room temperature. DAPI was used as a counterstain and slides were then mounted in ProLong Glass Antifade (Invitrogen). Epifluorescence microscopy was examined with a Zeiss Axioplan microscope (Zeiss) and images were digitally captured using SPOT 4.0 software and camera. Images were arranged using graphic software ImageJ (NIH).

### Immunocytochemistry

Cells were washed with ice cold PBS and fixed in 4% paraformaldehyde for 15 min. Fixed cells were washed in PBS, permeabilized in PBS containing 0.25% Triton X-100 (PBST), and blocked for 15 min in PBST containing 5% bovine serum albumin and 4.5% fish gelatin. Fixed cells were incubated with mouse monoclonal c-Myc antibody (9E10) (Cell Center, University of Pennsylvania) overnight at 4 °C. The cells were then washed in PBS prior to being incubated with the Alexa-Fluor secondary antibody (1:200) (Invitrogen, Thermo Fisher Scientific) in the dark for 1 h. The cells were again washed in PBS and then incubated in Hoechst solution (1:1000) (Invitrogen, Thermo Fisher Scientific) to stain the cell nuclei. Cells were washed and mounted in Gelvatol mounting medium (containing polyvinyl alcohol and glycerol). Immunofluorescence was visualized with an epifluorescence microscope (Axioplan, Carl Zeiss Meditec, Oberkochen, Germany) and images were digitally captured using Spot 4.0 camera.

## Supplementary information

Supplementary Information.

## Data Availability

The whole-genome sequencing datasets generated and analysed during the current study are available in the European Nucleotide Archive (ERS4045175-77 (SAMEA6245499-501)). All data generated or analysed during this study are included in this published article (and its Supplementary Information files).
