# Supplementary material for: CCDC66 frameshift variant associated with a new form of early-onset progressive retinal atrophy in Portuguese Water Dogs
Source: Sci Rep. 2020 Dec 3;10:21162. doi: 10.1038/s41598-020-77980-5 (PMC7712861; doi:10.1038/s41598-020-77980-5)
Supplement: Supplementary file 1 — Supplementary Information. [file 41598_2020_77980_MOESM1_ESM.docx]

***CCDC66* frameshift variant associated with a new form of early-onset progressive retinal atrophy in Portuguese Water Dogs**

Leonardo Murgiano^1^, Doreen Becker^1,2^, Courtney Spector^1^, Kendall Carlin^1^, Evelyn Santana^1^, Jessica Niggel^1^, Vidhya Jagannathan^3^, Tosso Leeb^3^, Sue Pearce-Kelling^4^, Gustavo D. Aguirre^1^, Keiko Miyadera^1,^ *

^1^Department of Clinical Sciences & Advanced Medicine, School of Veterinary Medicine, University of Pennsylvania, Philadelphia, PA

^2^Institute of Genome Biology, Leibniz Institute for Farm Animal Biology, Dummerstorf, Germany

^3^Institute of Genetics, Vetsuisse Faculty, University of Bern, Bern, Switzerland

^4^Optigen, LLC., Ithaca, NY

*Corresponding author

E-mail: [kmiya@upenn.edu](mailto:kmiya@upenn.edu) (KM)

**Supplementary Information**

**
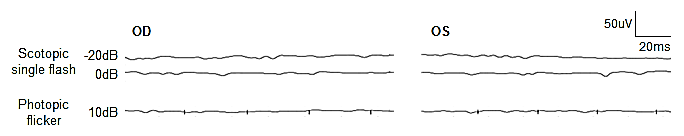
**

**Supplementary Figure 1. Electoretinogram of a Portuguese Water Dog affected with early-onset PRA.** Electroretinography traces in an affected Portuguese Water Dog recorded at 6 years of age with mid-stage retinal degeneration. The onset of visual deficit was inconclusive in this dog, sometime between 3 and 6 years of age. Traces from scotopic flashes of increasing light stimulation (-20 and 0dB) and from photopic 29Hz flicker at 10dB. No response could be detected are shown all of which were undetectable. OD, right eye; OS, left eye.

**Supplementary Figure 2.** Uncropped original image of the blot presented in Fig 7. The blot was

probed with anti-Myc and anti ACTB antibodies. Lanes 1 and 4, Lysates of COS-1 transfected

with Myc-tagged wild type CCDC66; lane 2, lysate of COS-1 transfected with Myc-tagged

mutant CCDC66; lane 3, lysate of HEK293 transfected with Myc-tagged wild type MAP9

(positive control); M, protein molecular weight marker.


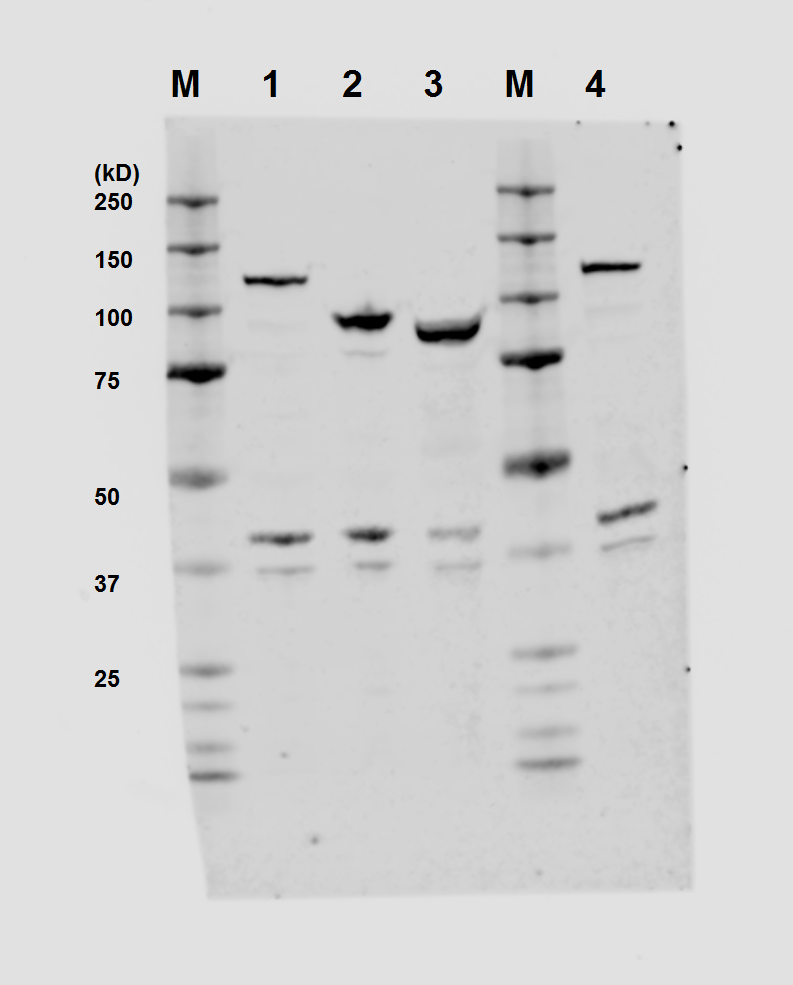


**Supplementary Data 1.** Canine *CCDC66* exon sequences including variants, mainly due to exitrons, as confirmed by canine retinal cDNA. Each predicted transcript I-VIII is also reported.

**1) CCDC66 exons, canine**

**>Exon 1**

TACACAGGTGGCTGGAGCGTCCTGTGGAGAGAGCGAGAGGTCAGGCCATGAACCTGGG

**>Exon 2**

AGATGGCTTAAAGCTTGAAACTGAATTGTTGGATGGAAAAACCAAGTTAATATTGTCTCCATATG

**>Exon 3**

AATGTAAATCAAAAAATTCTGTGAAG

**>Exon 4**

ATGGGAAGTAAGAACAAGATTGCAAAATGTCCTATAAGAACAAAACAGACTGGATACATTCTAAAATCAACACAAAATACATGTATCAGGAGTGGAAAACTTTTGCAAAAGAAGAGAATGGGTTCAGAAACTTCACTGGCAAAAGGTGAAAAAAGTAGCATGATTTTTTCACCCACTAAGGATTTATGCAAGCAGTATGTAGATAAAGACTGTCTTTATGTCCAGAAAGAGATTTCACCTGCAACCCCCACTATACAGAAGACTAGAAACACCATAAATACCTCTGTAGTAGCTAAGCAGAAGCATTGCAAAAAACACATCACAGCTGAAAATACGAAGAGCGGTTTGGTGTGTCTAACACAAGACCAACTACAACAGATTTTAATGACTGTGAACCAAGGAAATAAGTCTATTTCCGCGATTGAAAATGGAAAGGAGGAAACAA

**>Exon 4a**

ATGGGAAGTAAGAACAAGATTGCAAAATGTCCTATAAGAACAAAACAGACTGGATACATTCTAAAATCAACACAAAATACATGTATCAGGAGTGGAAAACTTTTGCAAAAGAAGAGAATGGGTTCAGAAACTTCACTGGCAAAAGGTGAAAAAAGTAGCATGATTTTTTCACCCACTAAGGATTTATGCAAGCAGTATGTAGATAAAGACTGTCTTTATGTCCAGAAAGAGATTTCACCTGCAACCCCCACTATACAGAAGACTAGAAACACCATAAATACCTCTGTAGTAGCTAAGCAGAAGCATTGCAAAAAACACATCACAGCTGAAAATACGAAGAGCGGTTTGGTGTGTCTAACACAAGACCAACTACAACAGATTTTAATGACTGTGAACCAAGGAAATAAGTCTATTTCCGCGATTGAAAATGGAAAGGAGGAAACAA

**>Exon 4a+c**

ATGGGAAGTAAGAACAAGATTGCAAAATGTCCTATAAGAACAAAACAGACTGGATACATTCTAAAATCAACACAAAATACATGTATCAGGAGTGGAAAACTTTTGCAAAAGAAGAGAATGGGTTCAGAAACTTCACTGGCAAAAGGTGAAAAAAGTAGCATGATTTTTTCACCCACTAAGGATTTATGCAAGCAGTATGTAGATAAAGACTGTCTTTATGTCCAGAAAGAGATTTCACCTGCAACCCCCACTATACAGAAGACTAGAAACACCATAAATACCTCTGTAGTAGCTAAGCAGAAGCATTGCAAAAAACACATCACAGCTGAAAATACGAAGAGCGGTTTGGTGTGTCTAACACAAGACCAACTACAACAGATTTTAATGACTGTGAACCAAGGAAATAAGTCTATTTCCGCGATTGAAAATGGAAAGGAGGAAACAA

**>Exon 5**

GTCAAGACAGTCTACATTTAAACAATACTTCCAATCAGCCAAAAGATGAGAACATAATGGGAGTATTCCAAAAAAATGAGGCTCTTTCATCTGTCCTGGATGAAAATAAATCCACTTTAAATAAAAATCAAGAGACATCTAAGCAGTATGAGCAGAAAATTGCCAT

**>Exon 6**

AGAGAATGTATGGAAACCAGCTGACATATTCAGTACTCTGGGCGAAAGAGAACGTGACAGAAGTTTGTTGGAAGCAAAAAAAGCCCAGTGGAAGAAAGAGCTTG

**>Exon 7**

ATGAACAGGTTGCTTTAAAGAAGAAAGAAAAAGAAGCTTCTGAAAAATGGAACAATCCTTGGAAAAAATTTGAAAGTGATAAAATAGTGTGGGAAAAATTTCAAACTCTTGGCCAGTCTAAG

**>Exon 8**

ACTAGTCTTAGCTCTTCCAACATTCTGTCACAGTCTCCCAGTCAGATAACAGTGGTCCAGGCTGATGACTACCCACTATGTAGAGCCAGTCAGATTTTAGAG

**>Exon 9**

GAAACAGTACCACTGGAGCGCCCTTTGAGTACTGTGAAACAAGAACAGCAGAGAAAATGGATTGAAGATTTGAACAAGCAAATAGAAGATGATCGGCAAAGAAAAATAGAGGAAAAAATTACATCTTCAAAG

**>Exon 10**

GGTGAGGAACATGACAGATGGGCAATGCATTTTGATTCATTAAAGAACTATCCTGCTTCTCAGTCTCAACTGTCCTCTCGGTCAATACACAACCAACCAGAGTACTTCTGTGTCTCTCCGGACACTCAGGAACTGTCTGATATCAGCAATGTTTATACACCAACGACTGGAAGCCAGGTTGAACCTTCAGAGGAGGAGCATATAGCAAAACCTGTTAGAGATATGGCTATGGCAAATAGTCAGAAAACAAA

**>Exon 10a**

TCTCAACTGTCCTCTCGGTCAATACACAACCAACCAGAGTACTTCTGTGTCTCTCCGGACACTCAGGAACTGTCTGATATCAGCAATGTTTATACACCAACGACTGGAAGCCAGGTTGAACCTTCAGAGGAGGAGCATATAGCAAAACCTGTTAGAGATATGGCTATGGCAAATAGTCAGAAAACAAA

**>Exon 11**

CTTTCTCCGTTCTATGACTGCTCTCCTGGATCCAGCTCAGATTGAAGAACGTGACAGGCGACGACAAAAACAGTTAGAACATCAG

**>Exon 12**

AAAGCCATCACTGCTCAGGTAGAAGAGAAACGCAGGAAGAAGCAGCTGGAAGAACAGCAAAGAAAGAAGGAAGAACAAGAAGAGGAGCGTCGCTTAGCGAGGGAACGAGAAGAGATGCAGAAACAGTATGAAGAAGACATACTTAAGCAAAAACAAAAGGAA

**>Exon 13**

GAAATCATGACTCTCAAGACAAATGAATTGTTCCAGACAATGCAGAGAGCACAGGAGCTAGCACAGAGACTGAAACAAGAACAGAGAATACGAGAATTGGCTCAGAAGGGACATGATACGTCTGGATTGATTAAAAATCTTGGTG

**>Exon 14**

GATATGGCTTGGATGATGTCAGTGGTAAAATGAATACATGTATTAATTCTACAACCTCTCCCAAAAAGGATACTGCTGTACAAACAG

**>Exon 15**

ATGACTTAAATACAGGAATGTTCACCATTGCAGAATCATGCTGTGGATCAATAATAGAGAGGGAAATTCTAAATTGCTCATCTCCTGAGATTCCTGCAGAATTTAATGACCAGTTTAAGAAAGACAAACAGGAACTAATCAATCAGGATAAAGCAGCCAACTTAGAAAAAGAAAACAGTTGGTACAATGATCAGTATGAGTTTGCAAGGACAGAGAAAAAACATATGAAGAAGTGTCCTAAAAGGCCTGATTGGAATATAAATAAGCCACTCAAAAGGTATATTCCGGCATCAGAAAAGTACCCTAAACAGCTTCAAAAGCAGAGAGAAGAAAAAAAAGTAAGAAGGCAGATGGAACTGCTTAATTTGGTAGAAAGAAATAATCCTGGACATCTCTCTCAAAATAGAGGGACTTCACCAGTTCTTCCTTCACCTCAAGAAGCAGAGGCAAGGTTCAGGTGGCATCTAATCAGAAAG

**>Exon 16**

GAGGAACCTCTGAAAAGTGATTCTTTCAGCAAGGAAAG

**>Exon 17**

GTCTCAGTCACCACTGGAACTGGTGAAAAACAGAACACAACAAACTCAGACACTAAAAAACAGAGAGAATCTGATCTTAGGAGACAGTCAGACAGAAACATCACCTGGAGCTTCTGAACCATCCCATTTTATCCCCTATGTCCGAACCAATGAGATTTATCATCTTGATCCAGATGCACCACTGTCCAGGCCTTTAACCCAGGATCTTCAGTACCAAAATCCACATG

**>Exon 18**

ACTGTGACCAAGAACAATGGCAGCTATTTGAATCTGATGTCAGGGACCCACTTCTCAATCCTAACTTGGTGAAAAACAGGGATCGACAGCAAGCAATCCTTAAGGGACTATCAGAACTGAGACAG

**>Exon 19**

GGCCTTCTCCAGAAGCAAAGGGAGTTGGAAACTAATCTCATGCCTTTAGCTGCAAATCAAGAAGAGAATTTTAATTCTTCGTTTTAAATGTAGAAAATCAAATCCTTCACATTTGTTGCTTTTAAATTATGTGTTCATTGCATAACTGTATTTTCCA

**2) CCDC66 transcripts, canine**

**>Transcript I**

TACACAGGTGGCTGGAGCGTCCTGTGGAGAGAGCGAGAGGTCAGGCCATGAACCTGGGAGATGGCTTAAAGCTTGAAACTGAATTGTTGGATGGAAAAACCAAGTTAATATTGTCTCCATATGAATGTAAATCAAAAAATTCTGTGAAGATGGGAAGTAAGAACAAGATTGCAAAATGTCCTATAAGAACAAAACAGACTGGATACATTCTAAAATCAACACAAAATACATGTATCAGGAGTGGAAAACTTTTGCAAAAGAAGAGAATGGGTTCAGAAACTTCACTGGCAAAAGGTGAAAAAAGTAGCATGATTTTTTCACCCACTAAGGATTTATGCAAGCAGTATGTAGATAAAGACTGTCTTTATGTCCAGAAAGAGATTTCACCTGCAACCCCCACTATACAGAAGACTAGAAACACCATAAATACCTCTGTAGTAGCTAAGCAGAAGCATTGCAAAAAACACATCACAGCTGAAAATACGAAGAGCGGTTTGGTGTGTCTAACACAAGACCAACTACAACAGATTTTAATGACTGTGAACCAAGGAAATAAGTCTATTTCCGCGATTGAAAATGGAAAGGAGGAAACAAGTCAAGACAGTCTACATTTAAACAATACTTCCAATCAGCCAAAAGATGAGAACATAATGGGAGTATTCCAAAAAAATGAGGCTCTTTCATCTGTCCTGGATGAAAATAAATCCACTTTAAATAAAAATCAAGAGACATCTAAGCAGTATGAGCAGAAAATTGCCATAGAGAATGTATGGAAACCAGCTGACATATTCAGTACTCTGGGCGAAAGAGAACGTGACAGAAGTTTGTTGGAAGCAAAAAAAGCCCAGTGGAAGAAAGAGCTTGATGAACAGGTTGCTTTAAAGAAGAAAGAAAAAGAAGCTTCTGAAAAATGGAACAATCCTTGGAAAAAATTTGAAAGTGATAAAATAGTGTGGGAAAAATTTCAAACTCTTGGCCAGTCTAAGACTAGTCTTAGCTCTTCCAACATTCTGTCACAGTCTCCCAGTCAGATAACAGTGGTCCAGGCTGATGACTACCCACTATGTAGAGCCAGTCAGATTTTAGAGGAAACAGTACCACTGGAGCGCCCTTTGAGTACTGTGAAACAAGAACAGCAGAGAAAATGGATTGAAGATTTGAACAAGCAAATAGAAGATGATCGGCAAAGAAAAATAGAGGAAAAAATTACATCTTCAAAGGGTGAGGAACATGACAGATGGGCAATGCATTTTGATTCATTAAAGAACTATCCTGCTTCTCAGTCTCAACTGTCCTCTCGGTCAATACACAACCAACCAGAGTACTTCTGTGTCTCTCCGGACACTCAGGAACTGTCTGATATCAGCAATGTTTATACACCAACGACTGGAAGCCAGGTTGAACCTTCAGAGGAGGAGCATATAGCAAAACCTGTTAGAGATATGGCTATGGCAAATAGTCAGAAAACAAACTTTCTCCGTTCTATGACTGCTCTCCTGGATCCAGCTCAGATTGAAGAACGTGACAGGCGACGACAAAAACAGTTAGAACATCAGAAAGCCATCACTGCTCAGGTAGAAGAGAAACGCAGGAAGAAGCAGCTGGAAGAACAGCAAAGAAAGAAGGAAGAACAAGAAGAGGAGCGTCGCTTAGCGAGGGAACGAGAAGAGATGCAGAAACAGTATGAAGAAGACATACTTAAGCAAAAACAAAAGGAAGAAATCATGACTCTCAAGACAAATGAATTGTTCCAGACAATGCAGAGAGCACAGGAGCTAGCACAGAGACTGAAACAAGAACAGAGAATACGAGAATTGGCTCAGAAGGGACATGATACGTCTGGATTGATTAAAAATCTTGGTGGATATGGCTTGGATGATGTCAGTGGTAAAATGAATACATGTATTAATTCTACAACCTCTCCCAAAAAGGATACTGCTGTACAAACAGATGACTTAAATACAGGAATGTTCACCATTGCAGAATCATGCTGTGGATCAATAATAGAGAGGGAAATTCTAAATTGCTCATCTCCTGAGATTCCTGCAGAATTTAATGACCAGTTTAAGAAAGACAAACAGGAACTAATCAATCAGGATAAAGCAGCCAACTTAGAAAAAGAAAACAGTTGGTACAATGATCAGTATGAGTTTGCAAGGACAGAGAAAAAACATATGAAGAAGTGTCCTAAAAGGCCTGATTGGAATATAAATAAGCCACTCAAAAGGTATATTCCGGCATCAGAAAAGTACCCTAAACAGCTTCAAAAGCAGAGAGAAGAAAAAAAAGTAAGAAGGCAGATGGAACTGCTTAATTTGGTAGAAAGAAATAATCCTGGACATCTCTCTCAAAATAGAGGGACTTCACCAGTTCTTCCTTCACCTCAAGAAGCAGAGGCAAGGTTCAGGTGGCATCTAATCAGAAAGGAGGAACCTCTGAAAAGTGATTCTTTCAGCAAGGAAAGGTCTCAGTCACCACTGGAACTGGTGAAAAACAGAACACAACAAACTCAGACACTAAAAAACAGAGAGAATCTGATCTTAGGAGACAGTCAGACAGAAACATCACCTGGAGCTTCTGAACCATCCCATTTTATCCCCTATGTCCGAACCAATGAGATTTATCATCTTGATCCAGATGCACCACTGTCCAGGCCTTTAACCCAGGATCTTCAGTACCAAAATCCACATGACTGTGACCAAGAACAATGGCAGCTATTTGAATCTGATGTCAGGGACCCACTTCTCAATCCTAACTTGGTGAAAAACAGGGATCGACAGCAAGCAATCCTTAAGGGACTATCAGAACTGAGACAGGGCCTTCTCCAGAAGCAAAGGGAGTTGGAAACTAATCTCATGCCTTTAGCTGCAAATCAAGAAGAGAATTTTAATTCTTCGTTTTAAATGTAGAAAATCAAATCCTTCACATTTGTTGCTTTTAAATTATGTGTTCATTGCATAACTGTATTTTCCA

**>Transcript II**

TACACAGGTGGCTGGAGCGTCCTGTGGAGAGAGCGAGAGGTCAGGCCATGAACCTGGGAGATGGCTTAAAGCTTGAAACTGAATTGTTGGATGGAAAAACCAAGTTAATATTGTCTCCATATGATGGGAAGTAAGAACAAGATTGCAAAATGTCCTATAAGAACAAAACAGACTGGATACATTCTAAAATCAACACAAAATACATGTATCAGGAGTGGAAAACTTTTGCAAAAGAAGAGAATGGGTTCAGAAACTTCACTGGCAAAAGGTGAAAAAAGTAGCATGATTTTTTCACCCACTAAGGATTTATGCAAGCAGTATGTAGATAAAGACTGTCTTTATGTCCAGAAAGAGATTTCACCTGCAACCCCCACTATACAGAAGACTAGAAACACCATAAATACCTCTGTAGTAGCTAAGCAGAAGCATTGCAAAAAACACATCACAGCTGAAAATACGAAGAGCGGTTTGGTGTGTCTAACACAAGACCAACTACAACAGATTTTAATGACTGTGAACCAAGGAAATAAGTCTATTTCCGCGATTGAAAATGGAAAGGAGGAAACAAGTCAAGACAGTCTACATTTAAACAATACTTCCAATCAGCCAAAAGATGAGAACATAATGGGAGTATTCCAAAAAAATGAGGCTCTTTCATCTGTCCTGGATGAAAATAAATCCACTTTAAATAAAAATCAAGAGACATCTAAGCAGTATGAGCAGAAAATTGCCATAGAGAATGTATGGAAACCAGCTGACATATTCAGTACTCTGGGCGAAAGAGAACGTGACAGAAGTTTGTTGGAAGCAAAAAAAGCCCAGTGGAAGAAAGAGCTTGATGAACAGGTTGCTTTAAAGAAGAAAGAAAAAGAAGCTTCTGAAAAATGGAACAATCCTTGGAAAAAATTTGAAAGTGATAAAATAGTGTGGGAAAAATTTCAAACTCTTGGCCAGTCTAAGACTAGTCTTAGCTCTTCCAACATTCTGTCACAGTCTCCCAGTCAGATAACAGTGGTCCAGGCTGATGACTACCCACTATGTAGAGCCAGTCAGATTTTAGAGGAAACAGTACCACTGGAGCGCCCTTTGAGTACTGTGAAACAAGAACAGCAGAGAAAATGGATTGAAGATTTGAACAAGCAAATAGAAGATGATCGGCAAAGAAAAATAGAGGAAAAAATTACATCTTCAAAGGGTGAGGAACATGACAGATGGGCAATGCATTTTGATTCATTAAAGAACTATCCTGCTTCTCAGTCTCAACTGTCCTCTCGGTCAATACACAACCAACCAGAGTACTTCTGTGTCTCTCCGGACACTCAGGAACTGTCTGATATCAGCAATGTTTATACACCAACGACTGGAAGCCAGGTTGAACCTTCAGAGGAGGAGCATATAGCAAAACCTGTTAGAGATATGGCTATGGCAAATAGTCAGAAAACAAACTTTCTCCGTTCTATGACTGCTCTCCTGGATCCAGCTCAGATTGAAGAACGTGACAGGCGACGACAAAAACAGTTAGAACATCAGAAAGCCATCACTGCTCAGGTAGAAGAGAAACGCAGGAAGAAGCAGCTGGAAGAACAGCAAAGAAAGAAGGAAGAACAAGAAGAGGAGCGTCGCTTAGCGAGGGAACGAGAAGAGATGCAGAAACAGTATGAAGAAGACATACTTAAGCAAAAACAAAAGGAAGAAATCATGACTCTCAAGACAAATGAATTGTTCCAGACAATGCAGAGAGCACAGGAGCTAGCACAGAGACTGAAACAAGAACAGAGAATACGAGAATTGGCTCAGAAGGGACATGATACGTCTGGATTGATTAAAAATCTTGGTGGATATGGCTTGGATGATGTCAGTGGTAAAATGAATACATGTATTAATTCTACAACCTCTCCCAAAAAGGATACTGCTGTACAAACAGATGACTTAAATACAGGAATGTTCACCATTGCAGAATCATGCTGTGGATCAATAATAGAGAGGGAAATTCTAAATTGCTCATCTCCTGAGATTCCTGCAGAATTTAATGACCAGTTTAAGAAAGACAAACAGGAACTAATCAATCAGGATAAAGCAGCCAACTTAGAAAAAGAAAACAGTTGGTACAATGATCAGTATGAGTTTGCAAGGACAGAGAAAAAACATATGAAGAAGTGTCCTAAAAGGCCTGATTGGAATATAAATAAGCCACTCAAAAGGTATATTCCGGCATCAGAAAAGTACCCTAAACAGCTTCAAAAGCAGAGAGAAGAAAAAAAAGTAAGAAGGCAGATGGAACTGCTTAATTTGGTAGAAAGAAATAATCCTGGACATCTCTCTCAAAATAGAGGGACTTCACCAGTTCTTCCTTCACCTCAAGAAGCAGAGGCAAGGTTCAGGTGGCATCTAATCAGAAAGGAGGAACCTCTGAAAAGTGATTCTTTCAGCAAGGAAAGGTCTCAGTCACCACTGGAACTGGTGAAAAACAGAACACAACAAACTCAGACACTAAAAAACAGAGAGAATCTGATCTTAGGAGACAGTCAGACAGAAACATCACCTGGAGCTTCTGAACCATCCCATTTTATCCCCTATGTCCGAACCAATGAGATTTATCATCTTGATCCAGATGCACCACTGTCCAGGCCTTTAACCCAGGATCTTCAGTACCAAAATCCACATGACTGTGACCAAGAACAATGGCAGCTATTTGAATCTGATGTCAGGGACCCACTTCTCAATCCTAACTTGGTGAAAAACAGGGATCGACAGCAAGCAATCCTTAAGGGACTATCAGAACTGAGACAGGGCCTTCTCCAGAAGCAAAGGGAGTTGGAAACTAATCTCATGCCTTTAGCTGCAAATCAAGAAGAGAATTTTAATTCTTCGTTTTAAATGTAGAAAATCAAATCCTTCACATTTGTTGCTTTTAAATTATGTGTTCATTGCATAACTGTATTTTCCA

**>Transcript III**

TACACAGGTGGCTGGAGCGTCCTGTGGAGAGAGCGAGAGGTCAGGCCATGAACCTGGGAGATGGCTTAAAGCTTGAAACTGAATTGTTGGATGGAAAAACCAAGTTAATATTGTCTCCATATGAATGTAAATCAAAAAATTCTGTGAAGATGGGAAGTAAGAACAAGATTGCAAAATGTCCTATAAGAACAAAACAGACTGGATACATTCTAAAATCAACACAAAATACATGTATCAGGAGTGGAAAACTTTTGCAAAAGAAGAGAATGGGTTCAGAAACTTCACTGGCAAAAGAAAGAGATTTCACCTGCAACCCCCACTATACAGAAGACTAGAAACACCATAAATACCTCTGTAGTAGCTAAGCAGAAGCATTGCAAAAAACACATCACAGCTGAAAATACGAAGAGCGGTTTGGTGTGTCTAACACAAGACCAACTACAACAGATTTTAATGACTGTGAACCAAGGAAATAAGTCTATTTCCGCGATTGAAAATGGAAAGGAGGAAACAAGTCAAGACAGTCTACATTTAAACAATACTTCCAATCAGCCAAAAGATGAGAACATAATGGGAGTATTCCAAAAAAATGAGGCTCTTTCATCTGTCCTGGATGAAAATAAATCCACTTTAAATAAAAATCAAGAGACATCTAAGCAGTATGAGCAGAAAATTGCCATAGAGAATGTATGGAAACCAGCTGACATATTCAGTACTCTGGGCGAAAGAGAACGTGACAGAAGTTTGTTGGAAGCAAAAAAAGCCCAGTGGAAGAAAGAGCTTGATGAACAGGTTGCTTTAAAGAAGAAAGAAAAAGAAGCTTCTGAAAAATGGAACAATCCTTGGAAAAAATTTGAAAGTGATAAAATAGTGTGGGAAAAATTTCAAACTCTTGGCCAGTCTAAGACTAGTCTTAGCTCTTCCAACATTCTGTCACAGTCTCCCAGTCAGATAACAGTGGTCCAGGCTGATGACTACCCACTATGTAGAGCCAGTCAGATTTTAGAGGAAACAGTACCACTGGAGCGCCCTTTGAGTACTGTGAAACAAGAACAGCAGAGAAAATGGATTGAAGATTTGAACAAGCAAATAGAAGATGATCGGCAAAGAAAAATAGAGGAAAAAATTACATCTTCAAAGGGTGAGGAACATGACAGATGGGCAATGCATTTTGATTCATTAAAGAACTATCCTGCTTCTCAGTCTCAACTGTCCTCTCGGTCAATACACAACCAACCAGAGTACTTCTGTGTCTCTCCGGACACTCAGGAACTGTCTGATATCAGCAATGTTTATACACCAACGACTGGAAGCCAGGTTGAACCTTCAGAGGAGGAGCATATAGCAAAACCTGTTAGAGATATGGCTATGGCAAATAGTCAGAAAACAAACTTTCTCCGTTCTATGACTGCTCTCCTGGATCCAGCTCAGATTGAAGAACGTGACAGGCGACGACAAAAACAGTTAGAACATCAGAAAGCCATCACTGCTCAGGTAGAAGAGAAACGCAGGAAGAAGCAGCTGGAAGAACAGCAAAGAAAGAAGGAAGAACAAGAAGAGGAGCGTCGCTTAGCGAGGGAACGAGAAGAGATGCAGAAACAGTATGAAGAAGACATACTTAAGCAAAAACAAAAGGAAGAAATCATGACTCTCAAGACAAATGAATTGTTCCAGACAATGCAGAGAGCACAGGAGCTAGCACAGAGACTGAAACAAGAACAGAGAATACGAGAATTGGCTCAGAAGGGACATGATACGTCTGGATTGATTAAAAATCTTGGTGGATATGGCTTGGATGATGTCAGTGGTAAAATGAATACATGTATTAATTCTACAACCTCTCCCAAAAAGGATACTGCTGTACAAACAGATGACTTAAATACAGGAATGTTCACCATTGCAGAATCATGCTGTGGATCAATAATAGAGAGGGAAATTCTAAATTGCTCATCTCCTGAGATTCCTGCAGAATTTAATGACCAGTTTAAGAAAGACAAACAGGAACTAATCAATCAGGATAAAGCAGCCAACTTAGAAAAAGAAAACAGTTGGTACAATGATCAGTATGAGTTTGCAAGGACAGAGAAAAAACATATGAAGAAGTGTCCTAAAAGGCCTGATTGGAATATAAATAAGCCACTCAAAAGGTATATTCCGGCATCAGAAAAGTACCCTAAACAGCTTCAAAAGCAGAGAGAAGAAAAAAAAGTAAGAAGGCAGATGGAACTGCTTAATTTGGTAGAAAGAAATAATCCTGGACATCTCTCTCAAAATAGAGGGACTTCACCAGTTCTTCCTTCACCTCAAGAAGCAGAGGCAAGGTTCAGGTGGCATCTAATCAGAAAGGAGGAACCTCTGAAAAGTGATTCTTTCAGCAAGGAAAGGTCTCAGTCACCACTGGAACTGGTGAAAAACAGAACACAACAAACTCAGACACTAAAAAACAGAGAGAATCTGATCTTAGGAGACAGTCAGACAGAAACATCACCTGGAGCTTCTGAACCATCCCATTTTATCCCCTATGTCCGAACCAATGAGATTTATCATCTTGATCCAGATGCACCACTGTCCAGGCCTTTAACCCAGGATCTTCAGTACCAAAATCCACATGACTGTGACCAAGAACAATGGCAGCTATTTGAATCTGATGTCAGGGACCCACTTCTCAATCCTAACTTGGTGAAAAACAGGGATCGACAGCAAGCAATCCTTAAGGGACTATCAGAACTGAGACAGGGCCTTCTCCAGAAGCAAAGGGAGTTGGAAACTAATCTCATGCCTTTAGCTGCAAATCAAGAAGAGAATTTTAATTCTTCGTTTTAAATGTAGAAAATCAAATCCTTCACATTTGTTGCTTTTAAATTATGTGTTCATTGCATAACTGTATTTTCCA

**>Transcript IV**

TACACAGGTGGCTGGAGCGTCCTGTGGAGAGAGCGAGAGGTCAGGCCATGAACCTGGGAGATGGCTTAAAGCTTGAAACTGAATTGTTGGATGGAAAAACCAAGTTAATATTGTCTCCATATGATGGGAAGTAAGAACAAGATTGCAAAATGTCCTATAAGAACAAAACAGACTGGATACATTCTAAAATCAACACAAAATACATGTATCAGGAGTGGAAAACTTTTGCAAAAGAAGAGAATGGGTTCAGAAACTTCACTGGCAAAAGAAAGAGATTTCACCTGCAACCCCCACTATACAGAAGACTAGAAACACCATAAATACCTCTGTAGTAGCTAAGCAGAAGCATTGCAAAAAACACATCACAGCTGAAAATACGAAGAGCGGTTTGGTGTGTCTAACACAAGACCAACTACAACAGATTTTAATGACTGTGAACCAAGGAAATAAGTCTATTTCCGCGATTGAAAATGGAAAGGAGGAAACAAGTCAAGACAGTCTACATTTAAACAATACTTCCAATCAGCCAAAAGATGAGAACATAATGGGAGTATTCCAAAAAAATGAGGCTCTTTCATCTGTCCTGGATGAAAATAAATCCACTTTAAATAAAAATCAAGAGACATCTAAGCAGTATGAGCAGAAAATTGCCATAGAGAATGTATGGAAACCAGCTGACATATTCAGTACTCTGGGCGAAAGAGAACGTGACAGAAGTTTGTTGGAAGCAAAAAAAGCCCAGTGGAAGAAAGAGCTTGATGAACAGGTTGCTTTAAAGAAGAAAGAAAAAGAAGCTTCTGAAAAATGGAACAATCCTTGGAAAAAATTTGAAAGTGATAAAATAGTGTGGGAAAAATTTCAAACTCTTGGCCAGTCTAAGACTAGTCTTAGCTCTTCCAACATTCTGTCACAGTCTCCCAGTCAGATAACAGTGGTCCAGGCTGATGACTACCCACTATGTAGAGCCAGTCAGATTTTAGAGGAAACAGTACCACTGGAGCGCCCTTTGAGTACTGTGAAACAAGAACAGCAGAGAAAATGGATTGAAGATTTGAACAAGCAAATAGAAGATGATCGGCAAAGAAAAATAGAGGAAAAAATTACATCTTCAAAGGGTGAGGAACATGACAGATGGGCAATGCATTTTGATTCATTAAAGAACTATCCTGCTTCTCAGTCTCAACTGTCCTCTCGGTCAATACACAACCAACCAGAGTACTTCTGTGTCTCTCCGGACACTCAGGAACTGTCTGATATCAGCAATGTTTATACACCAACGACTGGAAGCCAGGTTGAACCTTCAGAGGAGGAGCATATAGCAAAACCTGTTAGAGATATGGCTATGGCAAATAGTCAGAAAACAAACTTTCTCCGTTCTATGACTGCTCTCCTGGATCCAGCTCAGATTGAAGAACGTGACAGGCGACGACAAAAACAGTTAGAACATCAGAAAGCCATCACTGCTCAGGTAGAAGAGAAACGCAGGAAGAAGCAGCTGGAAGAACAGCAAAGAAAGAAGGAAGAACAAGAAGAGGAGCGTCGCTTAGCGAGGGAACGAGAAGAGATGCAGAAACAGTATGAAGAAGACATACTTAAGCAAAAACAAAAGGAAGAAATCATGACTCTCAAGACAAATGAATTGTTCCAGACAATGCAGAGAGCACAGGAGCTAGCACAGAGACTGAAACAAGAACAGAGAATACGAGAATTGGCTCAGAAGGGACATGATACGTCTGGATTGATTAAAAATCTTGGTGGATATGGCTTGGATGATGTCAGTGGTAAAATGAATACATGTATTAATTCTACAACCTCTCCCAAAAAGGATACTGCTGTACAAACAGATGACTTAAATACAGGAATGTTCACCATTGCAGAATCATGCTGTGGATCAATAATAGAGAGGGAAATTCTAAATTGCTCATCTCCTGAGATTCCTGCAGAATTTAATGACCAGTTTAAGAAAGACAAACAGGAACTAATCAATCAGGATAAAGCAGCCAACTTAGAAAAAGAAAACAGTTGGTACAATGATCAGTATGAGTTTGCAAGGACAGAGAAAAAACATATGAAGAAGTGTCCTAAAAGGCCTGATTGGAATATAAATAAGCCACTCAAAAGGTATATTCCGGCATCAGAAAAGTACCCTAAACAGCTTCAAAAGCAGAGAGAAGAAAAAAAAGTAAGAAGGCAGATGGAACTGCTTAATTTGGTAGAAAGAAATAATCCTGGACATCTCTCTCAAAATAGAGGGACTTCACCAGTTCTTCCTTCACCTCAAGAAGCAGAGGCAAGGTTCAGGTGGCATCTAATCAGAAAGGAGGAACCTCTGAAAAGTGATTCTTTCAGCAAGGAAAGGTCTCAGTCACCACTGGAACTGGTGAAAAACAGAACACAACAAACTCAGACACTAAAAAACAGAGAGAATCTGATCTTAGGAGACAGTCAGACAGAAACATCACCTGGAGCTTCTGAACCATCCCATTTTATCCCCTATGTCCGAACCAATGAGATTTATCATCTTGATCCAGATGCACCACTGTCCAGGCCTTTAACCCAGGATCTTCAGTACCAAAATCCACATGACTGTGACCAAGAACAATGGCAGCTATTTGAATCTGATGTCAGGGACCCACTTCTCAATCCTAACTTGGTGAAAAACAGGGATCGACAGCAAGCAATCCTTAAGGGACTATCAGAACTGAGACAGGGCCTTCTCCAGAAGCAAAGGGAGTTGGAAACTAATCTCATGCCTTTAGCTGCAAATCAAGAAGAGAATTTTAATTCTTCGTTTTAAATGTAGAAAATCAAATCCTTCACATTTGTTGCTTTTAAATTATGTGTTCATTGCATAACTGTATTTTCCA

**>Transcript V**

TACACAGGTGGCTGGAGCGTCCTGTGGAGAGAGCGAGAGGTCAGGCCATGAACCTGGGAGATGGCTTAAAGCTTGAAACTGAATTGTTGGATGGAAAAACCAAGTTAATATTGTCTCCATATGAATGTAAATCAAAAAATTCTGTGAAGATGGGAAGTAAGAACAAGATTGCAAAATGTCCTATAAGAACAAAACAGACTGGATACATTCTAAAATCAACACAAAATACATGTATCAGGAGTGGAAAACTTTTGCAAAAGAAGAGAATGGGTTCAGAAACTTCACTGGCAAAAGGTCAAGACAGTCTACATTTAAACAATACTTCCAATCAGCCAAAAGATGAGAACATAATGGGAGTATTCCAAAAAAATGAGGCTCTTTCATCTGTCCTGGATGAAAATAAATCCACTTTAAATAAAAATCAAGAGACATCTAAGCAGTATGAGCAGAAAATTGCCATAGAGAATGTATGGAAACCAGCTGACATATTCAGTACTCTGGGCGAAAGAGAACGTGACAGAAGTTTGTTGGAAGCAAAAAAAGCCCAGTGGAAGAAAGAGCTTGATGAACAGGTTGCTTTAAAGAAGAAAGAAAAAGAAGCTTCTGAAAAATGGAACAATCCTTGGAAAAAATTTGAAAGTGATAAAATAGTGTGGGAAAAATTTCAAACTCTTGGCCAGTCTAAGACTAGTCTTAGCTCTTCCAACATTCTGTCACAGTCTCCCAGTCAGATAACAGTGGTCCAGGCTGATGACTACCCACTATGTAGAGCCAGTCAGATTTTAGAGGAAACAGTACCACTGGAGCGCCCTTTGAGTACTGTGAAACAAGAACAGCAGAGAAAATGGATTGAAGATTTGAACAAGCAAATAGAAGATGATCGGCAAAGAAAAATAGAGGAAAAAATTACATCTTCAAAGGGTGAGGAACATGACAGATGGGCAATGCATTTTGATTCATTAAAGAACTATCCTGCTTCTCAGTCTCAACTGTCCTCTCGGTCAATACACAACCAACCAGAGTACTTCTGTGTCTCTCCGGACACTCAGGAACTGTCTGATATCAGCAATGTTTATACACCAACGACTGGAAGCCAGGTTGAACCTTCAGAGGAGGAGCATATAGCAAAACCTGTTAGAGATATGGCTATGGCAAATAGTCAGAAAACAAACTTTCTCCGTTCTATGACTGCTCTCCTGGATCCAGCTCAGATTGAAGAACGTGACAGGCGACGACAAAAACAGTTAGAACATCAGAAAGCCATCACTGCTCAGGTAGAAGAGAAACGCAGGAAGAAGCAGCTGGAAGAACAGCAAAGAAAGAAGGAAGAACAAGAAGAGGAGCGTCGCTTAGCGAGGGAACGAGAAGAGATGCAGAAACAGTATGAAGAAGACATACTTAAGCAAAAACAAAAGGAAGAAATCATGACTCTCAAGACAAATGAATTGTTCCAGACAATGCAGAGAGCACAGGAGCTAGCACAGAGACTGAAACAAGAACAGAGAATACGAGAATTGGCTCAGAAGGGACATGATACGTCTGGATTGATTAAAAATCTTGGTGGATATGGCTTGGATGATGTCAGTGGTAAAATGAATACATGTATTAATTCTACAACCTCTCCCAAAAAGGATACTGCTGTACAAACAGATGACTTAAATACAGGAATGTTCACCATTGCAGAATCATGCTGTGGATCAATAATAGAGAGGGAAATTCTAAATTGCTCATCTCCTGAGATTCCTGCAGAATTTAATGACCAGTTTAAGAAAGACAAACAGGAACTAATCAATCAGGATAAAGCAGCCAACTTAGAAAAAGAAAACAGTTGGTACAATGATCAGTATGAGTTTGCAAGGACAGAGAAAAAACATATGAAGAAGTGTCCTAAAAGGCCTGATTGGAATATAAATAAGCCACTCAAAAGGTATATTCCGGCATCAGAAAAGTACCCTAAACAGCTTCAAAAGCAGAGAGAAGAAAAAAAAGTAAGAAGGCAGATGGAACTGCTTAATTTGGTAGAAAGAAATAATCCTGGACATCTCTCTCAAAATAGAGGGACTTCACCAGTTCTTCCTTCACCTCAAGAAGCAGAGGCAAGGTTCAGGTGGCATCTAATCAGAAAGGAGGAACCTCTGAAAAGTGATTCTTTCAGCAAGGAAAGGTCTCAGTCACCACTGGAACTGGTGAAAAACAGAACACAACAAACTCAGACACTAAAAAACAGAGAGAATCTGATCTTAGGAGACAGTCAGACAGAAACATCACCTGGAGCTTCTGAACCATCCCATTTTATCCCCTATGTCCGAACCAATGAGATTTATCATCTTGATCCAGATGCACCACTGTCCAGGCCTTTAACCCAGGATCTTCAGTACCAAAATCCACATGACTGTGACCAAGAACAATGGCAGCTATTTGAATCTGATGTCAGGGACCCACTTCTCAATCCTAACTTGGTGAAAAACAGGGATCGACAGCAAGCAATCCTTAAGGGACTATCAGAACTGAGACAGGGCCTTCTCCAGAAGCAAAGGGAGTTGGAAACTAATCTCATGCCTTTAGCTGCAAATCAAGAAGAGAATTTTAATTCTTCGTTTTAAATGTAGAAAATCAAATCCTTCACATTTGTTGCTTTTAAATTATGTGTTCATTGCATAACTGTATTTTCCA

**>Transcript VI**

TACACAGGTGGCTGGAGCGTCCTGTGGAGAGAGCGAGAGGTCAGGCCATGAACCTGGGAGATGGCTTAAAGCTTGAAACTGAATTGTTGGATGGAAAAACCAAGTTAATATTGTCTCCATATGATGGGAAGTAAGAACAAGATTGCAAAATGTCCTATAAGAACAAAACAGACTGGATACATTCTAAAATCAACACAAAATACATGTATCAGGAGTGGAAAACTTTTGCAAAAGAAGAGAATGGGTTCAGAAACTTCACTGGCAAAAGGTCAAGACAGTCTACATTTAAACAATACTTCCAATCAGCCAAAAGATGAGAACATAATGGGAGTATTCCAAAAAAATGAGGCTCTTTCATCTGTCCTGGATGAAAATAAATCCACTTTAAATAAAAATCAAGAGACATCTAAGCAGTATGAGCAGAAAATTGCCATAGAGAATGTATGGAAACCAGCTGACATATTCAGTACTCTGGGCGAAAGAGAACGTGACAGAAGTTTGTTGGAAGCAAAAAAAGCCCAGTGGAAGAAAGAGCTTGATGAACAGGTTGCTTTAAAGAAGAAAGAAAAAGAAGCTTCTGAAAAATGGAACAATCCTTGGAAAAAATTTGAAAGTGATAAAATAGTGTGGGAAAAATTTCAAACTCTTGGCCAGTCTAAGACTAGTCTTAGCTCTTCCAACATTCTGTCACAGTCTCCCAGTCAGATAACAGTGGTCCAGGCTGATGACTACCCACTATGTAGAGCCAGTCAGATTTTAGAGGAAACAGTACCACTGGAGCGCCCTTTGAGTACTGTGAAACAAGAACAGCAGAGAAAATGGATTGAAGATTTGAACAAGCAAATAGAAGATGATCGGCAAAGAAAAATAGAGGAAAAAATTACATCTTCAAAGGGTGAGGAACATGACAGATGGGCAATGCATTTTGATTCATTAAAGAACTATCCTGCTTCTCAGTCTCAACTGTCCTCTCGGTCAATACACAACCAACCAGAGTACTTCTGTGTCTCTCCGGACACTCAGGAACTGTCTGATATCAGCAATGTTTATACACCAACGACTGGAAGCCAGGTTGAACCTTCAGAGGAGGAGCATATAGCAAAACCTGTTAGAGATATGGCTATGGCAAATAGTCAGAAAACAAACTTTCTCCGTTCTATGACTGCTCTCCTGGATCCAGCTCAGATTGAAGAACGTGACAGGCGACGACAAAAACAGTTAGAACATCAGAAAGCCATCACTGCTCAGGTAGAAGAGAAACGCAGGAAGAAGCAGCTGGAAGAACAGCAAAGAAAGAAGGAAGAACAAGAAGAGGAGCGTCGCTTAGCGAGGGAACGAGAAGAGATGCAGAAACAGTATGAAGAAGACATACTTAAGCAAAAACAAAAGGAAGAAATCATGACTCTCAAGACAAATGAATTGTTCCAGACAATGCAGAGAGCACAGGAGCTAGCACAGAGACTGAAACAAGAACAGAGAATACGAGAATTGGCTCAGAAGGGACATGATACGTCTGGATTGATTAAAAATCTTGGTGGATATGGCTTGGATGATGTCAGTGGTAAAATGAATACATGTATTAATTCTACAACCTCTCCCAAAAAGGATACTGCTGTACAAACAGATGACTTAAATACAGGAATGTTCACCATTGCAGAATCATGCTGTGGATCAATAATAGAGAGGGAAATTCTAAATTGCTCATCTCCTGAGATTCCTGCAGAATTTAATGACCAGTTTAAGAAAGACAAACAGGAACTAATCAATCAGGATAAAGCAGCCAACTTAGAAAAAGAAAACAGTTGGTACAATGATCAGTATGAGTTTGCAAGGACAGAGAAAAAACATATGAAGAAGTGTCCTAAAAGGCCTGATTGGAATATAAATAAGCCACTCAAAAGGTATATTCCGGCATCAGAAAAGTACCCTAAACAGCTTCAAAAGCAGAGAGAAGAAAAAAAAGTAAGAAGGCAGATGGAACTGCTTAATTTGGTAGAAAGAAATAATCCTGGACATCTCTCTCAAAATAGAGGGACTTCACCAGTTCTTCCTTCACCTCAAGAAGCAGAGGCAAGGTTCAGGTGGCATCTAATCAGAAAGGAGGAACCTCTGAAAAGTGATTCTTTCAGCAAGGAAAGGTCTCAGTCACCACTGGAACTGGTGAAAAACAGAACACAACAAACTCAGACACTAAAAAACAGAGAGAATCTGATCTTAGGAGACAGTCAGACAGAAACATCACCTGGAGCTTCTGAACCATCCCATTTTATCCCCTATGTCCGAACCAATGAGATTTATCATCTTGATCCAGATGCACCACTGTCCAGGCCTTTAACCCAGGATCTTCAGTACCAAAATCCACATGACTGTGACCAAGAACAATGGCAGCTATTTGAATCTGATGTCAGGGACCCACTTCTCAATCCTAACTTGGTGAAAAACAGGGATCGACAGCAAGCAATCCTTAAGGGACTATCAGAACTGAGACAGGGCCTTCTCCAGAAGCAAAGGGAGTTGGAAACTAATCTCATGCCTTTAGCTGCAAATCAAGAAGAGAATTTTAATTCTTCGTTTTAAATGTAGAAAATCAAATCCTTCACATTTGTTGCTTTTAAATTATGTGTTCATTGCATAACTGTATTTTCCA

**>Transcript VII**

TACACAGGTGGCTGGAGCGTCCTGTGGAGAGAGCGAGAGGTCAGGCCATGAACCTGGGAGATGGCTTAAAGCTTGAAACTGAATTGTTGGATGGAAAAACCAAGTTAATATTGTCTCCATATGAATGTAAATCAAAAAATTCTGTGAAGATGGGAAGTAAGAACAAGATTGCAAAATGTCCTATAAGAACAAAACAGACTGGATACATTCTAAAATCAACACAAAATACATGTATCAGGAGTGGAAAACTTTTGCAAAAGAAGAGAATGGGTTCAGAAACTTCACTGGCAAAAGGTGAAAAAAGTAGCATGATTTTTTCACCCACTAAGGATTTATGCAAGCAGTATGTAGATAAAGACTGTCTTTATGTCCAGAAAGAGATTTCACCTGCAACCCCCACTATACAGAAGACTAGAAACACCATAAATACCTCTGTAGTAGCTAAGCAGAAGCATTGCAAAAAACACATCACAGCTGAAAATACGAAGAGCGGTTTGGTGTGTCTAACACAAGACCAACTACAACAGATTTTAATGACTGTGAACCAAGGAAATAAGTCTATTTCCGCGATTGAAAATGGAAAGGAGGAAACAAGTCAAGACAGTCTACATTTAAACAATACTTCCAATCAGCCAAAAGATGAGAACATAATGGGAGTATTCCAAAAAAATGAGGCTCTTTCATCTGTCCTGGATGAAAATAAATCCACTTTAAATAAAAATCAAGAGACATCTAAGCAGTATGAGCAGAAAATTGCCATAGAGAATGTATGGAAACCAGCTGACATATTCAGTACTCTGGGCGAAAGAGAACGTGACAGAAGTTTGTTGGAAGCAAAAAAAGCCCAGTGGAAGAAAGAGCTTGATGAACAGGTTGCTTTAAAGAAGAAAGAAAAAGAAGCTTCTGAAAAATGGAACAATCCTTGGAAAAAATTTGAAAGTGATAAAATAGTGTGGGAAAAATTTCAAACTCTTGGCCAGTCTAAGACTAGTCTTAGCTCTTCCAACATTCTGTCACAGTCTCCCAGTCAGATAACAGTGGTCCAGGCTGATGACTACCCACTATGTAGAGCCAGTCAGATTTTAGAGGAAACAGTACCACTGGAGCGCCCTTTGAGTACTGTGAAACAAGAACAGCAGAGAAAATGGATTGAAGATTTGAACAAGCAAATAGAAGATGATCGGCAAAGAAAAATAGAGGAAAAAATTACATCTTCAAAGTCTCAACTGTCCTCTCGGTCAATACACAACCAACCAGAGTACTTCTGTGTCTCTCCGGACACTCAGGAACTGTCTGATATCAGCAATGTTTATACACCAACGACTGGAAGCCAGGTTGAACCTTCAGAGGAGGAGCATATAGCAAAACCTGTTAGAGATATGGCTATGGCAAATAGTCAGAAAACAAACTTTCTCCGTTCTATGACTGCTCTCCTGGATCCAGCTCAGATTGAAGAACGTGACAGGCGACGACAAAAACAGTTAGAACATCAGAAAGCCATCACTGCTCAGGTAGAAGAGAAACGCAGGAAGAAGCAGCTGGAAGAACAGCAAAGAAAGAAGGAAGAACAAGAAGAGGAGCGTCGCTTAGCGAGGGAACGAGAAGAGATGCAGAAACAGTATGAAGAAGACATACTTAAGCAAAAACAAAAGGAAGAAATCATGACTCTCAAGACAAATGAATTGTTCCAGACAATGCAGAGAGCACAGGAGCTAGCACAGAGACTGAAACAAGAACAGAGAATACGAGAATTGGCTCAGAAGGGACATGATACGTCTGGATTGATTAAAAATCTTGGTGGATATGGCTTGGATGATGTCAGTGGTAAAATGAATACATGTATTAATTCTACAACCTCTCCCAAAAAGGATACTGCTGTACAAACAGATGACTTAAATACAGGAATGTTCACCATTGCAGAATCATGCTGTGGATCAATAATAGAGAGGGAAATTCTAAATTGCTCATCTCCTGAGATTCCTGCAGAATTTAATGACCAGTTTAAGAAAGACAAACAGGAACTAATCAATCAGGATAAAGCAGCCAACTTAGAAAAAGAAAACAGTTGGTACAATGATCAGTATGAGTTTGCAAGGACAGAGAAAAAACATATGAAGAAGTGTCCTAAAAGGCCTGATTGGAATATAAATAAGCCACTCAAAAGGTATATTCCGGCATCAGAAAAGTACCCTAAACAGCTTCAAAAGCAGAGAGAAGAAAAAAAAGTAAGAAGGCAGATGGAACTGCTTAATTTGGTAGAAAGAAATAATCCTGGACATCTCTCTCAAAATAGAGGGACTTCACCAGTTCTTCCTTCACCTCAAGAAGCAGAGGCAAGGTTCAGGTGGCATCTAATCAGAAAGGAGGAACCTCTGAAAAGTGATTCTTTCAGCAAGGAAAGGTCTCAGTCACCACTGGAACTGGTGAAAAACAGAACACAACAAACTCAGACACTAAAAAACAGAGAGAATCTGATCTTAGGAGACAGTCAGACAGAAACATCACCTGGAGCTTCTGAACCATCCCATTTTATCCCCTATGTCCGAACCAATGAGATTTATCATCTTGATCCAGATGCACCACTGTCCAGGCCTTTAACCCAGGATCTTCAGTACCAAAATCCACATGACTGTGACCAAGAACAATGGCAGCTATTTGAATCTGATGTCAGGGACCCACTTCTCAATCCTAACTTGGTGAAAAACAGGGATCGACAGCAAGCAATCCTTAAGGGACTATCAGAACTGAGACAGGGCCTTCTCCAGAAGCAAAGGGAGTTGGAAACTAATCTCATGCCTTTAGCTGCAAATCAAGAAGAGAATTTTAATTCTTCGTTTTAAATGTAGAAAATCAAATCCTTCACATTTGTTGCTTTTAAATTATGTGTTCATTGCATAACTGTATTTTCCA

**>Transcript VIII**

TACACAGGTGGCTGGAGCGTCCTGTGGAGAGAGCGAGAGGTCAGGCCATGAACCTGGGAGATGGCTTAAAGCTTGAAACTGAATTGTTGGATGGAAAAACCAAGTTAATATTGTCTCCATATGATGGGAAGTAAGAACAAGATTGCAAAATGTCCTATAAGAACAAAACAGACTGGATACATTCTAAAATCAACACAAAATACATGTATCAGGAGTGGAAAACTTTTGCAAAAGAAGAGAATGGGTTCAGAAACTTCACTGGCAAAAGGTGAAAAAAGTAGCATGATTTTTTCACCCACTAAGGATTTATGCAAGCAGTATGTAGATAAAGACTGTCTTTATGTCCAGAAAGAGATTTCACCTGCAACCCCCACTATACAGAAGACTAGAAACACCATAAATACCTCTGTAGTAGCTAAGCAGAAGCATTGCAAAAAACACATCACAGCTGAAAATACGAAGAGCGGTTTGGTGTGTCTAACACAAGACCAACTACAACAGATTTTAATGACTGTGAACCAAGGAAATAAGTCTATTTCCGCGATTGAAAATGGAAAGGAGGAAACAAGTCAAGACAGTCTACATTTAAACAATACTTCCAATCAGCCAAAAGATGAGAACATAATGGGAGTATTCCAAAAAAATGAGGCTCTTTCATCTGTCCTGGATGAAAATAAATCCACTTTAAATAAAAATCAAGAGACATCTAAGCAGTATGAGCAGAAAATTGCCATAGAGAATGTATGGAAACCAGCTGACATATTCAGTACTCTGGGCGAAAGAGAACGTGACAGAAGTTTGTTGGAAGCAAAAAAAGCCCAGTGGAAGAAAGAGCTTGATGAACAGGTTGCTTTAAAGAAGAAAGAAAAAGAAGCTTCTGAAAAATGGAACAATCCTTGGAAAAAATTTGAAAGTGATAAAATAGTGTGGGAAAAATTTCAAACTCTTGGCCAGTCTAAGACTAGTCTTAGCTCTTCCAACATTCTGTCACAGTCTCCCAGTCAGATAACAGTGGTCCAGGCTGATGACTACCCACTATGTAGAGCCAGTCAGATTTTAGAGGAAACAGTACCACTGGAGCGCCCTTTGAGTACTGTGAAACAAGAACAGCAGAGAAAATGGATTGAAGATTTGAACAAGCAAATAGAAGATGATCGGCAAAGAAAAATAGAGGAAAAAATTACATCTTCAAAGTCTCAACTGTCCTCTCGGTCAATACACAACCAACCAGAGTACTTCTGTGTCTCTCCGGACACTCAGGAACTGTCTGATATCAGCAATGTTTATACACCAACGACTGGAAGCCAGGTTGAACCTTCAGAGGAGGAGCATATAGCAAAACCTGTTAGAGATATGGCTATGGCAAATAGTCAGAAAACAAACTTTCTCCGTTCTATGACTGCTCTCCTGGATCCAGCTCAGATTGAAGAACGTGACAGGCGACGACAAAAACAGTTAGAACATCAGAAAGCCATCACTGCTCAGGTAGAAGAGAAACGCAGGAAGAAGCAGCTGGAAGAACAGCAAAGAAAGAAGGAAGAACAAGAAGAGGAGCGTCGCTTAGCGAGGGAACGAGAAGAGATGCAGAAACAGTATGAAGAAGACATACTTAAGCAAAAACAAAAGGAAGAAATCATGACTCTCAAGACAAATGAATTGTTCCAGACAATGCAGAGAGCACAGGAGCTAGCACAGAGACTGAAACAAGAACAGAGAATACGAGAATTGGCTCAGAAGGGACATGATACGTCTGGATTGATTAAAAATCTTGGTGGATATGGCTTGGATGATGTCAGTGGTAAAATGAATACATGTATTAATTCTACAACCTCTCCCAAAAAGGATACTGCTGTACAAACAGATGACTTAAATACAGGAATGTTCACCATTGCAGAATCATGCTGTGGATCAATAATAGAGAGGGAAATTCTAAATTGCTCATCTCCTGAGATTCCTGCAGAATTTAATGACCAGTTTAAGAAAGACAAACAGGAACTAATCAATCAGGATAAAGCAGCCAACTTAGAAAAAGAAAACAGTTGGTACAATGATCAGTATGAGTTTGCAAGGACAGAGAAAAAACATATGAAGAAGTGTCCTAAAAGGCCTGATTGGAATATAAATAAGCCACTCAAAAGGTATATTCCGGCATCAGAAAAGTACCCTAAACAGCTTCAAAAGCAGAGAGAAGAAAAAAAAGTAAGAAGGCAGATGGAACTGCTTAATTTGGTAGAAAGAAATAATCCTGGACATCTCTCTCAAAATAGAGGGACTTCACCAGTTCTTCCTTCACCTCAAGAAGCAGAGGCAAGGTTCAGGTGGCATCTAATCAGAAAGGAGGAACCTCTGAAAAGTGATTCTTTCAGCAAGGAAAGGTCTCAGTCACCACTGGAACTGGTGAAAAACAGAACACAACAAACTCAGACACTAAAAAACAGAGAGAATCTGATCTTAGGAGACAGTCAGACAGAAACATCACCTGGAGCTTCTGAACCATCCCATTTTATCCCCTATGTCCGAACCAATGAGATTTATCATCTTGATCCAGATGCACCACTGTCCAGGCCTTTAACCCAGGATCTTCAGTACCAAAATCCACATGACTGTGACCAAGAACAATGGCAGCTATTTGAATCTGATGTCAGGGACCCACTTCTCAATCCTAACTTGGTGAAAAACAGGGATCGACAGCAAGCAATCCTTAAGGGACTATCAGAACTGAGACAGGGCCTTCTCCAGAAGCAAAGGGAGTTGGAAACTAATCTCATGCCTTTAGCTGCAAATCAAGAAGAGAATTTTAATTCTTCGTTTTAAATGTAGAAAATCAAATCCTTCACATTTGTTGCTTTTAAATTATGTGTTCATTGCATAACTGTATTTTCCA

**Supplementary Data 2.** Amino acid alignment of the annotated wild-type canine *CCDC66* isoforms.

Canis_I MNLGDGLKLETELLDGKTKLILSPYECKSKNSVKMGSKNKIAKCPIRTKQTGYILKSTQN 60

Canis_II ---------------------------------MMGSKNKIAKCPIRTKQTGYILKSTQN 27

Canis_III ------------------------------------------------------------ 0

Canis_IV ------------------------------------------------------------ 0

Canis_V MNLGDGLKLETELLDGKTKLILSPYECKSKNSVKMGSKNKIAKCPIRTKQTGYILKSTQN 60

Canis_VI ---------------------------------MMGSKNKIAKCPIRTKQTGYILKSTQN 27

Canis_VII MNLGDGLKLETELLDGKTKLILSPYECKSKNSVKMGSKNKIAKCPIRTKQTGYILKSTQN 60

Canis_VIII ---------------------------------MMGSKNKIAKCPIRTKQTGYILKSTQN 27

Canis_I TCIRSGKLLQKKRMGSETSLAKGEKSSMIFSPTKDLCKQYVDKDCLYVQKEISPATPTIQ 120

Canis_II TCIRSGKLLQKKRMGSETSLAKGEKSSMIFSPTKDLCKQYVDKDCLYVQKEISPATPTIQ 87

Canis_III ------------------------------------------------------------ 0

Canis_IV ------------------------------------------------------------ 0

Canis_V TCIRSGKLLQKKRMGSETSLAKG------------------------------------- 83

Canis_VI TCIRSGKLLQKKRMGSETSLAKG------------------------------------- 50

Canis_VII TCIRSGKLLQKKRMGSETSLAKGEKSSMIFSPTKDLCKQYVDKDCLYVQKEISPATPTIQ 120

Canis_VIII TCIRSGKLLQKKRMGSETSLAKGEKSSMIFSPTKDLCKQYVDKDCLYVQKEISPATPTIQ 87

Canis_I KTRNTINTSVVAKQKHCKKHITAENTKSGLVCLTQDQLQQILMTVNQGNKSISAIENGKE 180

Canis_II KTRNTINTSVVAKQKHCKKHITAENTKSGLVCLTQDQLQQILMTVNQGNKSISAIENGKE 147

Canis_III ------------------------------------------MTVNQGNKSISAIENGKE 18

Canis_IV ------------------------------------------MTVNQGNKSISAIENGKE 18

Canis_V ------------------------------------------------------------ 83

Canis_VI ------------------------------------------------------------ 50

Canis_VII KTRNTINTSVVAKQKHCKKHITAENTKSGLVCLTQDQLQQILMTVNQGNKSISAIENGKE 180

Canis_VIII KTRNTINTSVVAKQKHCKKHITAENTKSGLVCLTQDQLQQILMTVNQGNKSISAIENGKE 147

Canis_I ETSQDSLHLNNTSNQPKDENIMGVFQKNEALSSVLDENKSTLNKNQETSKQYEQKIAIEN 240

Canis_II ETSQDSLHLNNTSNQPKDENIMGVFQKNEALSSVLDENKSTLNKNQETSKQYEQKIAIEN 207

Canis_III ETSQDSLHLNNTSNQPKDENIMGVFQKNEALSSVLDENKSTLNKNQETSKQYEQKIAIEN 78

Canis_IV ETSQDSLHLNNTSNQPKDENIMGVFQKNEALSSVLDENKSTLNKNQETSKQYEQKIAIEN 78

Canis_V ---QDSLHLNNTSNQPKDENIMGVFQKNEALSSVLDENKSTLNKNQETSKQYEQKIAIEN 140

Canis_VI ---QDSLHLNNTSNQPKDENIMGVFQKNEALSSVLDENKSTLNKNQETSKQYEQKIAIEN 107

Canis_VII ETSQDSLHLNNTSNQPKDENIMGVFQKNEALSSVLDENKSTLNKNQETSKQYEQKIAIEN 240

Canis_VIII ETSQDSLHLNNTSNQPKDENIMGVFQKNEALSSVLDENKSTLNKNQETSKQYEQKIAIEN 207

*********************************************************

Canis_I VWKPADIFSTLGERERDRSLLEAKKAQWKKELDEQVALKKKEKEASEKWNNPWKKFESDK 300

Canis_II VWKPADIFSTLGERERDRSLLEAKKAQWKKELDEQVALKKKEKEASEKWNNPWKKFESDK 267

Canis_III VWKPADIFSTLGERERDRSLLEAKKAQWKKELDEQVALKKKEKEASEKWNNPWKKFESDK 138

Canis_IV VWKPADIFSTLGERERDRSLLEAKKAQWKKELDEQVALKKKEKEASEKWNNPWKKFESDK 138

Canis_V VWKPADIFSTLGERERDRSLLEAKKAQWKKELDEQVALKKKEKEASEKWNNPWKKFESDK 200

Canis_VI VWKPADIFSTLGERERDRSLLEAKKAQWKKELDEQVALKKKEKEASEKWNNPWKKFESDK 167

Canis_VII VWKPADIFSTLGERERDRSLLEAKKAQWKKELDEQVALKKKEKEASEKWNNPWKKFESDK 300

Canis_VIII VWKPADIFSTLGERERDRSLLEAKKAQWKKELDEQVALKKKEKEASEKWNNPWKKFESDK 267

************************************************************

Canis_I IVWEKFQTLGQSKTSLSSSNILSQSPSQITVVQADDYPLCRASQILEETVPLERPLSTVK 360

Canis_II IVWEKFQTLGQSKTSLSSSNILSQSPSQITVVQADDYPLCRASQILEETVPLERPLSTVK 327

Canis_III IVWEKFQTLGQSKTSLSSSNILSQSPSQITVVQADDYPLCRASQILEETVPLERPLSTVK 198

Canis_IV IVWEKFQTLGQSKTSLSSSNILSQSPSQITVVQADDYPLCRASQILEETVPLERPLSTVK 198

Canis_V IVWEKFQTLGQSKTSLSSSNILSQSPSQITVVQADDYPLCRASQILEETVPLERPLSTVK 260

Canis_VI IVWEKFQTLGQSKTSLSSSNILSQSPSQITVVQADDYPLCRASQILEETVPLERPLSTVK 227

Canis_VII IVWEKFQTLGQSKTSLSSSNILSQSPSQITVVQADDYPLCRASQILEETVPLERPLSTVK 360

Canis_VIII IVWEKFQTLGQSKTSLSSSNILSQSPSQITVVQADDYPLCRASQILEETVPLERPLSTVK 327

************************************************************

Canis_I QEQQRKWIEDLNKQIEDDRQRKIEEKITSSKGEEHDRWAMHFDSLKNYPASQSQLSSRSI 420

Canis_II QEQQRKWIEDLNKQIEDDRQRKIEEKITSSKGEEHDRWAMHFDSLKNYPASQSQLSSRSI 387

Canis_III QEQQRKWIEDLNKQIEDDRQRKIEEKITSSKGEEHDRWAMHFDSLKNYPASQSQLSSRSI 258

Canis_IV QEQQRKWIEDLNKQIEDDRQRKIEEKITSSKGEEHDRWAMHFDSLKNYPASQSQLSSRSI 258

Canis_V QEQQRKWIEDLNKQIEDDRQRKIEEKITSSKGEEHDRWAMHFDSLKNYPASQSQLSSRSI 320

Canis_VI QEQQRKWIEDLNKQIEDDRQRKIEEKITSSKGEEHDRWAMHFDSLKNYPASQSQLSSRSI 287

Canis_VII QEQQRKWIEDLNKQIEDDRQRKIEEKITSS---------------------KSQLSSRSI 399

Canis_VIII QEQQRKWIEDLNKQIEDDRQRKIEEKITSS---------------------KSQLSSRSI 366

****************************** :********

Canis_I HNQPEYFCVSPDTQELSDISNVYTPTTGSQVEPSEEEHIAKPVRDMAMANSQKTNFLRSM 480

Canis_II HNQPEYFCVSPDTQELSDISNVYTPTTGSQVEPSEEEHIAKPVRDMAMANSQKTNFLRSM 447

Canis_III HNQPEYFCVSPDTQELSDISNVYTPTTGSQVEPSEEEHIAKPVRDMAMANSQKTNFLRSM 318

Canis_IV HNQPEYFCVSPDTQELSDISNVYTPTTGSQVEPSEEEHIAKPVRDMAMANSQKTNFLRSM 318

Canis_V HNQPEYFCVSPDTQELSDISNVYTPTTGSQVEPSEEEHIAKPVRDMAMANSQKTNFLRSM 380

Canis_VI HNQPEYFCVSPDTQELSDISNVYTPTTGSQVEPSEEEHIAKPVRDMAMANSQKTNFLRSM 347

Canis_VII HNQPEYFCVSPDTQELSDISNVYTPTTGSQVEPSEEEHIAKPVRDMAMANSQKTNFLRSM 459

Canis_VIII HNQPEYFCVSPDTQELSDISNVYTPTTGSQVEPSEEEHIAKPVRDMAMANSQKTNFLRSM 426

************************************************************

Canis_I TALLDPAQIEERDRRRQKQLEHQKAITAQVEEKRRKKQLEEQQRKKEEQEEERRLARERE 540

Canis_II TALLDPAQIEERDRRRQKQLEHQKAITAQVEEKRRKKQLEEQQRKKEEQEEERRLARERE 507

Canis_III TALLDPAQIEERDRRRQKQLEHQKAITAQVEEKRRKKQLEEQQRKKEEQEEERRLARERE 378

Canis_IV TALLDPAQIEERDRRRQKQLEHQKAITAQVEEKRRKKQLEEQQRKKEEQEEERRLARERE 378

Canis_V TALLDPAQIEERDRRRQKQLEHQKAITAQVEEKRRKKQLEEQQRKKEEQEEERRLARERE 440

Canis_VI TALLDPAQIEERDRRRQKQLEHQKAITAQVEEKRRKKQLEEQQRKKEEQEEERRLARERE 407

Canis_VII TALLDPAQIEERDRRRQKQLEHQKAITAQVEEKRRKKQLEEQQRKKEEQEEERRLARERE 519

Canis_VIII TALLDPAQIEERDRRRQKQLEHQKAITAQVEEKRRKKQLEEQQRKKEEQEEERRLARERE 486

************************************************************

Canis_I EMQKQYEEDILKQKQKEEIMTLKTNELFQTMQRAQELAQRLKQEQRIRELAQKGHDTSGL 600

Canis_II EMQKQYEEDILKQKQKEEIMTLKTNELFQTMQRAQELAQRLKQEQRIRELAQKGHDTSGL 567

Canis_III EMQKQYEEDILKQKQKEEIMTLKTNELFQTMQRAQELAQRLKQEQRIRELAQKGHDTSGL 438

Canis_IV EMQKQYEEDILKQKQKEEIMTLKTNELFQTMQRAQELAQRLKQEQRIRELAQKGHDTSGL 438

Canis_V EMQKQYEEDILKQKQKEEIMTLKTNELFQTMQRAQELAQRLKQEQRIRELAQKGHDTSGL 500

Canis_VI EMQKQYEEDILKQKQKEEIMTLKTNELFQTMQRAQELAQRLKQEQRIRELAQKGHDTSGL 467

Canis_VII EMQKQYEEDILKQKQKEEIMTLKTNELFQTMQRAQELAQRLKQEQRIRELAQKGHDTSGL 579

Canis_VIII EMQKQYEEDILKQKQKEEIMTLKTNELFQTMQRAQELAQRLKQEQRIRELAQKGHDTSGL 546

************************************************************

Canis_I IKNLGGYGLDDVSGKMNTCINSTTSPKKDTAVQTDDLNTGMFTIAESCCGSIIEREILNC 660

Canis_II IKNLGGYGLDDVSGKMNTCINSTTSPKKDTAVQTDDLNTGMFTIAESCCGSIIEREILNC 627

Canis_III IKNLGGYGLDDVSGKMNTCINSTTSPKKDTAVQTDDLNTGMFTIAESCCGSIIEREILNC 498

Canis_IV IKNLGGYGLDDVSGKMNTCINSTTSPKKDTAVQTDDLNTGMFTIAESCCGSIIEREILNC 498

Canis_V IKNLGGYGLDDVSGKMNTCINSTTSPKKDTAVQTDDLNTGMFTIAESCCGSIIEREILNC 560

Canis_VI IKNLGGYGLDDVSGKMNTCINSTTSPKKDTAVQTDDLNTGMFTIAESCCGSIIEREILNC 527

Canis_VII IKNLGGYGLDDVSGKMNTCINSTTSPKKDTAVQTDDLNTGMFTIAESCCGSIIEREILNC 639

Canis_VIII IKNLGGYGLDDVSGKMNTCINSTTSPKKDTAVQTDDLNTGMFTIAESCCGSIIEREILNC 606

************************************************************

Canis_I SSPEIPAEFNDQFKKDKQELINQDKAANLEKENSWYNDQYEFARTEKKHMKKCPKRPDWN 720

Canis_II SSPEIPAEFNDQFKKDKQELINQDKAANLEKENSWYNDQYEFARTEKKHMKKCPKRPDWN 687

Canis_III SSPEIPAEFNDQFKKDKQELINQDKAANLEKENSWYNDQYEFARTEKKHMKKCPKRPDWN 558

Canis_IV SSPEIPAEFNDQFKKDKQELINQDKAANLEKENSWYNDQYEFARTEKKHMKKCPKRPDWN 558

Canis_V SSPEIPAEFNDQFKKDKQELINQDKAANLEKENSWYNDQYEFARTEKKHMKKCPKRPDWN 620

Canis_VI SSPEIPAEFNDQFKKDKQELINQDKAANLEKENSWYNDQYEFARTEKKHMKKCPKRPDWN 587

Canis_VII SSPEIPAEFNDQFKKDKQELINQDKAANLEKENSWYNDQYEFARTEKKHMKKCPKRPDWN 699

Canis_VIII SSPEIPAEFNDQFKKDKQELINQDKAANLEKENSWYNDQYEFARTEKKHMKKCPKRPDWN 666

************************************************************

Canis_I INKPLKRYIPASEKYPKQLQKQREEKKVRRQMELLNLVERNNPGHLSQNRGTSPVLPSPQ 780

Canis_II INKPLKRYIPASEKYPKQLQKQREEKKVRRQMELLNLVERNNPGHLSQNRGTSPVLPSPQ 747

Canis_III INKPLKRYIPASEKYPKQLQKQREEKKVRRQMELLNLVERNNPGHLSQNRGTSPVLPSPQ 618

Canis_IV INKPLKRYIPASEKYPKQLQKQREEKKVRRQMELLNLVERNNPGHLSQNRGTSPVLPSPQ 618

Canis_V INKPLKRYIPASEKYPKQLQKQREEKKVRRQMELLNLVERNNPGHLSQNRGTSPVLPSPQ 680

Canis_VI INKPLKRYIPASEKYPKQLQKQREEKKVRRQMELLNLVERNNPGHLSQNRGTSPVLPSPQ 647

Canis_VII INKPLKRYIPASEKYPKQLQKQREEKKVRRQMELLNLVERNNPGHLSQNRGTSPVLPSPQ 759

Canis_VIII INKPLKRYIPASEKYPKQLQKQREEKKVRRQMELLNLVERNNPGHLSQNRGTSPVLPSPQ 726

************************************************************

Canis_I EAEARFRWHLIRKEEPLKSDSFSKERSQSPLELVKNRTQQTQTLKNRENLILGDSQTETS 840

Canis_II EAEARFRWHLIRKEEPLKSDSFSKERSQSPLELVKNRTQQTQTLKNRENLILGDSQTETS 807

Canis_III EAEARFRWHLIRKEEPLKSDSFSKERSQSPLELVKNRTQQTQTLKNRENLILGDSQTETS 678

Canis_IV EAEARFRWHLIRKEEPLKSDSFSKERSQSPLELVKNRTQQTQTLKNRENLILGDSQTETS 678

Canis_V EAEARFRWHLIRKEEPLKSDSFSKERSQSPLELVKNRTQQTQTLKNRENLILGDSQTETS 740

Canis_VI EAEARFRWHLIRKEEPLKSDSFSKERSQSPLELVKNRTQQTQTLKNRENLILGDSQTETS 707

Canis_VII EAEARFRWHLIRKEEPLKSDSFSKERSQSPLELVKNRTQQTQTLKNRENLILGDSQTETS 819

Canis_VIII EAEARFRWHLIRKEEPLKSDSFSKERSQSPLELVKNRTQQTQTLKNRENLILGDSQTETS 786

************************************************************

Canis_I PGASEPSHFIPYVRTNEIYHLDPDAPLSRPLTQDLQYQNPHDCDQEQWQLFESDVRDPLL 900

Canis_II PGASEPSHFIPYVRTNEIYHLDPDAPLSRPLTQDLQYQNPHDCDQEQWQLFESDVRDPLL 867

Canis_III PGASEPSHFIPYVRTNEIYHLDPDAPLSRPLTQDLQYQNPHDCDQEQWQLFESDVRDPLL 738

Canis_IV PGASEPSHFIPYVRTNEIYHLDPDAPLSRPLTQDLQYQNPHDCDQEQWQLFESDVRDPLL 738

Canis_V PGASEPSHFIPYVRTNEIYHLDPDAPLSRPLTQDLQYQNPHDCDQEQWQLFESDVRDPLL 800

Canis_VI PGASEPSHFIPYVRTNEIYHLDPDAPLSRPLTQDLQYQNPHDCDQEQWQLFESDVRDPLL 767

Canis_VII PGASEPSHFIPYVRTNEIYHLDPDAPLSRPLTQDLQYQNPHDCDQEQWQLFESDVRDPLL 879

Canis_VIII PGASEPSHFIPYVRTNEIYHLDPDAPLSRPLTQDLQYQNPHDCDQEQWQLFESDVRDPLL 846

************************************************************

Canis_I NPNLVKNRDRQQAILKGLSELRQGLLQKQRELETNLMPLAANQEENFNSSF 951

Canis_II NPNLVKNRDRQQAILKGLSELRQGLLQKQRELETNLMPLAANQEENFNSSF 918

Canis_III NPNLVKNRDRQQAILKGLSELRQGLLQKQRELETNLMPLAANQEENFNSSF 789

Canis_IV NPNLVKNRDRQQAILKGLSELRQGLLQKQRELETNLMPLAANQEENFNSSF 789

Canis_V NPNLVKNRDRQQAILKGLSELRQGLLQKQRELETNLMPLAANQEENFNSSF 851

Canis_VI NPNLVKNRDRQQAILKGLSELRQGLLQKQRELETNLMPLAANQEENFNSSF 818

Canis_VII NPNLVKNRDRQQAILKGLSELRQGLLQKQRELETNLMPLAANQEENFNSSF 930

Canis_VIII NPNLVKNRDRQQAILKGLSELRQGLLQKQRELETNLMPLAANQEENFNSSF 897

***************************************************

**Supplementary Data 3.** Alignment of the annotated wild-type canine and human CCDC66 isoforms.

Canis_I MNLGDGLKLETELLDGKTKLILSPYECKSKNSVKMGSKNKIAKCPIRTKQTGYILKSTQN 60

Canis_II ---------------------------------MMGSKNKIAKCPIRTKQTGYILKSTQN 27

Canis_III ------------------------------------------------------------ 0

Canis_IV ------------------------------------------------------------ 0

Canis_V MNLGDGLKLETELLDGKTKLILSPYECKSKNSVKMGSKNKIAKCPIRTKQTGYILKSTQN 60

Canis_VI ---------------------------------MMGSKNKIAKCPIRTKQTGYILKSTQN 27

Canis_VII MNLGDGLKLETELLDGKTKLILSPYECKSKNSVKMGSKNKIAKCPIRTKQTGYILKSTQN 60

Canis_VIII ---------------------------------MMGSKNKIAKCPIRTKQTGYILKSTQN 27

Homo_1 MNLGDGLKLETELLDGKTKLILSPYEHKSKISVKMGNKAKIAKCPLRTK-TGHILKSTQD 59

Homo_2 ----------------------------------MGNKAKIAKCPLRTK-TGHILKSTQD 25

Homo_3 MNLGDGLKLETELLDGKTKLILSPYEHKSKISVKMGNKAKIAKCPLRTK-TGHILKSTQD 59

Homo_4 MNLGDGLKLETELLDGKTKLILSPYEHKSKISVKMGNKAKIAKCPLRTK-TGHILKSTQD 59

Homo_5 MNLGDGLKLETELLDGKTKLILSPYEHKSKISVKMGNKAKIAKCPLRTK-TGHILKSTQD 59

Homo_6 MNLGDGLKLETELLDGKTKLILSPYEHKSKISVKMGNKAKIAKCPLRTK-TGHILKSTQD 59

Homo_7 ---------------------------------MMGNKAKIAKCPLRTK-TGHILKSTQD 26

Homo_8 MNLGDGLKLETELLDGKTKLILSPYEHKSKISVKMGNKAKIAKCPLRTK-TGHILKSTQD 59

Homo_9 MNLGDGLKLETELLDGKTKLILSPYEHKSKISVKMGNKAKIAKCPLRTK-TGHILKSTQD 59

Homo_10 ------------------------------------------------------------ 0

Homo_11 ------------------------------------------------------------ 0

Homo_12 ------------------------------------------------------------ 0

Canis_I TCIRSGKLLQKKRMGSETSLAKGEKSSMIFSPTKDLCKQYVDKDCLYVQKEISPATPTIQ 120

Canis_II TCIRSGKLLQKKRMGSETSLAKGEKSSMIFSPTKDLCKQYVDKDCLYVQKEISPATPTIQ 87

Canis_III ------------------------------------------------------------ 0

Canis_IV ------------------------------------------------------------ 0

Canis_V TCIRSGKLLQKKRMGSETSLAKG------------------------------------- 83

Canis_VI TCIRSGKLLQKKRMGSETSLAKG------------------------------------- 50

Canis_VII TCIRSGKLLQKKRMGSETSLAKGEKSSMIFSPTKDLCKQYVDKDCLYVQKEISPATPTIQ 120

Canis_VIII TCIRSGKLLQKKRMGSETSLAKGEKSSMIFSPTKDLCKQYVDKDCLYVQKEISPATPTIQ 87

Homo_1 TCIGSEKLLQKKPVGSETSQAKGEKNGMTFSSTKDLCKQCIDKDCLHIQKEISPATPNMQ 119

Homo_2 TCIGSEKLLQKKPVGSETSQAKGEKNGMTFSSTKDLCKQCIDKDCLHIQKEISPATPNMQ 85

Homo_3 TCIGSEKLLQKKPVGSETSQAKGEKNGMTFSSTKDLCKQCIDKDCLHIQKEISPATPNMQ 119

Homo_4 TCIGSEKLLQKKPVGSETSQAKGEKNGMTFSSTKDLCKQCIDKDCLHIQKEISPATPNMQ 119

Homo_5 TCIGSEKLLQKKPVGSETSQAKGEKNGMTFSSTKDLCKQCIDKDCLHIQKEISPATPNMQ 119

Homo_6 TCIGSEKLLQKKPVGSETSQAKGEKNGMTFSSTKDLCKQCIDKDCLHIQKEISPATPNMQ 119

Homo_7 TCIGSEKLLQKKPVGSETSQAKGEKNGMTFSSTKDLCKQCIDKDCLHIQKEISPATPNMQ 86

Homo_8 TCIGSEKLLQKKPVGSETSQAKGEKNGMTFSSTKDLCKQCIDKDCLHIQKEISPATPNMQ 119

Homo_9 TCIGSEKLLQKKPVGSETSQAKGEKNGMTFSSTKDLCKQCIDKDCLHIQKEISPATPNMQ 119

Homo_10 ------------------------------------------------------------ 0

Homo_11 ------------------------------------------------------------ 0

Homo_12 ------------------------------------------------------------ 0

Canis_I KTRNTINTSVVAKQKHCKKHITAENTKSGLVCLTQDQLQQILMTVNQGNKSISAIENGKE 180

Canis_II KTRNTINTSVVAKQKHCKKHITAENTKSGLVCLTQDQLQQILMTVNQGNKSISAIENGKE 147

Canis_III ------------------------------------------MTVNQGNKSISAIENGKE 18

Canis_IV ------------------------------------------MTVNQGNKSISAIENGKE 18

Canis_V ------------------------------------------------------------ 83

Canis_VI ------------------------------------------------------------ 50

Canis_VII KTRNTINTSVVAKQKHCKKHITAENTKSGLVCLTQDQLQQILMTVNQGNKSISAIENGKE 180

Canis_VIII KTRNTINTSVVAKQKHCKKHITAENTKSGLVCLTQDQLQQILMTVNQGNKSISAIENGKE 147

Homo_1 KTRNTVNTSLVGKQKPHKKHITAENMKSSLVCLTQDQLQQILMTVNQGNRSLSLTENGKE 179

Homo_2 KTRNTVNTSLVGKQKPHKKHITAENMKSSLVCLTQDQLQQILMTVNQGNRSLSLTENGKE 145

Homo_3 KTRNTVNTSLVGKQKPHKKHITAENMKSSLVCLTQDQLQQILMTVNQGNRSLSLTENGKE 179

Homo_4 KTRNTVNTSLVGKQKPHKKHITAENMKSSLVCLTQDQLQQILMTVNQGNRSLSLTENGKE 179

Homo_5 KTRNTVNTSLVGKQKPHKKHITAENMKSSLVCLTQDQLQQILMTVNQGNRSLSLTENGKE 179

Homo_6 KTRNTVNTSLVGKQKPHKKHITAENMKSSLVCLTQDQLQQILMTVNQGNRSLSLTENGKE 179

Homo_7 KTRNTVNTSLVGKQKPHKKHITAENMKSSLVCLTQDQLQQILMTVNQGNRSLSLTENGKE 146

Homo_8 KTRNTVNTSLVGKQKPHKKHITAENMKSSLVCLTQDQLQQILMTVNQGNRSLSLTENGKE 179

Homo_9 KTRNTVNTSLVGKQKPHKKHITAENMKSSLVCLTQDQLQQILMTVNQGNRSLSLTENGKE 179

Homo_10 ------------------------------------------------------------ 0

Homo_11 ------------------------------------------------------------ 0

Homo_12 ------------------------------------------------------------ 0

Canis_I ETSQDSLHLNNTSNQPKDENIMGVFQKNEALSSVLDENKSTLNKNQETSKQYEQKIAIEN 240

Canis_II ETSQDSLHLNNTSNQPKDENIMGVFQKNEALSSVLDENKSTLNKNQETSKQYEQKIAIEN 207

Canis_III ETSQDSLHLNNTSNQPKDENIMGVFQKNEALSSVLDENKSTLNKNQETSKQYEQKIAIEN 78

Canis_IV ETSQDSLHLNNTSNQPKDENIMGVFQKNEALSSVLDENKSTLNKNQETSKQYEQKIAIEN 78

Canis_V ---QDSLHLNNTSNQPKDENIMGVFQKNEALSSVLDENKSTLNKNQETSKQYEQKIAIEN 140

Canis_VI ---QDSLHLNNTSNQPKDENIMGVFQKNEALSSVLDENKSTLNKNQETSKQYEQKIAIEN 107

Canis_VII ETSQDSLHLNNTSNQPKDENIMGVFQKNEALSSVLDENKSTLNKNQETSKQYEQKIAIEN 240

Canis_VIII ETSQDSLHLNNTSNQPKDENIMGVFQKNEALSSVLDENKSTLNKNQETSKQYEQKIAIEN 207

Homo_1 AKSQYSLYLNSISNQPKDENIMGLFKKTEMVSSVPAENKSVLNEHQETSKQCEQKIAIEN 239

Homo_2 AKSQYSLYLNSISNQPKDENIMGLFKKTEMVSSVPAENKSVLNEHQETSKQCEQKIAIEN 205

Homo_3 AKSQYSLYLNSISNQPKDENIMGLFKKTEMVSSVPAENKSVLNEHQETSKQCEQKIAIEN 239

Homo_4 AKSQYSLYLNSISNQPKDENIMGLFKKTEMVSSVPAENKSVLNEHQETSKQCEQKIAIEN 239

Homo_5 AKSQYSLYLNSISNQPKDENIMGLFKKTEMVSSVPAENKSVLNEHQETSKQ-------EN 232

Homo_6 AKSQYSLYLNSISNQPKDENIMGLFKKTEMVSSVPAENKSVLNEHQETSKQCEQKIAIEN 239

Homo_7 AKSQYSLYLNSISNQPKDENIMGLFKKTEMVSSVPAENKSVLNEHQETSKQCEQKIAIEN 206

Homo_8 AKSQYSLYLNSISNQPKDENIMGLFKKTEMVSSVPAENKSVLNEHQETSKQCEQKIAIEN 239

Homo_9 AKSQYSLYLNSISNQPKDENIMGLFKKTEMVSSVPAENKSVLNEHQETSKQ-------EN 232

Homo_10 ------------------------------------------------------------ 0

Homo_11 ------------------------------------------------------------ 0

Homo_12 ------------------------------------------------------------ 0

Canis_I VWKPADIFSTLGERERDRSLLEAKKAQWKKELDEQVALKKKEKEASEKWNNPWKKFESDK 300

Canis_II VWKPADIFSTLGERERDRSLLEAKKAQWKKELDEQVALKKKEKEASEKWNNPWKKFESDK 267

Canis_III VWKPADIFSTLGERERDRSLLEAKKAQWKKELDEQVALKKKEKEASEKWNNPWKKFESDK 138

Canis_IV VWKPADIFSTLGERERDRSLLEAKKAQWKKELDEQVALKKKEKEASEKWNNPWKKFESDK 138

Canis_V VWKPADIFSTLGERERDRSLLEAKKAQWKKELDEQVALKKKEKEASEKWNNPWKKFESDK 200

Canis_VI VWKPADIFSTLGERERDRSLLEAKKAQWKKELDEQVALKKKEKEASEKWNNPWKKFESDK 167

Canis_VII VWKPADIFSTLGERERDRSLLEAKKAQWKKELDEQVALKKKEKEASEKWNNPWKKFESDK 300

Canis_VIII VWKPADIFSTLGERERDRSLLEAKKAQWKKELDEQVALKKKEKEASEKWNNPWKKFESDK 267

Homo_1 EWKPADIFSTLGERECDRSSLEAKKAQWRKELDEQVALKKKEKEVSEKWNDPWKKSESDK 299

Homo_2 EWKPADIFSTLGERECDRSSLEAKKAQWRKELDEQVALKKKEKEVSEKWNDPWKKSESDK 265

Homo_3 EWKPADIFSTLGERECDRSSLEAKKAQWRKELDEQVALKKKEKEVSEKWNDPWKKSESDK 299

Homo_4 EWKPADIFSTLGERECDRSSLEAKKAQWRKELDEQVALKKKEKEVSEKWNDPWKKSESDK 299

Homo_5 EWKPADIFSTLGERECDRSSLEAKKAQWRKELDEQVALKKKEKEVSEKWNDPWKKSESDK 292

Homo_6 EWKPADIFSTLGERECDRSSLEAKKAQWRKELDEQVALKKKEKEVSEKWNDPWKKSESDK 299

Homo_7 EWKPADIFSTLGERECDRSSLEAKKAQWRKELDEQVALKKKEKEVSEKWNDPWKKSESDK 266

Homo_8 EWKPADIFSTLGERECDRSSLEAKKAQWRKELDEQVALKKKEKEVSEKWNDPWKKSESDK 299

Homo_9 EWKPADIFSTLGERECDRSSLEAKKAQWRKELDEQVALKKKEKEVSEKWNDPWKKSESDK 292

Homo_10 ------------------------------------------------------------ 0

Homo_11 ------------------------------------------------------------ 0

Homo_12 ------------------------------------------------------------ 0

Canis_I IVWEKFQTLGQSKTSLSSSNILSQSPSQITVVQADDYPLCRASQILEETVPLERPLSTVK 360

Canis_II IVWEKFQTLGQSKTSLSSSNILSQSPSQITVVQADDYPLCRASQILEETVPLERPLSTVK 327

Canis_III IVWEKFQTLGQSKTSLSSSNILSQSPSQITVVQADDYPLCRASQILEETVPLERPLSTVK 198

Canis_IV IVWEKFQTLGQSKTSLSSSNILSQSPSQITVVQADDYPLCRASQILEETVPLERPLSTVK 198

Canis_V IVWEKFQTLGQSKTSLSSSNILSQSPSQITVVQADDYPLCRASQILEETVPLERPLSTVK 260

Canis_VI IVWEKFQTLGQSKTSLSSSNILSQSPSQITVVQADDYPLCRASQILEETVPLERPLSTVK 227

Canis_VII IVWEKFQTLGQSKTSLSSSNILSQSPSQITVVQADDYPLCRASQILEETVPLERPLSTVK 360

Canis_VIII IVWEKFQTLGQSKTSLSSSNILSQSPSQITVVQADDYPLCRASQILEETVPLERPLSTVK 327

Homo_1 IIWEKHQILD----------------------------------QSRETVLLEHPFSAVK 325

Homo_2 IIWEKHQILD----------------------------------QSRETVLLEHPFSAVK 291

Homo_3 IIWEKHQILD----------------------------------QSRETVLLEHPFSAVK 325

Homo_4 IIWEKHQILD----------------------------------QSRETVLLEHPFSAVK 325

Homo_5 IIWEKHQILD----------------------------------QSRETVLLEHPFSAVK 318

Homo_6 IIWEKHQILD----------------------------------QSRETVLLEHPFSAVK 325

Homo_7 IIWEKHQILD----------------------------------QSRETVLLEHPFSAVK 292

Homo_8 IIWEKHQILD----------------------------------QSRETVLLEHPFSAVK 325

Homo_9 IIWEKHQILD----------------------------------QSRETVLLEHPFSAVK 318

Homo_10 MGHSQLRASQ----------------------------------TLEETVLLEHPFSAVK 26

Homo_11 MGHSQLRASQ----------------------------------TLEETVLLEHPFSAVK 26

Homo_12 ------------------------------------------------------------ 0

Canis_I QEQQRKWIEDLNKQIEDDRQRKIEEKITSSKGEEHDRWAMHFDSLKNYPASQSQLSSRSI 420

Canis_II QEQQRKWIEDLNKQIEDDRQRKIEEKITSSKGEEHDRWAMHFDSLKNYPASQSQLSSRSI 387

Canis_III QEQQRKWIEDLNKQIEDDRQRKIEEKITSSKGEEHDRWAMHFDSLKNYPASQSQLSSRSI 258

Canis_IV QEQQRKWIEDLNKQIEDDRQRKIEEKITSSKGEEHDRWAMHFDSLKNYPASQSQLSSRSI 258

Canis_V QEQQRKWIEDLNKQIEDDRQRKIEEKITSSKGEEHDRWAMHFDSLKNYPASQSQLSSRSI 320

Canis_VI QEQQRKWIEDLNKQIEDDRQRKIEEKITSSKGEEHDRWAMHFDSLKNYPASQSQLSSRSI 287

Canis_VII QEQQRKWIEDLNKQIEDDRQRKIEEKITSS---------------------KSQLSSRSI 399

Canis_VIII QEQQRKWIEDLNKQIEDDRQRKIEEKITSS---------------------KSQLSSRSI 366

Homo_1 QELQRKWIEELNKQIEDDRQRKIEEKIIYSKGEEHDRWAMHFDSLKSYPGSQSQLFSQST 385

Homo_2 QELQRKWIEELNKQIEDDRQRKIEEKIIYSKGEEHDRWAMHFDSLKSYPGSQSQLFSQST 351

Homo_3 QELQRKWIEELNKQIEDDRQRKIEEKIIYSKGEEHDRWAMHFDSLKSYPGSQSQLFSQST 385

Homo_4 QELQRKWIEELNKQIEDDRQRKIEEKIIYSKGEEHDRWAMHFDSLKSYPGSQSQLFSQST 385

Homo_5 QELQRKWIEELNKQIEDDRQRKIEEKIIYSKGEEHDRWAMHFDSLKSYPGSQSQLFSQST 378

Homo_6 QELQRKWIEELNKQIEDDRQRKIEEKIIYS-----------------------KLFSQST 362

Homo_7 QELQRKWIEELNKQIEDDRQRKIEEKIIYSKGEEHDRWAMHFDSLKSYPGSQSQLFSQST 352

Homo_8 QELQRKWIEELNKQIEDDRQRKIEEKIIYSK----------------------------- 356

Homo_9 QELQRKWIEELNKQIEDDRQRKIEEKIIYSK----------------------------- 349

Homo_10 QELQRKWIEELNKQIEDDRQRKIEEKIIYSKGEEHDRWAMHFDSLKSYPGSQSQLFSQST 86

Homo_11 QELQRKWIEELNKQIEDDRQRKIEEKIIYSK----------------------------- 57

Homo_12 ---------------------------------------MHFDSLKSYPGSQSQLFSQST 21

Canis_I HNQPEYFCVSPDTQELSDISNVYTPTTGSQVEPSEEEHIAKPVRDMAMANSQKTNFLRSM 480

Canis_II HNQPEYFCVSPDTQELSDISNVYTPTTGSQVEPSEEEHIAKPVRDMAMANSQKTNFLRSM 447

Canis_III HNQPEYFCVSPDTQELSDISNVYTPTTGSQVEPSEEEHIAKPVRDMAMANSQKTNFLRSM 318

Canis_IV HNQPEYFCVSPDTQELSDISNVYTPTTGSQVEPSEEEHIAKPVRDMAMANSQKTNFLRSM 318

Canis_V HNQPEYFCVSPDTQELSDISNVYTPTTGSQVEPSEEEHIAKPVRDMAMANSQKTNFLRSM 380

Canis_VI HNQPEYFCVSPDTQELSDISNVYTPTTGSQVEPSEEEHIAKPVRDMAMANSQKTNFLRSM 347

Canis_VII HNQPEYFCVSPDTQELSDISNVYTPTTGSQVEPSEEEHIAKPVRDMAMANSQKTNFLRSM 459

Canis_VIII HNQPEYFCVSPDTQELSDISNVYTPTTGSQVEPSEEEHIAKPVRDMAMANSQKTNFLRSM 426

Homo_1 HKQPEYFCVSPDTQELADVSSVCTPTTGSQVEPSEEEHIAKPIKDVVMANSKKTNFLRSM 445

Homo_2 HKQPEYFCVSPDTQELADVSSVCTPTTGSQVEPSEEEHIAKPIKDVVMANSKKTNFLRSM 411

Homo_3 HKQPEYFCVSPDTQELADVSSVCTPTTGSQVEPSEEEHIAKPIKDVVMANSKKTNFLRSM 445

Homo_4 HKQPEYFCVSPDTQELADVSSVCTPTTGSQVEPSEEEHIAKPIKDVVMANSKKTNFLRSM 445

Homo_5 HKQPEYFCVSPDTQELADVSSVCTPTTGSQVEPSEEEHIAKPIKDVVMANSKKTNFLRSM 438

Homo_6 HKQPEYFCVSPDTQELADVSSVCTPTTGSQVEPSEEEHIAKPIKDVVMANSKKTNFLRSM 422

Homo_7 HKQPEYFCVSPDTQELADVSSVCTPTTGSQVEPSEEEHIAKPIKDVVMANSKKTNFLRSM 412

Homo_8 --------------ELADVSSVCTPTTGSQVEPSEEEHIAKPIKDVVMANSKKTNFLRSM 402

Homo_9 --------------ELADVSSVCTPTTGSQVEPSEEEHIAKPIKDVVMANSKKTNFLRSM 395

Homo_10 HKQPEYFCVSPDTQELADVSSVCTPTTGSQVEPSEEEHIAKPIKDVVMANSKKTNFLRSM 146

Homo_11 --------------ELADVSSVCTPTTGSQVEPSEEEHIAKPIKDVVMANSKKTNFLRSM 103

Homo_12 HKQPEYFCVSPDTQELADVSSVCTPTTGSQVEPSEEEHIAKPIKDVVMANSKKTNFLRSM 81

**:*:*.* *******************::*:.****:********

Canis_I TALLDPAQIEERDRRRQKQLEHQKAITAQVEEKRRKKQLEEQQRKKEEQEEERRLARERE 540

Canis_II TALLDPAQIEERDRRRQKQLEHQKAITAQVEEKRRKKQLEEQQRKKEEQEEERRLARERE 507

Canis_III TALLDPAQIEERDRRRQKQLEHQKAITAQVEEKRRKKQLEEQQRKKEEQEEERRLARERE 378

Canis_IV TALLDPAQIEERDRRRQKQLEHQKAITAQVEEKRRKKQLEEQQRKKEEQEEERRLARERE 378

Canis_V TALLDPAQIEERDRRRQKQLEHQKAITAQVEEKRRKKQLEEQQRKKEEQEEERRLARERE 440

Canis_VI TALLDPAQIEERDRRRQKQLEHQKAITAQVEEKRRKKQLEEQQRKKEEQEEERRLARERE 407

Canis_VII TALLDPAQIEERDRRRQKQLEHQKAITAQVEEKRRKKQLEEQQRKKEEQEEERRLARERE 519

Canis_VIII TALLDPAQIEERDRRRQKQLEHQKAITAQVEEKRRKKQLEEQQRKKEEQEEERRLARERE 486

Homo_1 TALLDPAQIEERDRRRQKQLEHQKAITAQVEEKRRKKQLEEEQRKKEEQEEELRLAQERE 505

Homo_2 TALLDPAQIEERDRRRQKQLEHQKAITAQVEEKRRKKQLEEEQRKKEEQEEELRLAQERE 471

Homo_3 TALLDPAQIEERDRRRQKQLEHQKAITAQVEEKRRKKQLEEEQRKKEEQEEELRLAQERE 505

Homo_4 TALLDPAQIEERDRRRQKQLEH------QVEEKRRKKQLEEEQRKKEEQEEELRLAQERE 499

Homo_5 TALLDPAQIEERDRRRQKQLEHQKAITAQVEEKRRKKQLEEEQRKKEEQEEELRLAQERE 498

Homo_6 TALLDPAQIEERDRRRQKQLEHQKAITAQVEEKRRKKQLEEEQRKKEEQEEELRLAQERE 482

Homo_7 TALLDPAQIEERDRRRQKQLEHQKAITAQVEEKRRKKQLEEEQRKKEEQEEELRLAQERE 472

Homo_8 TALLDPAQIEERDRRRQKQLEHQKAITAQVEEKRRKKQLEEEQRKKEEQEEELRLAQERE 462

Homo_9 TALLDPAQIEERDRRRQKQLEHQKAITAQVEEKRRKKQLEEEQRKKEEQEEELRLAQERE 455

Homo_10 TALLDPAQIEERDRRRQKQLEHQKAITAQVEEKRRKKQLEEEQRKKEEQEEELRLAQERE 206

Homo_11 TALLDPAQIEERDRRRQKQLEHQKAITAQVEEKRRKKQLEEEQRKKEEQEEELRLAQERE 163

Homo_12 TALLDPAQIEERDRRRQKQLEHQKAITAQVEEKRRKKQLEEEQRKKEEQEEELRLAQERE 141

********************** *************:********** ***:***

Canis_I EMQKQYEEDILKQKQKEEIMTLKTNELFQTMQRAQELAQRLKQEQRIRELAQKGHDTSGL 600

Canis_II EMQKQYEEDILKQKQKEEIMTLKTNELFQTMQRAQELAQRLKQEQRIRELAQKGHDTSGL 567

Canis_III EMQKQYEEDILKQKQKEEIMTLKTNELFQTMQRAQELAQRLKQEQRIRELAQKGHDTSGL 438

Canis_IV EMQKQYEEDILKQKQKEEIMTLKTNELFQTMQRAQELAQRLKQEQRIRELAQKGHDTSGL 438

Canis_V EMQKQYEEDILKQKQKEEIMTLKTNELFQTMQRAQELAQRLKQEQRIRELAQKGHDTSGL 500

Canis_VI EMQKQYEEDILKQKQKEEIMTLKTNELFQTMQRAQELAQRLKQEQRIRELAQKGHDTSGL 467

Canis_VII EMQKQYEEDILKQKQKEEIMTLKTNELFQTMQRAQELAQRLKQEQRIRELAQKGHDTSGL 579

Canis_VIII EMQKQYEEDILKQKQKEEIMTLKTNELFQTMQRAQELAQRLKQEQRIRELAQKGHDTSGL 546

Homo_1 EMQKQYEEDILKQKQKEEIMTLKTNELFQTMQRAQELAQRLKQEQRIRELAQKGHDTSRL 565

Homo_2 EMQKQYEEDILKQKQKEEIMTLKTNELFQTMQRAQELAQRLKQEQRIRELAQKGHDTSRL 531

Homo_3 EMQKQYEEDILKQKQKEEIMTLKTNELFQTMQRAQELAQRLKQEQRIRELAQKGHDTSRL 565

Homo_4 EMQKQYEEDILKQKQKEEIMTLKTNELFQTMQRAQELAQRLKQEQRIRELAQKGHDTSRL 559

Homo_5 EMQKQYEEDILKQKQKEEIMTLKTNELFQTMQRAQELAQRLKQEQRIRELAQKGHDTSRL 558

Homo_6 EMQKQYEEDILKQKQKEEIMTLKTNELFQTMQRAQELAQRLKQEQRIRELAQKGHDTSRL 542

Homo_7 EMQKQYEEDILKQKQKEEIMTLKTNELFQTMQRAQELAQRLKQEQRIRELAQKGHDTSRL 532

Homo_8 EMQKQYEEDILKQKQKEEIMTLKTNELFQTMQRAQELAQRLKQEQRIRELAQKGHDTSRL 522

Homo_9 EMQKQYEEDILKQKQKEEIMTLKTNELFQTMQRAQELAQRLKQEQRIRELAQKGHDTSRL 515

Homo_10 EMQKQYEEDILKQKQKEEIMTLKTNELFQTMQRAQELAQRLKQEQRIRELAQKGHDTSRL 266

Homo_11 EMQKQYEEDILKQKQKEEIMTLKTNELFQTMQRAQELAQRLKQEQRIRELAQKGHDTSRL 223

Homo_12 EMQKQYEEDILKQKQKEEIMTLKTNELFQTMQRAQELAQRLKQEQRIRELAQKGHDTSRL 201

********************************************************** *

Canis_I IKNLGGYG----------------------LDDVSGKMNTCINSTTSPKKDTAVQTDDLN 638

Canis_II IKNLGGYG----------------------LDDVSGKMNTCINSTTSPKKDTAVQTDDLN 605

Canis_III IKNLGGYG----------------------LDDVSGKMNTCINSTTSPKKDTAVQTDDLN 476

Canis_IV IKNLGGYG----------------------LDDVSGKMNTCINSTTSPKKDTAVQTDDLN 476

Canis_V IKNLGGYG----------------------LDDVSGKMNTCINSTTSPKKDTAVQTDDLN 538

Canis_VI IKNLGGYG----------------------LDDVSGKMNTCINSTTSPKKDTAVQTDDLN 505

Canis_VII IKNLGGYG----------------------LDDVSGKMNTCINSTTSPKKDTAVQTDDLN 617

Canis_VIII IKNLGGYG----------------------LDDVSGKMNTCINSTTSPKKDTAVQTDDLN 584

Homo_1 IKNLG------VDTIQMEYNASNISNSRHDSDEISGKMNTYMNSTT-SKKDTGVQTDDLN 618

Homo_2 IKNLG------VDTIQMEYNASNISNSRHDSDEISGKMNTYMNSTT-SKKDTGVQTDDLN 584

Homo_3 IKNLGGDFSLPVDTIQMEYNASNISNSRHDSDEISGKMNTYMNSTT-SKKDTGVQTDDLN 624

Homo_4 IKNLG------VDTIQMEYNASNISNSRHDSDEISGKMNTYMNSTT-SKKDTGVQTDDLN 612

Homo_5 IKNLG------VDTIQMEYNASNISNSRHDSDEISGKMNTYMNSTT-SKKDTGVQTDDLN 611

Homo_6 IKNLG------VDTIQMEYNASNISNSRHDSDEISGKMNTYMNSTT-SKKDTGVQTDDLN 595

Homo_7 IKNLG------VDTIQMEYNASNISNSRHDSDEISGKMNTYMNSTT-SKKDTGVQTDDLN 585

Homo_8 IKNLG------VDTIQMEYNASNISNSRHDSDEISGKMNTYMNSTT-SKKDTGVQTDDLN 575

Homo_9 IKNLG------VDTIQMEYNASNISNSRHDSDEISGKMNTYMNSTT-SKKDTGVQTDDLN 568

Homo_10 IKNLG------VDTIQMEYNASNISNSRHDSDEISGKMNTYMNSTT-SKKDTGVQTDDLN 319

Homo_11 IKNLG------VDTIQMEYNASNISNSRHDSDEISGKMNTYMNSTT-SKKDTGVQTDDLN 276

Homo_12 IKNLG------VDTIQMEYNASNISNSRHDSDEISGKMNTYMNSTT-SKKDTGVQTDDLN 254

***** *::****** :**** ****.*******

Canis_I TGMFTIAESCCGSIIEREILNCSSPEIPAEFNDQFKKDK-QELINQDKAANLEKENSWYN 697

Canis_II TGMFTIAESCCGSIIEREILNCSSPEIPAEFNDQFKKDK-QELINQDKAANLEKENSWYN 664

Canis_III TGMFTIAESCCGSIIEREILNCSSPEIPAEFNDQFKKDK-QELINQDKAANLEKENSWYN 535

Canis_IV TGMFTIAESCCGSIIEREILNCSSPEIPAEFNDQFKKDK-QELINQDKAANLEKENSWYN 535

Canis_V TGMFTIAESCCGSIIEREILNCSSPEIPAEFNDQFKKDK-QELINQDKAANLEKENSWYN 597

Canis_VI TGMFTIAESCCGSIIEREILNCSSPEIPAEFNDQFKKDK-QELINQDKAANLEKENSWYN 564

Canis_VII TGMFTIAESCCGSIIEREILNCSSPEIPAEFNDQFKKDK-QELINQDKAANLEKENSWYN 676

Canis_VIII TGMFTIAESCCGSIIEREILNCSSPEIPAEFNDQFKKDK-QELINQDKAANLEKENSWYN 643

Homo_1 IGIFTNAESHCGSLMERDITNCSSPEISAELIGQFSTKKNKQELTQDKGASLEKENNRCN 678

Homo_2 IGIFTNAESHCGSLMERDITNCSSPEISAELIGQFSTKKNKQELTQDKGASLEKENNRCN 644

Homo_3 IGIFTNAESHCGSLMERDITNCSSPEISAELIGQFSTKKNKQELTQDKGASLEKENNRCN 684

Homo_4 IGIFTNAESHCGSLMERDITNCSSPEISAELIGQFSTKKNKQELTQDKGASLEKENNRCN 672

Homo_5 IGIFTNAESHCGSLMERDITNCSSPEISAELIGQFSTKKNKQELTQDKGASLEKENNRCN 671

Homo_6 IGIFTNAESHCGSLMERDITNCSSPEISAELIGQFSTKKNKQELTQDKGASLEKENNRCN 655

Homo_7 IGIFTNAESHCGSLMERDITNCSSPEISAELIGQFSTKKNKQELTQDKGASLEKENNRCN 645

Homo_8 IGIFTNAESHCGSLMERDITNCSSPEISAELIGQFSTKKNKQELTQDKGASLEKENNRCN 635

Homo_9 IGIFTNAESHCGSLMERDITNCSSPEISAELIGQFSTKKNKQELTQDKGASLEKENNRCN 628

Homo_10 IGIFTNAESHCGSLMERDITNCSSPEISAELIGQFSTKKNKQELTQDKGASLEKENNRCN 379

Homo_11 IGIFTNAESHCGSLMERDITNCSSPEISAELIGQFSTKKNKQELTQDKGASLEKENNRCN 336

Homo_12 IGIFTNAESHCGSLMERDITNCSSPEISAELIGQFSTKKNKQELTQDKGASLEKENNRCN 314

*:** *** ***::**:* ******* **: .**...* :: :.***.*.*****. *

Canis_I DQY---EFARTEKKHMKKCPKRPDWNINKPLKRYIPASEKYPKQLQKQREEKKVRRQMEL 754

Canis_II DQY---EFARTEKKHMKKCPKRPDWNINKPLKRYIPASEKYPKQLQKQREEKKVRRQMEL 721

Canis_III DQY---EFARTEKKHMKKCPKRPDWNINKPLKRYIPASEKYPKQLQKQREEKKVRRQMEL 592

Canis_IV DQY---EFARTEKKHMKKCPKRPDWNINKPLKRYIPASEKYPKQLQKQREEKKVRRQMEL 592

Canis_V DQY---EFARTEKKHMKKCPKRPDWNINKPLKRYIPASEKYPKQLQKQREEKKVRRQMEL 654

Canis_VI DQY---EFARTEKKHMKKCPKRPDWNINKPLKRYIPASEKYPKQLQKQREEKKVRRQMEL 621

Canis_VII DQY---EFARTEKKHMKKCPKRPDWNINKPLKRYIPASEKYPKQLQKQREEKKVRRQMEL 733

Canis_VIII DQY---EFARTEKKHMKKCPKRPDWNINKPLKRYIPASEKYPKQLQKQREEKKVRRQMEL 700

Homo_1 DQCNQFTRIEKQTKHMKKYPKRPDWNINKPPKRYIPASEKYPKQLQKQREEKKVRRQMEL 738

Homo_2 DQCNQFTRIEKQTKHMKKYPKRPDWNINKPPKRYIPASEKYPKQLQKQREEKKVRRQMEL 704

Homo_3 DQCNQFTRIEKQTKHMKKYPKRPDWNINKPPKRYIPASEKYPKQLQKQREEKKVRRQMEL 744

Homo_4 DQCNQFTRIEKQTKHMKKYPKRPDWNINKPPKRYIPASEKYPKQLQKQREEKKVRRQMEL 732

Homo_5 DQCNQFTRIEKQTKHMKKYPKRPDWNINKPPKRYIPASEKYPKQLQKQREEKKVRRQMEL 731

Homo_6 DQCNQFTRIEKQTKHMKKYPKRPDWNINKPPKRYIPASEKYPKQLQKQREEKKVRRQMEL 715

Homo_7 DQCNQFTRIEKQTKHMKKYPKRPDWNINKPPKRYIPASEKYPKQLQKQREEKKVRRQMEL 705

Homo_8 DQCNQFTRIEKQTKHMKKYPKRPDWNINKPPKRYIPASEKYPKQLQKQREEKKVRRQMEL 695

Homo_9 DQCNQFTRIEKQTKHMKKYPKRPDWNINKPPKRYIPASEKYPKQLQKQREEKKVRRQMEL 688

Homo_10 DQCNQFTRIEKQTKHMKKYPKRPDWNINKPPKRYIPASEKYPKQLQKQREEKKVRRQMEL 439

Homo_11 DQCNQFTRIEKQTKHMKKYPKRPDWNINKPPKRYIPASEKYPKQLQKQREEKKVRRQMEL 396

Homo_12 DQCNQFTRIEKQTKHMKKYPKRPDWNINKPPKRYIPASEKYPKQLQKQREEKKVRRQMEL 374

** ..:.***** *********** *****************************

Canis_I LNLVERNNPGHLSQNRGTSPVLP--SPQEAEARFRWHLIRKE-EPLKSDSFSKERSQ-SP 810

Canis_II LNLVERNNPGHLSQNRGTSPVLP--SPQEAEARFRWHLIRKE-EPLKSDSFSKERSQ-SP 777

Canis_III LNLVERNNPGHLSQNRGTSPVLP--SPQEAEARFRWHLIRKE-EPLKSDSFSKERSQ-SP 648

Canis_IV LNLVERNNPGHLSQNRGTSPVLP--SPQEAEARFRWHLIRKE-EPLKSDSFSKERSQ-SP 648

Canis_V LNLVERNNPGHLSQNRGTSPVLP--SPQEAEARFRWHLIRKE-EPLKSDSFSKERSQ-SP 710

Canis_VI LNLVERNNPGHLSQNRGTSPVLP--SPQEAEARFRWHLIRKE-EPLKSDSFSKERSQ-SP 677

Canis_VII LNLVERNNPGHLSQNRGTSPVLP--SPQEAEARFRWHLIRKE-EPLKSDSFSKERSQ-SP 789

Canis_VIII LNLVERNNPGHLSQNRGTSPVLP--SPQEAEARFRWHLIRKE-EPLKSDSFSKERSQ-SP 756

Homo_1 LHLVEKNNPGHLSQNRGISPEIFHSSHQETESKLRWHLVKKEEEPLNIHSFSKERSPSSP 798

Homo_2 LHLVEKNNPGHLSQNRGISPEIFHSSHQETESKLRWHLVKKEEEPLNIHSFSKERSPSSP 764

Homo_3 LHLVEKNNPGHLSQNRGISPEIFHSSHQETESKLRWHLVKKEEEPLNIHSFSKERSPSSP 804

Homo_4 LHLVEKNNPGHLSQNRGISPEIFHSSHQETESKLRWHLVKKEEEPLNIHSFSKERSPSSP 792

Homo_5 LHLVEKNNPGHLSQNRGISPEIFHSSHQETESKLRWHLVKKEEEPLNIHSFSKERSPSSP 791

Homo_6 LHLVEKNNPGHLSQNRGISPEIFHSSHQETESKLRWHLVKKEEEPLNIHSFSKERSPSSP 775

Homo_7 LHLVEKNNPGHLSQNRGISPEIFHSSHQETESKLRWHLVKKEEEPLNIHSFSKERSPSSP 765

Homo_8 LHLVEKNNPGHLSQNRGISPEIFHSSHQETESKLRWHLVKKEEEPLNIHSFSKERSPSSP 755

Homo_9 LHLVEKNNPGHLSQNRGISPEIFHSSHQETESKLRWHLVKKEEEPLNIHSFSKERSPSSP 748

Homo_10 LHLVEKNNPGHLSQNRGISPEIFHSSHQETESKLRWHLVKKEEEPLNIHSFSKERSPSSP 499

Homo_11 LHLVEKNNPGHLSQNRGISPEIFHSSHQETESKLRWHLVKKEEEPLNIHSFSKERSPSSP 456

Homo_12 LHLVEKNNPGHLSQNRGISPEIFHSSHQETESKLRWHLVKKEEEPLNIHSFSKERSPSSP 434

*:***:*********** ** : * **:*:::****::** ***: .******* **

Canis_I LELVKNRTQQTQT---------LKNRENLILGDSQTETSPGASEPSHFIPYVRTNEIYHL 861

Canis_II LELVKNRTQQTQT---------LKNRENLILGDSQTETSPGASEPSHFIPYVRTNEIYHL 828

Canis_III LELVKNRTQQTQT---------LKNRENLILGDSQTETSPGASEPSHFIPYVRTNEIYHL 699

Canis_IV LELVKNRTQQTQT---------LKNRENLILGDSQTETSPGASEPSHFIPYVRTNEIYHL 699

Canis_V LELVKNRTQQTQT---------LKNRENLILGDSQTETSPGASEPSHFIPYVRTNEIYHL 761

Canis_VI LELVKNRTQQTQT---------LKNRENLILGDSQTETSPGASEPSHFIPYVRTNEIYHL 728

Canis_VII LELVKNRTQQTQT---------LKNRENLILGDSQTETSPGASEPSHFIPYVRTNEIYHL 840

Canis_VIII LELVKNRTQQTQT---------LKNRENLILGDSQTETSPGASEPSHFIPYVRTNEIYHL 807

Homo_1 VPVVKNRTQQTQNTLHLPLKNSSYERENLISGSNQTELSSGISESSHFIPYVRTNEIYYL 858

Homo_2 VPVVKNRTQQTQNTLHLPLKNSSYERENLISGSNQTELSSGISESSHFIPYVRTNEIYYL 824

Homo_3 VPVVKNRTQQTQNTLHLPLKNSSYERENLISGSNQTELSSGISESSHFIPYVRTNEIYYL 864

Homo_4 VPVVKNRTQQTQNTLHLPLKNSSYERENLISGSNQTELSSGISESSHFIPYVRTNEIYYL 852

Homo_5 VPVVKNRTQQTQNTLHLPLKNSSYERENLISGSNQTELSSGISESSHFIPYVRTNEIYYL 851

Homo_6 VPVVKNRTQQTQNTLHLPLKNSSYERENLISGSNQTELSSGISESSHFIPYVRTNEIYYL 835

Homo_7 VPVVKNRTQQTQNTLHLPLKNSSYERENLISGSNQTELSSGISESSHFIPYVRTNEIYYL 825

Homo_8 VPVVKNRTQQTQNTLHLPLKNSSYERENLISGSNQTELSSGISESSHFIPYVRTNEIYYL 815

Homo_9 VPVVKNRTQQTQNTLHLPLKNSSYERENLISGSNQTELSSGISESSHFIPYVRTNEIYYL 808

Homo_10 VPVVKNRTQQTQNTLHLPLKNSSYERENLISGSNQTELSSGISESSHFIPYVRTNEIYYL 559

Homo_11 VPVVKNRTQQTQNTLHLPLKNSSYERENLISGSNQTELSSGISESSHFIPYVRTNEIYYL 516

Homo_12 VPVVKNRTQQTQNTLHLPLKNSSYERENLISGSNQTELSSGISESSHFIPYVRTNEIYYL 494

: :*********. :***** *..*** * * ** *************:*

Canis_I DPDAPLSRPLTQDLQYQNPHDCDQEQWQLFESDVRDPLLNPNLVKNRDRQQAILKGLSEL 921

Canis_II DPDAPLSRPLTQDLQYQNPHDCDQEQWQLFESDVRDPLLNPNLVKNRDRQQAILKGLSEL 888

Canis_III DPDAPLSRPLTQDLQYQNPHDCDQEQWQLFESDVRDPLLNPNLVKNRDRQQAILKGLSEL 759

Canis_IV DPDAPLSRPLTQDLQYQNPHDCDQEQWQLFESDVRDPLLNPNLVKNRDRQQAILKGLSEL 759

Canis_V DPDAPLSRPLTQDLQYQNPHDCDQEQWQLFESDVRDPLLNPNLVKNRDRQQAILKGLSEL 821

Canis_VI DPDAPLSRPLTQDLQYQNPHDCDQEQWQLFESDVRDPLLNPNLVKNRDRQQAILKGLSEL 788

Canis_VII DPDAPLSRPLTQDLQYQNPHDCDQEQWQLFESDVRDPLLNPNLVKNRDRQQAILKGLSEL 900

Canis_VIII DPDAPLSRPLTQDLQYQNPHDCDQEQWQLFESDVRDPLLNPNLVKNRDRQQAILKGLSEL 867

Homo_1 DPDAPLSGPSTQDPQYQNSQDCGQKRQLFDSDCVRDPLLNPNMVKNRDRQQAILKGLSEL 918

Homo_2 DPDAPLSGPSTQDPQYQNSQDCGQKRQLFDSDCVRDPLLNPNMVKNRDRQQAILKGLSEL 884

Homo_3 DPDAPLSGPSTQDPQYQNSQDCGQKRQLFDSDCVRDPLLNPNMVKNRDRQQAILKGLSEL 924

Homo_4 DPDAPLSGPSTQDPQYQNSQDCGQKRQLFDSDCVRDPLLNPNMVKNRDRQQAILKGLSEL 912

Homo_5 DPDAPLSGPSTQDPQYQNSQDCGQKRQLFDSDCVRDPLLNPNMVKNRDRQQAILKGLSEL 911

Homo_6 DPDAPLSGPSTQDPQYQNSQDCGQKRQLFDSDCVRDPLLNPNMVKNRDRQQAILKGLSEL 895

Homo_7 DPDAPLSGPSTQDPQYQNSQDCGQKRQLFDSDCVRDPLLNPNMVKNRDRQQAILKGLSEL 885

Homo_8 DPDAPLSGPSTQDPQYQNSQDCGQKRQLFDSDCVRDPLLNPNMVKNRDRQQAILKGLSEL 875

Homo_9 DPDAPLSGPSTQDPQYQNSQDCGQKRQLFDSDCVRDPLLNPNMVKNRDRQQAILKGLSEL 868

Homo_10 DPDAPLSGPSTQDPQYQNSQDCGQKRQLFDSDCVRDPLLNPNMVKNRDRQQAILKGLSEL 619

Homo_11 DPDAPLSGPSTQDPQYQNSQDCGQKRQLFDSDCVRDPLLNPNMVKNRDRQQAILKGLSEL 576

Homo_12 DPDAPLSGPSTQDPQYQNSQDCGQKRQLFDSDCVRDPLLNPNMVKNRDRQQAILKGLSEL 554

******* * *** **** :**.*:: : .. *********:*****************

Canis_I RQGLLQKQRELETNLMPLAANQEENFNSSF 951

Canis_II RQGLLQKQRELETNLMPLAANQEENFNSSF 918

Canis_III RQGLLQKQRELETNLMPLAANQEENFNSSF 789

Canis_IV RQGLLQKQRELETNLMPLAANQEENFNSSF 789

Canis_V RQGLLQKQRELETNLMPLAANQEENFNSSF 851

Canis_VI RQGLLQKQRELETNLMPLAANQEENFNSSF 818

Canis_VII RQGLLQKQRELETNLMPLAANQEENFNSSF 930

Canis_VIII RQGLLQKQRELETNLMPLAANQEENFNSSF 897

Homo_1 RQGLLQKQKELESSLLPLAENQEESFGSSF 948

Homo_2 RQGLLQKQKELESSLLPLAENQEESFGSSF 914

Homo_3 RQGLLQKQKELESSLLPLAENQEESFGSSF 954

Homo_4 RQGLLQKQKELESSLLPLAENQEESFGSSF 942

Homo_5 RQGLLQKQKELESSLLPLAENQEESFGSSF 941

Homo_6 RQGLLQKQKELESSLLPLAENQEESFGSSF 925

Homo_7 RQGLLQKQKELESSLLPLAENQEESFGSSF 915

Homo_8 RQGLLQKQKELESSLLPLAENQEESFGSSF 905

Homo_9 RQGLLQKQKELESSLLPLAENQEESFGSSF 898

Homo_10 RQGLLQKQKELESSLLPLAENQEESFGSSF 649

Homo_11 RQGLLQKQKELESSLLPLAENQEESFGSSF 606

Homo_12 RQGLLQKQKELESSLLPLAENQEESFGSSF 584

********:***:.*:*** ****.*.***

**Supplementary Data 4.** Alignment of the predicted canine wild-type and mutant isoforms I with mammal sequences, also shown in Figure 6.

Canis_lupus_WT MNLGDGLKLETELLDGKTKLILSPYECKSKNSVKMGSKNKIAKCPIRTKQTGYILKSTQN 60

Canis_lupus_Mut MNLGDGLKLETELLDGKTKLILSPYECKSKNSVKMGSKNKIAKCPIRTKQTGYILKSTQN 60

Homo_sapiens MNLGDGLKLETELLDGKTKLILSPYEHKSKISVKMGNKAKIAKCPLRTK-TGHILKSTQD 59

Felis_catus MNLGDGLKLETELLDGKTKLILSPYECKSKYSVKMGNKTKIVKCPLRTKQTGYILKSTQN 60

Bos_taurus MNLGDGLKLETELLNGKTKLILSPYDHKSKVSGKMGNKTKIAKYPLRTK-TGYILKS-QN 58

Equus_caballus MNLGDGLKLETELLDGKTKLILSPYEHKSKISVKMGNKTKIAKCPLRTKQTGYVLRSTQN 60

Mus_musculus MNLGDGLKLETELLDGKTKLILSPYEHKSKVSVKMGNKFKIAKCPLRTKQTGHTLKSTQN 60

Rattus_norvegicus MNLGDGLKLETELLDGKTKLILSPYEHKSKVSVKMGNKIKIAKYSLRTKQTGHTLKSTQN 60

Macaca_mulatta MNLGDGLKLETELLDGKTKLILSPYERKSKISVKMGNKTKIAKCPLRTK-TGHILKSTQD 59

**************:**********: *** * ***.* **.* :*** **: *:* *:

Canis_lupus_WT TCIRSGKLLQKKRMGSETSLAKGEKSSMIFSP-TKDLCKQYVDKDCLYVQKEISPATPTI 119

Canis_lupus_Mut TCIRSGKLLQKKRMGSETSLAKGEKSSMIFSP-TKDLCKQYVDKDCLYVQKEISPATPTI 119

Homo_sapiens TCIGSEKLLQKKPVGSETSQAKGEKNGMTFSS-TKDLCKQCIDKDCLHIQKEISPATPNM 118

Felis_catus TCIKSGKLLQKKRMGSETSQAKGEKNSMTFSP-TKDLCKQYVDKDCLYVQKEILPATPNI 119

Bos_taurus TCVRSEKFLQKKRIGSETSLLKGEENSMTFSP-TKDLCKQSVDKDSLHTEKEISFAPPNI 117

Equus_caballus TCVRSEKLLQKKRIGSETSLAEGEKNSMTFSP-TKDLCKQYADKDCLHIQKEISPATPNI 119

Mus_musculus TYIGNENLSQKKISTLDTSQAKPENSRLTFSPST---DKQYSEKDSVRVQKEISPTTSNI 117

Rattus_norvegicus TYIGSENLSQKKISTSDTSQAKRENSRLTFSSPSTDLCKQYSEKDCLRVQKEISPTASSI 120

Macaca_mulatta TCIGSEKLLQKKTVGSETSQAKGEKNGMTFSS-IKDLCKQCVDKDCLHIQKEIPPATPNI 118

* : . :: *** :** : *:. : ** ** :**.: :*** : .:

Canis_lupus_WT QKTRNT-INTSVVAKQKHCKKHITAENTKSGLVCLTQDQLQQILM-TVNQGNKSISAIEN 177

Canis_lupus_Mut QKTRNT-INTSVVAKQKHCKKHITAENTKSGLVCLTQDQLQQILM-TVNQGNKSISAIEN 177

Homo_sapiens QKTRNT-VNTSLVGKQKPHKKHITAENMKSSLVCLTQDQLQQILM-TVNQGNRSLSLTEN 176

Felis_catus QKTRNT-INTSIVSKEKLCQKHITAENMKSSLVCLTQDQLQQILM-TVNQGNGSISLIEN 177

Bos_taurus QKTRNT-MNPFVVAKQKPCRKHITAENRKSGLVCLTEDQLQRILM-TVNQGSKSITLTEN 175

Equus_caballus QKSRNT-INTSLVAKEKPCKKHITAENMKSGLVCLTQDQLQQILM-TVNQGNRSISLTEN 177

Mus_musculus RKIINT-TGTCPVAKQKPCKKNPTAETMNSGLVCLTQDQLRQILMLSVNQGNGSVCLTET 176

Rattus_norvegicus RKTVNTSTDTDPAAKQKPCRKPTAAEGMGSGLVCLTQDQLRQILMLSVNQGNGSMSLPEN 180

Macaca_mulatta QKTRNT-VNTSLVAKQKPHKKHITAENMKSSLVCLTQDQLQQILM-TVNQGNRSLSLTEN 176

:* ** . ..*:* :* :** *.*****:***::*** :****. *: *.

Canis_lupus_WT GKEET-SQDSLHLNNTSNQPKDENIMGVFQKNEALSSVLDENKSTLNKNQETSKQYEQKI 236

Canis_lupus_Mut GKEET-SQDSLHLNNTSNQPKDENIMGVFQKNEALSSVLDENKSTLNKNQETSKQYEQKI 236

Homo_sapiens GKEAKS-QYSLYLNSISNQPKDENIMGLFKKTEMVSSVPAENKSVLNEHQETSKQCEQKI 235

Felis_catus GREET-CRGSLHLNNISNQPNDENVMGVLQKTEALSSVLDESKSVLNKNQETSKQYEQKI 236

Bos_taurus EKEEETSKSQYSLNNIPDQPKDENIMGLLQNTETVSSIQDESKSVLNKNQATSNQCEQKI 235

Equus_caballus GKEEERNQYSLPLNNIPNQPKDKNIMGLVQKTEAVSSVQDENQSVLNKNQETSKQYEQKI 237

Mus_musculus GEEEA-SQDSLHLINIPSQPKDVNDTGFLQNTEAASPVTSEHEHVHRRAQEAFQQCEQKA 235

Rattus_norvegicus GEEVT-SQDSLHLISIPSQPKEVSVTGLLQKTEAVSPVARENEPVPQRAQAASQQCEQKA 239

Macaca_mulatta GKEAKS-QYSLHLNSISNQPKDENIMGLFKKTEMVSSVPAESKSVLNEHQDTSKQCEQKI 235

.* : . * . .**:: . *..::.* * : * : . .. * : :* ***

Canis_lupus_WT AIENVWKPADIFSTLGERERDRSLLEAKKAQWKKEL------DEQVALKKKEKEASEKWN 290

Canis_lupus_Mut AIENVWKPADIFSTLGERERDRSLLEAKKAQWKKEL------DEQVALKKKEKEASEKWN 290

Homo_sapiens AIENEWKPADIFSTLGERECDRSSLEAKKAQWRKEL------DEQVALKKKEKEVSEKWN 289

Felis_catus AIENAWKPADIFSTLGERERDRSLLEAKKAQWRKELGRTLFTDEQVALKKKEKEASEKWN 296

Bos_taurus STENVWKPADIFSTLGERERDRSLLEAKKAQWRKEL------DEQVALKKKEKEASEKWN 289

Equus_caballus ATENLWKPADIFSTLGERERDRSLLEAKKAQWRKEL------DEQVALKKKEKEASEKWN 291

Mus_musculus ATENEWKPADIFSTLGERERDKSLLEARRAQWKKEL------DEQVALKKKEKEASQKWH 289

Rattus_norvegicus TVESEWKPADIFSTLGERERDRSLLEARRAQWKKEL------DEQVALKKKEKEASQKWP 293

Macaca_mulatta AIENEWKPADIFSTLGERERDRSSLEAKKAQWRKEL------DEQVALKKKEKEVSEKWN 289

: *. ************** *:* ***::***:*** ************.*:**

Canis_lupus_WT NPWKKFESDKIVWEKFQTLGQSKTSLSSSNILSQSPSQITVVQADDYPLCRASQILEETV 350

Canis_lupus_Mut NPWKKFESDKIVWEKFQTLGQSKTSLSSSNILSQSPSQITVVQADDYPLCRASQILEETV 350

Homo_sapiens DPWKKSESDKIIWEKHQILDQSR----------------------------------ETV 315

Felis_catus NPWKKFESDKRVWEKFQILDQSKTCASSSSILSQSPCQVTVVQDDGHPLIRVGQILEDAV 356

Bos_taurus NPWKKSESDKIEWEKLQIVDQSK----------------------------------EAV 315

Equus_caballus NPWKKSESDKTVCEKLGIFDQSKTSASSSSVLSQSPIQVALIQADGHSLPRASQILEEAV 351

Mus_musculus NPWKPSD---IECEKSQVHDQSK----------------------------------EAR 312

Rattus_norvegicus DPWKPSE---ILCEKLQVLERSK----------------------------------EAG 316

Macaca_mulatta DPWKKSESDKIIWEKLQILDQSR----------------------------------ETV 315

:*** : ** :*: ::

Canis_lupus_WT PLERPLSTVKQEQQRKWIEDLNKQIEDDRQRKIEEKITSSKGEEHDRWAMHFDSLKNYPA 410

Canis_lupus_Mut PLERPLSTVKQEQQRKWIEDLNKQIEDDRQRKIEEKITSSKGEEHDRWAMHFDSLKNYPA 410

Homo_sapiens LLEHPFSAVKQELQRKWIEELNKQIEDDRQRKIEEKIIYSKGEEHDRWAMHFDSLKSYPG 375

Felis_catus PLEHSFNAVKQEQQRKWIEDLNKQIEDDRQRKTEEKITSSKGEEHDRWTVHFGSLKNYPA 416

Bos_taurus LLEQPFSAVKQEQQRKWIEELNKQIEDDRQKKAEEKIISSMAEERDRWAMHFDSLKNYPG 375

Equus_caballus PLGHPFSAVKQEQQRKWIEELNKQIEDDRQRKIEEKIISS-------------------- 391

Mus_musculus LLESPCSAIKQEQQRKWIEELNKQVEDDQQRKAEERMIYSKGEEHDRWAVHFDSLKSHPG 372

Rattus_norvegicus LLESPCITVKQEQQRKWIEELNKQVEDDQQRKAEEKLIYSKGEEHDRWAVHFDSFKSHPG 376

Macaca_mulatta LLEHPFSAVKQELQRKWIEELNKQIEDDRQRKIEEKIIYSKGEEHDRWAMHFDSLKSYPG 375

* ::*** ******:****:***:*:* **:: *

Canis_lupus_WT SQSQLSSRSIHNQPEYFCVSPDTQELSDISNVYTPTTGSQVEPSEEEHIAKPVRDMAMAN 470

Canis_lupus_Mut SQSQLSSRSIHNQPEYFCVSPDTQELSDISNVYTPTTGSQVEPSEEEHIAKPVRDMAMAN 470

Homo_sapiens SQSQLFSQSTHKQPEYFCVSPDTQELADVSSVCTPTTGSQVEPSEEEHIAKPIKDVVMAN 435

Felis_catus SQSQLSSRSTYNQPEYFCVSPDTQELADINSVYTPMSGSQVEPSEEEHTAKPLRDTTMAN 476

Bos_taurus SQSRLSSQSVQKQPEYFCVSPDTQELADISSLYTPTVGSQIEPSEEEHTVKPFRDTAVAN 435

Equus_caballus ---KLSSQSTHKQPEYFCVSPDTQELADISSVCTPTTGTHVEPSEEEHLVKPVGDVAMAN 448

Mus_musculus SQSRLSSQLTHQHLESLCVSPDTQELADVNGVFTPPPGVQAEPSEKEQRARPVLEMAVSH 432

Rattus_norvegicus SQSRLSSQLTPQHLESLCVSPDTQELADVSSVDTPPPAVQVKPSEKEQRARPVMDMSVSH 436

Macaca_mulatta SQSQLSSRSTHKQPEYFCVSPDTQELADVSSVCTPTTGSQVEPSEVEHRAKPIKDVVMAN 435

:* *: :: * :*********:*:..: ** . : :*** *: .:*. : :::

Canis_lupus_WT SQKTNFLRSMTALLDPAQIEERDRRRQKQLEHQKAITAQVEEKRRKKQLEEQQRKKEEQE 530

Canis_lupus_Mut SQKTNFLRSMTALLDPAQIEERDRRRQKQLEHQKAITAQVEEKRRKKQLEEQQRKKEEQE 530

Homo_sapiens SKKTNFLRSMTALLDPAQIEERDRRRQKQLEHQKAITAQVEEKRRKKQLEEEQRKKEEQE 495

Felis_catus SQKTNFLRSMTALLDPAQIEERDRRRQKQLEHQKAITAQVEEKRRKKQLEEQQRKKEEQE 536

Bos_taurus IQKTNFLRSMTALLDPAQIEERDRRRQKQLEHQKAITAQVEEKRKKKQLEEEQRKKEEQE 495

Equus_caballus SQKTNFLRSMTALLDPAEIEERDRRRQKQLEHQKAITAQVEEKRRKKQLEEEQRKKEEQE 508

Mus_musculus GPKTNFLRSMTALLDPAQIEERERRRQKQLEHQKAIMAQVEENRRKKRLEEEQRKKEEQE 492

Rattus_norvegicus GQKTNFLRSMTALLDPAQIEERERRRQKQLEHQKAITAQVEENRRKKRLEEEQRRKEEQE 496

Macaca_mulatta SKKTNFFRSMTALLDPAQIEERDRRRQKQLEHQKAITAQVEEKRRKKQLEEEQRKKEEQE 495

****:**********:****:************* *****:*:**:***:**:*****

Canis_lupus_WT EERRLAREREEMQKQYEEDILKQKQKEEIMTLKTNELFQTMQRAQELAQRLKQEQRIREL 590

Canis_lupus_Mut EERRLAREREEMQKQYEEDILKQKQKEEIMTLKTNELFQTMQRAQELAQRLKQEQRIREL 590

Homo_sapiens EELRLAQEREEMQKQYEEDILKQKQKEEIMTLKTNELFQTMQRAQELAQRLKQEQRIREL 555

Felis_catus EECRLAREREEMQKQYEEDILKQKQKEEIMALKTNELFQTMQRAQELAQRLKQEQRIREL 596

Bos_taurus EERRLAREREEMQKQYEEDILKQKQKEEMMTLQTNKLFQTMQRAQELAQRLKQEQRIREL 555

Equus_caballus EEHRLAREREEMQKQYEEDMFKQKQKEEIMTLKTNELFQTMQRAQELAQRLKQEQRIREL 568

Mus_musculus LELRLAREREEMQRQYEEDILKQRQREEIMTLKTNELFHTMQRAQELAQRLKQEQRIREL 552

Rattus_norvegicus EELRLAREREEMQRQYEEDILKQKHKEEIMTLKTNELFHTMQRAQELAQRLKQEQRIREL 556

Macaca_mulatta EELRLAQEREEMQKQYEEDILKQKQKEEIMTVKTNELFQTMQRAQELAQRLKQEQRIREL 555

* ***:******:*****::**:::**:*:::**:**:*********************

Canis_lupus_WT AQKGHDTSGLIKNLG------------------GYGLDDVSGKMNTCINSTTSPKKDTAV 632

Canis_lupus_Mut AQKGHDTSGLIKNLG------------------GYGLDDVSGKMNTCINSTTSPKKDTAV 632

Homo_sapiens AQKGHDTSRLIKNLGVDTIQMEYNAS-N-ISNSRHDSDEISGKMNTYMNSTT-SKKDTGV 612

Felis_catus AQKGHDTSGLIKNLG------------------GHGLDDVSDKMNICINSTTSPKKDTAV 638

Bos_taurus AQKGHDTSRLIQNLGIDTIQVEYNASTNNTGNSRHGLDKVSGKMNTYINSATSPRKDTGV 615

Equus_caballus AQKGHDTSRLIKNLGVDTIPVEYNASTNNISNSRHGLDEVSGKCNRCINSVISPKKDTGV 628

Mus_musculus AQKGHDTSRLIQNLGAQV---DYKAFTTI--SSSHSDPE-----ETADTSTASPKKDTGV 602

Rattus_norvegicus TQKGHDTSRLIQNLGAHV---DCKASTPV--SSSRDTEEAANDTRAAATSTASPKKDTGV 611

Macaca_mulatta AQKGHDTSRLIKNLGVDTIQMEYNASTN-ISNSRHDSDEVSGKMNTYTNYTT-SKKDTGV 613

:******* **:*** . . . . . :***.*

Canis_lupus_WT QTDDLNTGMFTIAESCCGSIIEREILNCSSPEIPAEFNDQF--KKDKQELINQDKAANLE 690

Canis_lupus_Mut QTDDLNTGMFTIAESCCGSIIEREILNCSSPEIPAEFNDQF--KKDKQELINQDKAANLE 690

Homo_sapiens QTDDLNIGIFTNAESHCGSLMERDITNCSSPEISAELIGQFSTKKNKQELT-QDKGASLE 671

Felis_catus QTDDLNTGMFTNAESCCGSVIEREIINCSSPEIPAEFSEQLHTKKDKQELTNQDKGANLE 698

Bos_taurus QTDDLNIGVFTNTESRCGSVSEREIINCSSPEIAAEFNEQFNTKNNKQELVSQIKGANLE 675

Equus_caballus QTDDLNTGIFTNAESCCGSVIEREIINCSSPEIPAEFNQQFNTKKNKQVVLSRDKGANLE 688

Mus_musculus QTDDVNLGIFNDALPPCGSVTEKGIRNISSPEISAEFSGQTDIRKENQELS-MNKGTNLD 661

Rattus_norvegicus QTDDVNLGIFNDGLPHCGSVTEWGVRNLSSPEISAEFSGQTGIRKEKQELS-MDKGTHLD 670

Macaca_mulatta QTDDLNIGIFTNAESHCGSLMERDLTNCSSPEILAELIGQFSTKKNKQELT-QDKGASLE 672

****:* *:*. ***: * : * ***** **: * ::::* : *.: *:

Canis_lupus_WT KENSWYNDQYE-FARTE--KKHMKKCPKRPDWNINKPLKRYIPASEKYPKQLQKQREEKK 747

Canis_lupus_Mut KENSWYNDQYE-FARTE--KKHMKKCPKRPDWNINKPLKRYIPASEKYPKQLQKQREEKK 747

Homo_sapiens KENNRCNDQCNQFTRIEKQTKHMKKYPKRPDWNINKPPKRYIPASEKYPKQLQKQREEKK 731

Felis_catus KENSWYNDQWNEFTRTEK-TKPVKKGPKRPDWNINKPLKRYIPASEKYPKQLQKQREEKK 757

Bos_taurus KENSWYNDQCNQITRREKQTKHMKKCPKRPDWNINKPLRRYIPASEKYPKQLQKLREEKK 735

Equus_caballus KENSWYNDQCNQFTRTEKQMKHMKKSPKRPDWNINKPIKRYIPASEKYPKQLQKQREEKK 748

Mus_musculus KENSWHNGQCNQYRRTEKQTKLMKKCPKKPAWNINKPLKKYVPASAKYPAHLQKEKEEKK 721

Rattus_norvegicus KENSWYNNRCTQHRRTEKQTKLVKKCPKKPAWNINKPLKRYVPASAKYPAHLQKEKEERK 730

Macaca_mulatta KENNRYNDQCNQFTRIEKQTKHMKKYPKRPDWNINKPPKRYIPASEKYPKQLQKQREEKK 732

***. *.: * * * :** **:* ****** ::*:*** *** :*** :**:*

Canis_lupus_WT VRRQMELLNLVERNNPGHLSQNRGTSPVL--PSPQEAEARFRWHLIRKE-EPLKSDSFSK 804

Canis_lupus_Mut SKKADGTA---------------------------------------------------- 755

Homo_sapiens VRRQMELLHLVEKNNPGHLSQNRGISPEIFHSSHQETESKLRWHLVKKEEEPLNIHSFSK 791

Felis_catus VRRQMELLNLVERNSPGHLSQNRGPSPVL--PSSQETEPRFRWHLIKKEEDPLKINSFSK 815

Bos_taurus VRRQMELLHLIERSNPGQISQNRGTSPEVLLSSHQETDPSFRRQLVRKEEEPLRTNCFSR 795

Equus_caballus VRRQMELLNLVEINNSGHLSQNRGTSPEVFHSSHQETEPRFKWHLVKKEEEPLKINSFSK 808

Mus_musculus VQRQMELLHLVERNNPENLSQNRGISP--LATSHRETESESRLHLIKKVEEPLKTPSVSK 779

Rattus_norvegicus VRRQMELLHLVQRNDPETLSQNNGASPDIFVSSHREAESEMRLHLLKKVEEPLET-SVSK 789

Macaca_mulatta VRRQMELLHLVEKNNPGHISQNRGISPEIFHSSHQETESKFRWHLVKKEEEPLNIHSFSK 792

::

Canis_lupus_WT ERSQS--PLELVKNRTQQTQ------TL-K---NRENLILGDSQTETSPGASEPSHFIPY 852

Canis_lupus_Mut ------------------------------------------------------------ 755

Homo_sapiens ERSPSS-PVPVVKNRTQQTQ-NTLHLPLKNSSYERENLISGSNQTELSSGISESSHFIPY 849

Felis_catus ERSPSPPPVPVVKNRTQQTQ------TL-NSNYERENLILEGSQTETSPGVSEPSHFIPY 868

Bos_taurus ERSQSP-PFPAVKSRTQQTQ------TLKNSNYERENLISGHSQTELSPGISDPFHFIPY 848

Equus_caballus ERSQSP-KVPAVNNRTQQTQ------TLKNTNYERENLISGGNQTATSPGISEPSHFIPY 861

Mus_musculus ERFQTS---PAVKNRTQQTQSNVLHLPLKNNDYEKETLTLGDGHTKLSDEMSEPSHFIPY 836

Rattus_norvegicus ERFQTS---PAVKSRTQQTQSNILHLPPKNSDYEKETLTLGDGHTKLSDERSEPSHFIPY 846

Macaca_mulatta ERSPSP-PVPAVKNRTQQTQ-NTLHLPLKNSSYERENLISGGNQTELSPGISESSHFIPY 850

Canis_lupus_WT VRTNEIYHLDPDAPLSRPLTQDLQYQNPHDCDQEQWQLFESDVRDPLLNPNLVKNRDRQQ 912

Canis_lupus_Mut ------------------------------------------------------------ 755

Homo_sapiens VRTNEIYYLDPDAPLSGPSTQDPQYQNSQDCGQKRQLFDSDCVRDPLLNPNMVKNRDRQQ 909

Felis_catus VRTSEIYYLDPDAPLSRPLTQDLQYQNPHDCDQEQGQLLDFGVRDPLLNPNLLKNRDRQQ 928

Bos_taurus VRTKEVYYLDPDAPLSRPVTQNPQYQNAHDR--EQELFASDHLRDPLLNPNLVKNRDRQQ 906

Equus_caballus VRTNEIYYLDPDAPLSRPLTQDPQYQNPYNYDQERQLVDSDHVRDPLLNPNLVKNRDRQQ 921

Mus_musculus VRTNEIYYLDPDAPLSRPSTQDNQYQKSHDCAREQELFDSDHIRDPLLNPKLVKNRDRQQ 896

Rattus_norvegicus VRTNEIYYLDPDAPLSRPSTQDNQYQKSHDCGGGQELFDSDHIRDPLLNPKLVKSRDRQQ 906

Macaca_mulatta VRTNEIYYLDPDAPLSQPSTQDPQYQNSQDCGQERQLFDSDCVRDPLLNPNMVKNRDRQQ 910

Canis_lupus_WT AILKGLSELRQGLLQKQRELETNLMPLAANQEENFNSSF 951

Canis_lupus_Mut --------------------------------------- 755

Homo_sapiens AILKGLSELRQGLLQKQKELESSLLPLAENQEESFGSSF 948

Felis_catus AILKGLSELRQGLLQKQRELETNLMPLAANQEENFNSSF 967

Bos_taurus AILKGLSDLRQGLLQKQRELETNLMPLAANQEENFSSSF 945

Equus_caballus AILKGLSELRQGLLQKQRELETNLMPLAANQEENFSSTF 960

Mus_musculus AILKGLSELRQGLLQKQKELETNLIPLTANQEDNFSSSF 935

Rattus_norvegicus AILRGLSELRQGLLQKQKELETNLIPLTANQEDNFSSSF 945

Macaca_mulatta AILKGLSELRQGLLQKQKELESSLLPLAENQEDNFGSSF 949

**Supplementary Data 5.** Alignment of the predicted canine wild-type and mutant CCDC66 for isoforms I and II.

**Isoform I**

WT_Canis_I MNLGDGLKLETELLDGKTKLILSPYECKSKNSVKMGSKNKIAKCPIRTKQTGYILKSTQN 60

Mut_Canis_I MNLGDGLKLETELLDGKTKLILSPYECKSKNSVKMGSKNKIAKCPIRTKQTGYILKSTQN 60

************************************************************

WT_Canis_I TCIRSGKLLQKKRMGSETSLAKGEKSSMIFSPTKDLCKQYVDKDCLYVQKEISPATPTIQ 120

Mut_Canis_I TCIRSGKLLQKKRMGSETSLAKGEKSSMIFSPTKDLCKQYVDKDCLYVQKEISPATPTIQ 120

************************************************************

WT_Canis_I KTRNTINTSVVAKQKHCKKHITAENTKSGLVCLTQDQLQQILMTVNQGNKSISAIENGKE 180

Mut_Canis_I KTRNTINTSVVAKQKHCKKHITAENTKSGLVCLTQDQLQQILMTVNQGNKSISAIENGKE 180

************************************************************

WT_Canis_I ETSQDSLHLNNTSNQPKDENIMGVFQKNEALSSVLDENKSTLNKNQETSKQYEQKIAIEN 240

Mut_Canis_I ETSQDSLHLNNTSNQPKDENIMGVFQKNEALSSVLDENKSTLNKNQETSKQYEQKIAIEN 240

************************************************************

WT_Canis_I VWKPADIFSTLGERERDRSLLEAKKAQWKKELDEQVALKKKEKEASEKWNNPWKKFESDK 300

Mut_Canis_I VWKPADIFSTLGERERDRSLLEAKKAQWKKELDEQVALKKKEKEASEKWNNPWKKFESDK 300

************************************************************

WT_Canis_I IVWEKFQTLGQSKTSLSSSNILSQSPSQITVVQADDYPLCRASQILEETVPLERPLSTVK 360

Mut_Canis_I IVWEKFQTLGQSKTSLSSSNILSQSPSQITVVQADDYPLCRASQILEETVPLERPLSTVK 360

************************************************************

WT_Canis_I QEQQRKWIEDLNKQIEDDRQRKIEEKITSSKGEEHDRWAMHFDSLKNYPASQSQLSSRSI 420

Mut_Canis_I QEQQRKWIEDLNKQIEDDRQRKIEEKITSSKGEEHDRWAMHFDSLKNYPASQSQLSSRSI 420

************************************************************

WT_Canis_I HNQPEYFCVSPDTQELSDISNVYTPTTGSQVEPSEEEHIAKPVRDMAMANSQKTNFLRSM 480

Mut_Canis_I HNQPEYFCVSPDTQELSDISNVYTPTTGSQVEPSEEEHIAKPVRDMAMANSQKTNFLRSM 480

************************************************************

WT_Canis_I TALLDPAQIEERDRRRQKQLEHQKAITAQVEEKRRKKQLEEQQRKKEEQEEERRLARERE 540

Mut_Canis_I TALLDPAQIEERDRRRQKQLEHQKAITAQVEEKRRKKQLEEQQRKKEEQEEERRLARERE 540

************************************************************

WT_Canis_I EMQKQYEEDILKQKQKEEIMTLKTNELFQTMQRAQELAQRLKQEQRIRELAQKGHDTSGL 600

Mut_Canis_I EMQKQYEEDILKQKQKEEIMTLKTNELFQTMQRAQELAQRLKQEQRIRELAQKGHDTSGL 600

************************************************************

WT_Canis_I IKNLGGYGLDDVSGKMNTCINSTTSPKKDTAVQTDDLNTGMFTIAESCCGSIIEREILNC 660

Mut_Canis_I IKNLGGYGLDDVSGKMNTCINSTTSPKKDTAVQTDDLNTGMFTIAESCCGSIIEREILNC 660

************************************************************

WT_Canis_I SSPEIPAEFNDQFKKDKQELINQDKAANLEKENSWYNDQYEFARTEKKHMKKCPKRPDWN 720

Mut_Canis_I SSPEIPAEFNDQFKKDKQELINQDKAANLEKENSWYNDQYEFARTEKKHMKKCPKRPDWN 720

************************************************************

WT_Canis_I INKPLKRYIPASEKYPKQLQKQREEKKVRRQMELLNLVERNNPGHLSQNRGTSPVLPSPQ 780

Mut_Canis_I INKPLKRYIPASEKYPKQLQKQREEKKSKKADGTA------------------------- 755

*************************** ::

WT_Canis_I EAEARFRWHLIRKEEPLKSDSFSKERSQSPLELVKNRTQQTQTLKNRENLILGDSQTETS 840

Mut_Canis_I ------------------------------------------------------------ 755

WT_Canis_I PGASEPSHFIPYVRTNEIYHLDPDAPLSRPLTQDLQYQNPHDCDQEQWQLFESDVRDPLL 900

Mut_Canis_I ------------------------------------------------------------ 755

WT_Canis_I NPNLVKNRDRQQAILKGLSELRQGLLQKQRELETNLMPLAANQEENFNSSF 951

Mut_Canis_I --------------------------------------------------- 755

**Isoform II**

WT_Canis_II MMGSKNKIAKCPIRTKQTGYILKSTQNTCIRSGKLLQKKRMGSETSLAKGEKSSMIFSPT 60

Mut_Canis_II MMGSKNKIAKCPIRTKQTGYILKSTQNTCIRSGKLLQKKRMGSETSLAKGEKSSMIFSPT 60

************************************************************

WT_Canis_II KDLCKQYVDKDCLYVQKEISPATPTIQKTRNTINTSVVAKQKHCKKHITAENTKSGLVCL 120

Mut_Canis_II KDLCKQYVDKDCLYVQKEISPATPTIQKTRNTINTSVVAKQKHCKKHITAENTKSGLVCL 120

************************************************************

WT_Canis_II TQDQLQQILMTVNQGNKSISAIENGKEETSQDSLHLNNTSNQPKDENIMGVFQKNEALSS 180

Mut_Canis_II TQDQLQQILMTVNQGNKSISAIENGKEETSQDSLHLNNTSNQPKDENIMGVFQKNEALSS 180

************************************************************

WT_Canis_II VLDENKSTLNKNQETSKQYEQKIAIENVWKPADIFSTLGERERDRSLLEAKKAQWKKELD 240

Mut_Canis_II VLDENKSTLNKNQETSKQYEQKIAIENVWKPADIFSTLGERERDRSLLEAKKAQWKKELD 240

************************************************************

WT_Canis_II EQVALKKKEKEASEKWNNPWKKFESDKIVWEKFQTLGQSKTSLSSSNILSQSPSQITVVQ 300

Mut_Canis_II EQVALKKKEKEASEKWNNPWKKFESDKIVWEKFQTLGQSKTSLSSSNILSQSPSQITVVQ 300

************************************************************

WT_Canis_II ADDYPLCRASQILEETVPLERPLSTVKQEQQRKWIEDLNKQIEDDRQRKIEEKITSSKGE 360

Mut_Canis_II ADDYPLCRASQILEETVPLERPLSTVKQEQQRKWIEDLNKQIEDDRQRKIEEKITSSKGE 360

************************************************************

WT_Canis_II EHDRWAMHFDSLKNYPASQSQLSSRSIHNQPEYFCVSPDTQELSDISNVYTPTTGSQVEP 420

Mut_Canis_II EHDRWAMHFDSLKNYPASQSQLSSRSIHNQPEYFCVSPDTQELSDISNVYTPTTGSQVEP 420

************************************************************

WT_Canis_II SEEEHIAKPVRDMAMANSQKTNFLRSMTALLDPAQIEERDRRRQKQLEHQKAITAQVEEK 480

Mut_Canis_II SEEEHIAKPVRDMAMANSQKTNFLRSMTALLDPAQIEERDRRRQKQLEHQKAITAQVEEK 480

************************************************************

WT_Canis_II RRKKQLEEQQRKKEEQEEERRLAREREEMQKQYEEDILKQKQKEEIMTLKTNELFQTMQR 540

Mut_Canis_II RRKKQLEEQQRKKEEQEEERRLAREREEMQKQYEEDILKQKQKEEIMTLKTNELFQTMQR 540

************************************************************

WT_Canis_II AQELAQRLKQEQRIRELAQKGHDTSGLIKNLGGYGLDDVSGKMNTCINSTTSPKKDTAVQ 600

Mut_Canis_II AQELAQRLKQEQRIRELAQKGHDTSGLIKNLGGYGLDDVSGKMNTCINSTTSPKKDTAVQ 600

************************************************************

WT_Canis_II TDDLNTGMFTIAESCCGSIIEREILNCSSPEIPAEFNDQFKKDKQELINQDKAANLEKEN 660

Mut_Canis_II TDDLNTGMFTIAESCCGSIIEREILNCSSPEIPAEFNDQFKKDKQELINQDKAANLEKEN 660

************************************************************

WT_Canis_II SWYNDQYEFARTEKKHMKKCPKRPDWNINKPLKRYIPASEKYPKQLQKQREEKKSKKADG 720

Mut_Canis_II SWYNDQYEFARTEKKHMKKCPKRPDWNINKPLKRYIPASEKYPKQLQKQREEKKVRRQME 720

****************************************************** ::

WT_Canis_II TA---------------------------------------------------------- 722

Mut_Canis_II LLNLVERNNPGHLSQNRGTSPVLPSPQEAEARFRWHLIRKEEPLKSDSFSKERSQSPLEL 780

WT_Canis_II ------------------------------------------------------------ 722

Mut_Canis_II VKNRTQQTQTLKNRENLILGDSQTETSPGASEPSHFIPYVRTNEIYHLDPDAPLSRPLTQ 840

WT_Canis_II ------------------------------------------------------------ 722

Mut_Canis_II DLQYQNPHDCDQEQWQLFESDVRDPLLNPNLVKNRDRQQAILKGLSELRQGLLQKQRELE 900

WT_Canis_II ------------------ 722

Mut_Canis_II TNLMPLAANQEENFNSSF 918
